# Prodrug Strategies for the Development of β-l-5-((*E*)-2-Bromovinyl)-1-((2*S*,4*S*)-2-(hydroxymethyl)-1,3-(dioxolane-4-yl))uracil (l-BHDU) against Varicella Zoster Virus (VZV)

**DOI:** 10.1021/acs.jmedchem.3c00545

**Published:** 2023-05-04

**Authors:** Uma S. Singh, Ananda K. Konreddy, Yugandhar Kothapalli, Dongmei Liu, Megan G. Lloyd, Vidya Annavarapu, Catherine A. White, Michael. G. Bartlett, Jennifer F. Moffat, Chung K. Chu

**Affiliations:** † Department of Pharmaceutical and Biomedical Science, College of Pharmacy, University of Georgia, Athens, Georgia 30602, United States; ‡ State University of New York, Upstate Medical University, Syracuse, New York 13210, United States

## Abstract

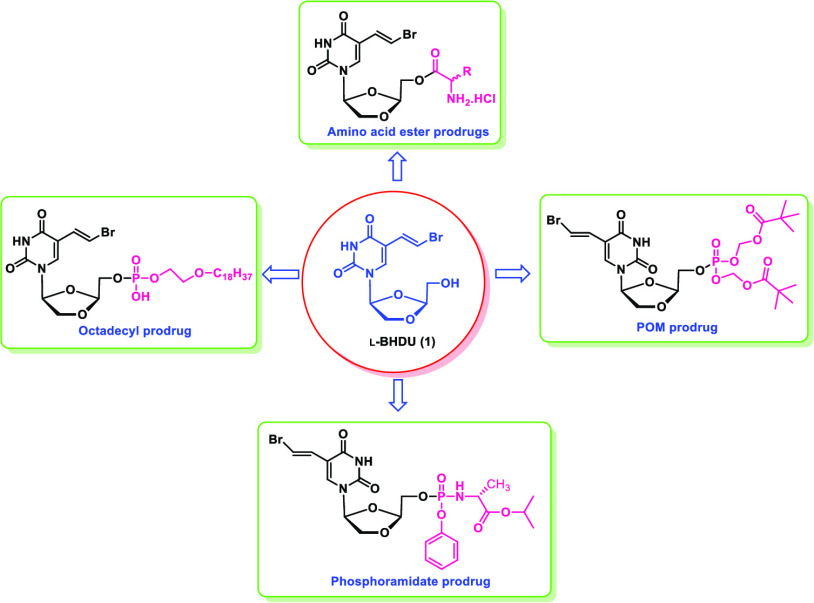

Varicella zoster virus (VZV) establishes lifelong infection after primary disease and can reactivate. Several drugs are approved to treat VZV diseases, but new antivirals with greater potency are needed. Previously, we identified β-l-5-((*E)*-2-bromovinyl)-1-((2*S*,4*S*)-2-(hydroxymethyl)-1,3-(dioxolane-4-yl))uracil (l-BHDU, **1**), which had significant anti-VZV activity. In this communication, we report the synthesis and evaluation of numerous l-BHDU prodrugs: amino acid esters (**14**–**26**), phosphoramidates (**33**–**34**), long-chain lipids (ODE-l-BHDU-MP, **38**, and HDP-l-BHDU-MP, **39**), and phosphate ester prodrugs (POM-l-BHDU-MP, **41**, and POC-l-BHDU-MP, **47**). The amino acid ester l-BHDU prodrugs (l-phenylalanine, **16**, and l-valine, **17**) had a potent antiviral activity with EC_50_ values of 0.028 and 0.030 μM, respectively. The phosphate ester prodrugs POM-l-BHDU-MP and POC-l-BHDU-MP had a significant anti-VZV activity with EC_50_ values of 0.035 and 0.034 μM, respectively, and no cellular toxicity (CC_50_ > 100 μM) was detected. Out of these prodrugs, ODE-l-BHDU-MP (**38**) and POM-l-BHDU-MP (**41**) were selected for further evaluation in future studies.

## Introduction

Varicella zoster virus (VZV), an alphaherpesvirus, causes chickenpox (varicella) on primary infection and shingles (zoster) upon reactivation from latency.^1^ Currently, vaccination is available with a live attenuated strain for both stages of VZV disease,^2^ and an adjuvant subunit vaccine is approved to prevent shingles.^3^ While pediatric vaccination has reduced the incidence of chickenpox, shingles remains prevalent. According to the Centers for Disease Control and Prevention,^4^ there are an estimated one million cases of zoster annually in the U.S. Those at highest risk are people over the age of 50, transplant recipients, people living with human immunodeficiency virus (HIV), and anyone who is immunocompromised. When VZV reactivates in the skin, it causes a painful, vesicular rash that contains abundant infectious virus. Antiviral therapy is most effective when given within three days of the appearance of the rash, and there is evidence that prompt antiviral treatment can reduce acute pain, speed healing, reduce virus shedding, and lower the incidence of herpetic neuropathic pain.^5^ A major complication of shingles is postherpetic neuralgia, which is a neuropathic pain that persists for months to years after the rash heals.^6^ There is a compelling need for improved antivirals for the treatment of VZV infections that are more effective and safe.

**Figure 1:**
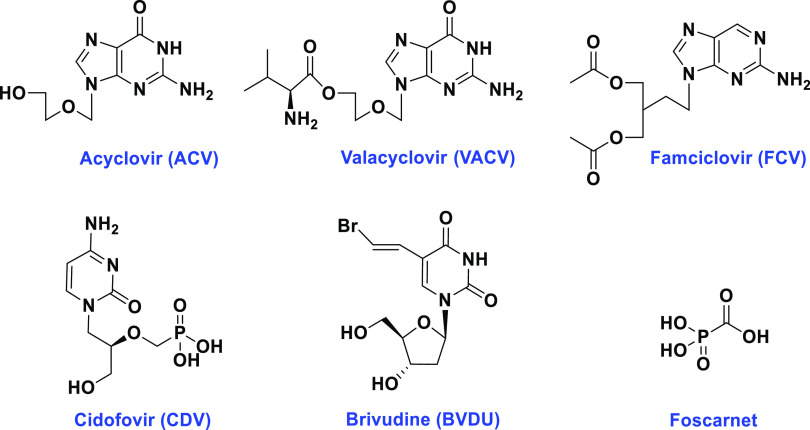
Structure of antiviral drugs for the treatment of VZV.

Currently, several nucleoside analogues are used to treat VZV infections, including acyclovir (ACV), valacyclovir (VACV), and famciclovir (FCV) (Figure 1).^7^ These nucleoside analogues act on the viral DNA polymerase to disrupt viral DNA synthesis. They are active in their triphosphate form, requiring the VZV thymidine kinase (TK) and cellular enzymes for activation.^8^ These drugs are not highly effective against VZV, large doses are required, and long-term use is associated with the development of drug resistance.^9^ Cidofovir (CDV, Figure 1), a broad-spectrum nucleotide analogue, is active against VZV. Unfortunately, nephrotoxicity and lack of oral bioavailability restrict CDV use as a first-line treatment. In selected cases, CDV may be used off-label to treat acyclovir-resistant VZV.^8^ Foscarnet (pyrophosphate analogue, Figure 1) is another second-line treatment; however, it is associated with many deleterious side effects.^10^

Another class of compounds discovered to treat VZV infections is the cyclic derivatives of uridine. Trifluridine and idoxuridine are used primarily as topical treatments for herpes zoster ophthalmicus.^11^ The bromovinyl analogue, brivudine (BVDU, *E*-5-(2-bromovinyl)-2′-deoxy uridine, Figure 1), has been approved in Europe for the treatment of VZV infections and has potent anti-herpes activity.^12^ Similar to the nucleoside analogues, BVDU must be converted by the viral TK into the 5′-monophosphate and diphosphate forms. Cellular kinases then perform the final conversion step into the active 5′-triphosphate form (BVDU-TP). BVDU-TP selectively interacts with the viral DNA polymerase as a competitive inhibitor, where it is incorporated into viral DNA, leading to chain termination. BVDU has a better anti-VZV activity profile than acyclovir or its prodrug, thus requiring a smaller dose. Additionally, BVDU can be administered orally once a day, making it more appealing than other drugs used for the treatment of VZV infections. The major drawback associated with BVDU is that it is catabolized in the liver into bromovinyluracil (BVU).^13^ BVU inhibits dihydropyrimidine dehydrogenase (DPD), which degrades thymidine and uracil. The anticancer drug 5-fluorouracil (5-FU) is catabolized by DPD, thus causing a harmful drug interaction with brivudine.^14^ Due to the adverse effects associated with currently prescribed drugs to treat VZV infections, there is a critical need for new antivirals that are safe and effective against VZV and its resistant strains.

We reported a uridine derivative, β-l-5-((*E)*-2-bromovinyl)-1-((2*S*,4*S*)-2-(hydroxymethyl)-1,3-(dioxolane-4-yl))uracil (l-BHDU), as a potent and safe anti-VZV agent.^15^ In human foreskin fibroblasts (HFFs), l-BHDU was effective against VZV with an EC_50_ value of 0.25 μM. It was not cytotoxic in HFFs up to 200 μM, yielding a selectivity index (SI) of >909.^16^*In vivo*, l-BHDU significantly reduced VZV growth and spread compared to ACV and VACV. Metabolic studies indicated that 5-FU did not accumulate in mice treated with l-BHDU.^16^ It is likely that l-BHDU does not inhibit DPD and would have a better safety profile than brivudine. To increase cellular bioavailability and uptake of l-BHDU, we developed an amino acid ester prodrug, l-BHDU-l-valine, which had enhanced anti-VZV activity compared to its parent molecule. l-BHDU-l-valine had an EC_50_ value of 0.03 μM with a CC_50_ of >200 μM.^16^ Encouraged by these findings and to enhance the pharmacokinetic (PK) profile of l-BHDU, we explored various prodrug approaches to improve the potency of l-BHDU against VZV. Here, we describe the synthesis and antiviral evaluation of various l-BHDU prodrugs, including thirteen 5′-amino acid ester prodrugs (**14**–**26**), two phosphoramidate prodrugs (**33** & **34**), two long-chain phospholipid prodrugs, an octadecyloxyethyl prodrug of l-BHDU monophosphate (ODE-l-BHDU-MP, **38**) and a hexadecyloxypropyl prodrug of l-BHDU monophosphate (HDP-l-BHDU-MP, **39**), and two phosphate ester prodrugs (POM-l-BHDU-MP, **41**, and POC-l-BHDU-MP, **47**).

## Results and Discussion

The development of 5′-amino acid ester prodrugs based on nucleoside analogues is well-established and known to reduce cytotoxicity.^17^ We previously showed that inserting an amino acid as part of the prodrug strategy enhanced bioavailability and carrier-mediated cell transport while lowering the polarity of the standard nucleoside.^18^ Furthermore, the phosphoramidate, phosphate esters, and long-chain phospholipid prodrugs are all known to increase the antiviral potency of nucleosides while overcoming the rate-limiting first step, monophosphorylation. Chemical and enzymatic mechanisms release nucleoside monophosphate (NMP).^19^ For instance, the 1-*O*-hexadecyloxypropyl and 1-*O*-octadecyloxyethyl prodrugs of CDV are more active against DNA viruses than the parent molecule.^20^ Due to their lipophilic nature, phospholipid prodrugs exhibit enhanced cellular uptake and oral bioavailability, and they inhibit viral replication more efficiently than standard CDV.^21^ However, VZV infects many human cell types and establishes latency in nerve cells. Unfortunately, l-BHDU was not lipophilic enough to penetrate nerve cells; thus, we synthesized the octadecyloxyethyl-l-BHDU-MP (ODE-l-BHDU-MP, **38**) and hexadecyloxypropyl-l-BHDU-MP (HDP-l-BHDU-MP, **39**) prodrugs. Esterification of l-BHDU-MP with octadecyloxyethyl (ODE) or hexadecyloxypropyl (HDP) was performed to increase its bioavailability and cell penetration, as they resemble phospholipids of the cell membrane. It was expected that the addition of these long hydrocarbon chains would improve the uptake and intracellular transport of the compounds.

To date, the FDA has approved adefovir dipivoxil [bis(pivaloyloxymethyl), POM]^22,23^ and tenofovir disoproxil fumarate [bis(isopropyloxymethyl carbonate), POC]^24^ for the treatment of hepatitis B virus (HBV) and human immunodeficiency virus (HIV) infections, respectively. The lipophilic nature of POM or POC groups may enhance the bioavailability and cellular uptake of l-BHDU.^25^ During the metabolism of adefovir and tenofovir prodrugs, the first POM ester group is degraded and forms an unstable hydroxymethyl alcoholate intermediate that undergoes chemical rearrangement and releases formaldehyde. Next, the second POM ester group is cleaved to generate free NMP.^26^ Similarly, POC prodrugs are also metabolized by enzymatic degradation. Carbonates of POC are decomposed by esterase to produce an unstable carboxylate intermediate that results in the sequential release of carbon dioxide and formaldehyde to produce a free NMP.^27^ Following these strategies, we synthesized POM-l-BHDU-MP (**41**) and POC-l-BHDU-MP (**47**) prodrugs for evaluation against VZV.

### Chemistry

**Scheme 1:**
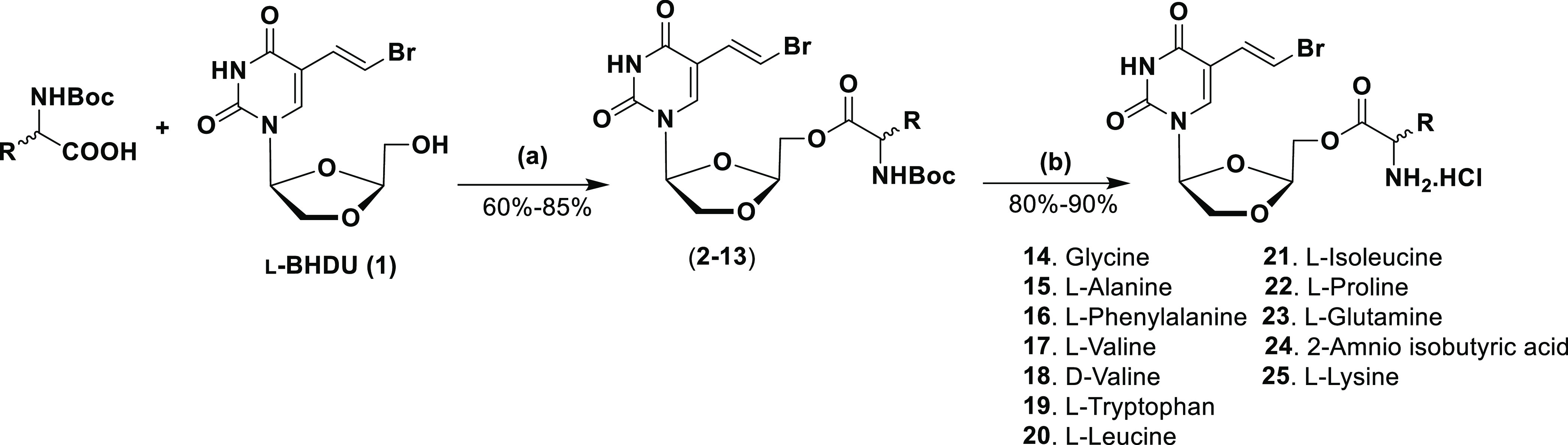
Synthesis of 5′-*O*-Amino Acid Ester Prodrugs of l-BHDU Reagents and conditions: (a) DIC, DMAP, DCM, rt, 12 h; (b) 2 M TFA in DCM, 0 °C to rt, 1 h; 1 M HCl in ether, MeOH, 0 °C to rt, 1 h.

Synthesis of l-BHDU was performed as per our reported standard protocol.^15^ The chemical structures of the newly synthesized amino acid ester prodrugs of l-BHDU (**14**–**25**) are shown in Scheme 1. To synthesize the targeted amino acid ester prodrugs (**14**–**25**), condensation of l-BHDU was carried out with appropriate *Boc*-protected d- or l- amino acids. l-BHDU was stirred with the Boc-protected amino acids in the presence of catalytic 4-(dimethylamino)pyridine (DMAP) and the coupling agent 1,3-diisopropylcarbodiimide (DIC) at room temperature (rt) to produce a coupled intermediate (**2**–**13**) (60–85% yield). The *Boc* group was removed from compounds **2**–**13** by treatment with 2 M trifluoro acetic acid (TFA) in dichloromethane (DCM), followed by 1 M HCl solution in the ether, resulting in a targeted amino acid ester HCl salt (**14**–**25**) of l-BHDU (80–90% yield).

**Scheme 2:**
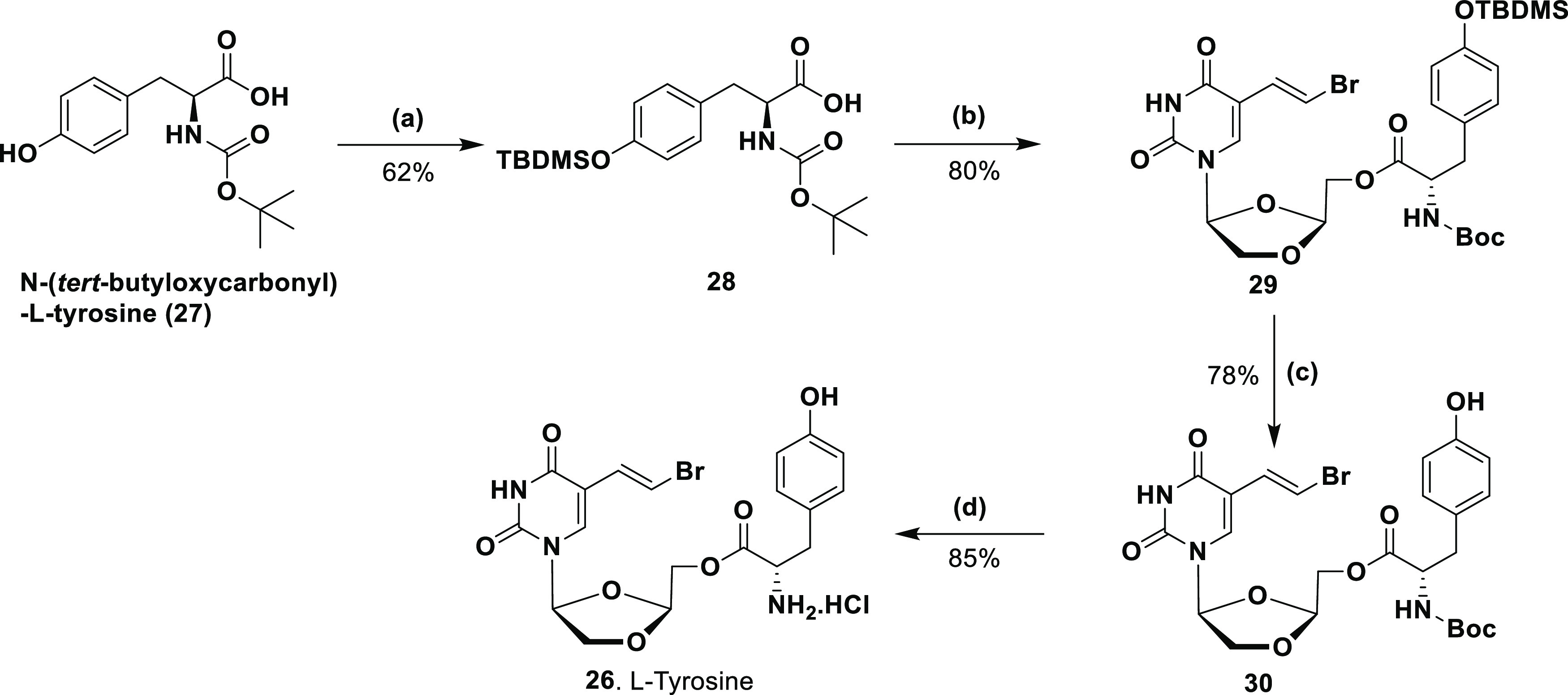
Synthesis of 5′-*O*-l-Amino Acid Tyrosine Ester Prodrugs of l-BHDU Reagents and conditions: (a) TBDMSCl, imidazole, DMF, rt, 16 h; (b) l-BHDU, DIC, DMAP, DCM, rt, 12 h; (c) 1 M TBAF solution in THF, THF, 0 °C—rt, 3 h; (d) 2 M TFA in DCM; 2 M HCl in ether, MeOH, 0 °C—rt; 1.5 h.

To synthesize the tyrosine amino acid ester l-BHDU prodrug (Scheme 2), we started with the *N*-Boc-protected l-tyrosine amino acid (**27**), where the phenol of tyrosine was protected with the *tert*-butyldimethylsilyl chloride (TBDMSCl) in the presence of imidazole in *N*,*N*-dimethylformamide (DMF) at rt to give intermediate **28** (62% yield). Intermediate **28** was then condensed with l-BHDU *via* a coupling reaction in the presence of DIC at rt to afford the coupled product **29** (80% yield). Next, TBDMS deprotection of **29** was performed in a 1 M tetrabutylammonium fluoride (TBAF) solution in tetrahydrofuran (THF) to produce intermediate **30** (78% yield). Finally, deprotection of Boc was performed by treating intermediate **30** with 2 M TFA solution in DCM followed by treatment with 2 M HCl solution in ether to produce l-BHDU-l-tyrosine amino acid ester as HCl salt, **26** (85% yield).

**Scheme 3:**
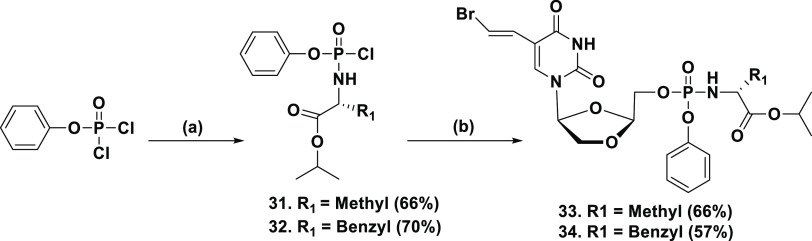
Synthesis of Phosphoramidate Prodrugs of l-BHDU Reagents and conditions: (a) l-alanine isopropyl ester hydrochloride or l-phenylalanine isopropyl ester hydrochloride, Et_3_N, DCM, −78 °C—rt, 3 h; (b) l-BHDU (1); NMI, THF 0 °C—rt, 12 h.

To bypass first-step rate-limiting monophosphorylation, McGuigan introduced the concept of phosphoramidate prodrugs consisting of an amino ester moiety attached *via* a P–N bond formation to a nucleoside aryl phosphate.^28^ Mechanistically, aryloxy phosphoramidate prodrugs release NMP intracellularly by chemical and enzymatic degradation. Phosphoramidate prodrugs have enhanced pharmacokinetic properties with better cellular uptake and bioavailability. Following this strategy, phosphoramidate prodrugs of l-BHDU were prepared according to our reported protocol.^29^ First, phosphorochloridate intermediates of the aryl alkoxy-amino ester (**31** & **32**) were synthesized. Phenyl dichlorophosphate was treated with l-alanine isopropyl ester hydrochloride or l-phenylalanine isopropyl ester hydrochloride in the presence of triethylamine (Et_3_N) in DCM at −78 °C to render intermediates **31** and **32**, respectively (60–70% yield, Scheme 3). Next, condensation of phosphorochloridate intermediates (**31** & **32**) was carried out with l-BHDU in the presence of *N*-methylimidazole (NMI) in THF at 0 °C to rt to furnish phosphoramidate prodrugs **33** & **34** (50–65% yield).

**Scheme 4:**
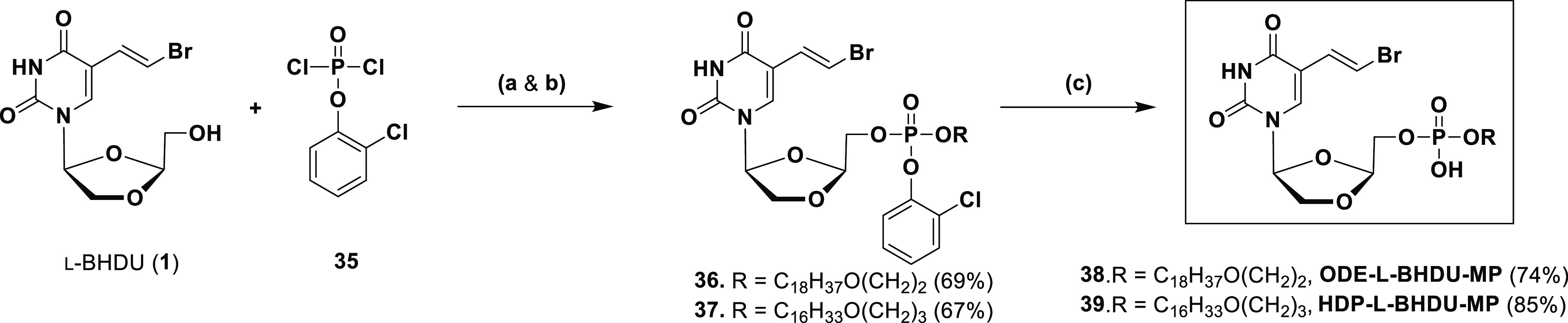
Synthesis of Long-Chain Lipid Phosphate Prodrugs of l-BHDU Reagents and conditions: (a) 1,2,4-triazole, Et_3_N, THF, rt, 1.5 h; (b) ROH, NMI, THF, rt, 12 h; (c) 0.5 N NaOH, THF/H_2_O, 50 °C, 2 h.

The octadecyloxyethyl prodrug of l-BHDU monophosphate (ODE-l-BHDU-MP, **38**) and the hexadecyloxypropyl prodrug of l-BHDU monophosphate (HDP-l-BHDU-MP, **39**) prodrugs were synthesized according to our standard protocol.^30^ These long-chain lipid phosphates of l-BHDU monophosphate (l-BHDU-MP) were constructed using a phosphotriester approach (Scheme 4). First, l-BHDU was condensed with 2-chlorophenyl dichlorophosphate (**35**) in the presence of 1,2,4-triazole and Et_3_N to yield the coupled intermediate. *In situ*, without further purification, the intermediate was treated with the appropriate long-chain lipid alcohol (3-hexadecyloxy-1-propanol or 2-octadecyloxy-1-ethanol) in the presence of NMI in THF at rt to afford a fully protected corresponding intermediate (**36** & **37**, 67–69% yield). The phosphotriester intermediates **36** & **37** had two closely distinguished signals in the ^31^P NMR, corresponding to the two diastereoisomers. This was also apparent from the ^1^H NMR spectroscopy. Next, the 2-chlorophenyl group of each phosphotriester **36** & **37** was removed using 0.5 N NaOH in THF at 50 °C to yield prodrugs **38** and **39** (74–85% yield).

**Scheme 5:**
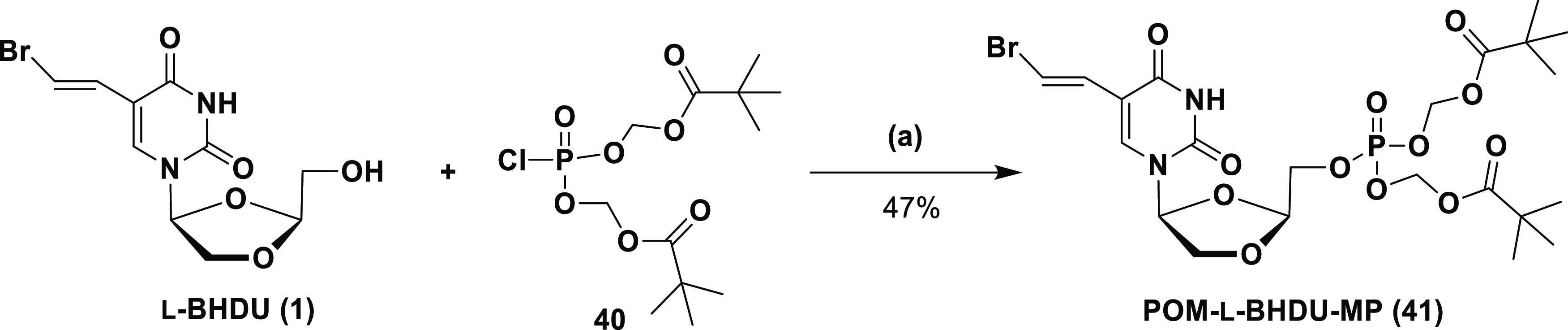
Synthesis of the POM-l-BHDU-MP Prodrug Reagents and conditions: (a) NMI, THF, 0 °C to rt, 3 h.

To synthesize POM-l-BHDU-MP, first, the synthesis of bis(POM)phosphorochloridate (**40**) was carried out according to the reported protocol by Hawang et al.^31^ Next, coupling of l-BHDU was performed with POM chloride (**40**), in the presence of NMI in THF to give POM-l-BHDU-MP (**41**) (47% yield, Scheme 5).

**Scheme 6:**
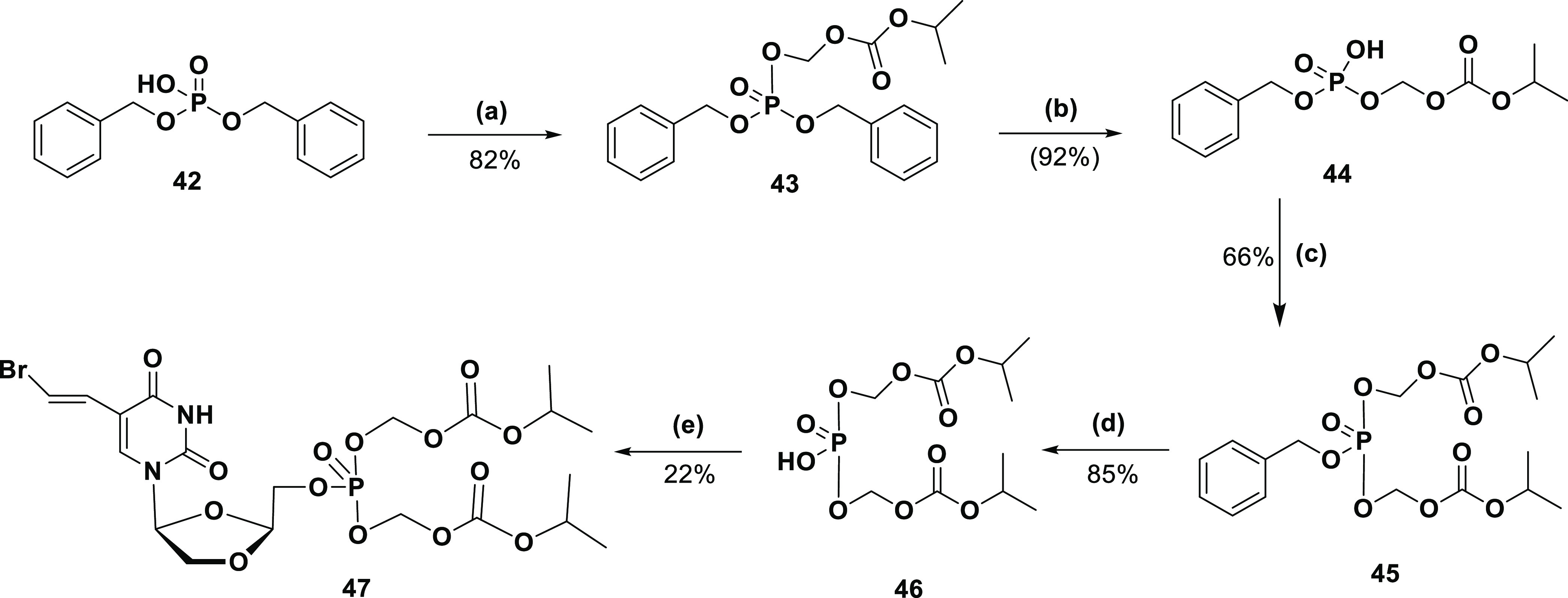
Synthesis of the Bis-POC-l-BHDU-MP Prodrug Reagents and conditions: (a) POC-I, Cs_2_CO_3_, acetone, rt, 24 h; (b) NaI, acetonitrile, rt, 24 h; (c) POC-I, Cs_2_CO_3_, acetone, rt, 24 h; (d) Pd/C, 5-10 psi, rt, 2 h; (e) l-BHDU, BOP-Cl, 3-nitro-1, 2, 4-triazole, DIPEA, THF, rt, 2–3 h.

The synthesis of the bis-POC-l-BHDU-MP (**47**) prodrug was commenced with compound **42** (Scheme 6). Alkylation of **42** was carried out with the POC-I and cesium carbonate (Cs_2_CO_3_) in THF to yield POC alkylated ester **43** (82% yield). Initially, we attempted to convert **43** to **44** using the hydrogenation conditions on Pd/C. Unfortunately, selective monobenzyl deprotection was not achieved by this method, and a major didebenzylated product was obtained. Thus, selective monobenzyl deprotection was performed with LiBr, but only a small amount of compound **44** was formed (7–9% yield). Therefore, the selective monobenzyl deprotection reaction was again tried with sodium iodide (NaI) in acetonitrile, and in this case, it exclusively produced **44** in 92% yield. Repeated alkylation of **44** was performed with POC-I and Cs_2_CO_3_ in acetone to give intermediate **45** (66% yield). The final benzyl deprotection of **45** was executed on Pd/C in hydrogen at 5 psi to give the key intermediate **46** (85% yield). Finally, l-BHDU was treated with **46** in THF in the presence of *N,N*-diisopropylethylamine (DIPEA), bis(2-oxo-3-oxazolidinyl) phosphinic chloride (BOP-Cl), and 3-nitro-1,2,4-triazole to produce POC-l-BHDU-MP (**47**) (22% yield). The structures of the all-synthesized prodrugs were confirmed by ^1^H NMR, ^13^C NMR, ^31^P NMR, and electrospray ionization ESI high-resolution mass spectra (EI-HRMS).

### Antiviral Activity

We previously reported that l-BHDU had good antiviral activity against VZV.^16^ We found that l-BHDU effectively reduced VZV replication at 15 mg/kg/day in the SCID-hu mouse with human fetal skin xenografts. It was well tolerated up to 150 mg/kg in mice and was not toxic to cells or skin.^16^ In the same study, ACV or VACV had no effect on VZV spread at 120 mg/kg/day. Others have corroborated these results, showing that VACV did not affect VZV replication in SCID-hu mice.^32^ In our previous study, we also measured l-BHDU in various mouse organs, finding that it reached high levels in most tissues but did not penetrate the brain.

The antiviral activity of l-BHDU depends on phosphorylation by VZV thymidine kinase (TK), and most antiviral resistance maps to the TK gene.^33^ To avoid the dependence on VZV TK for the generation of l-BHDU-MP, we utilized the phosphate ester, phosphoramidate, and long-chain phospholipid prodrug strategies. We expected these techniques to enhance the cell-bioavailability, tissue distribution, and pharmacokinetic profile of l-BHDU. The synthesized prodrugs also cap the polarity of l-BHDU, making it more lipophilic for improved cell membrane transport.

To evaluate these new prodrugs, we tested them in ARPE-19 cells infected with VZV-ORF57-Luc.^34^ VZV-ORF57-Luc expresses firefly luciferase; thus, virus spread was measured by bioluminescence imaging (total flux [photons/s/cm^2^/steradian]) in the IVIS 50 instrument (Caliper Life Sciences/Xenogen, Hopkinton, MA). ARPE-19 cells were seeded in 96-well tissue culture plates 3 d prior to infection and grown to confluence. Cells were infected with cell-free VZV-ORF57-Luc at an MOI of 0.01, as previously described.^16,34−36^ At 2 h post-infection, the virus inoculum was removed, and the medium was replaced with controls or test compounds in 6 replicate wells. CDV or ACV (positive controls), or l-BHDU and its prodrugs, were incubated with infected cells for 72 h. Virus yield was determined by dividing the average total flux at each concentration by the average total flux of the untreated wells.

Cellular toxicity was measured using a neutral red dye uptake assay, as previously described.^16,36,37^ Briefly, ARPE-19 cells were seeded in tissue culture plates for 3 d. Cells were then treated for 72 h with a range of concentrations of the control and experimental compounds. Staurosporine, which causes apoptosis, served as the control for cell death. For both efficacy and cytotoxicity assays, the 50% reduction in efficacy (EC_50_) and cell viability (CC_50_) was determined using GraphPad software (San Diego, California, www.graphpad.com).

**Figure 2:**
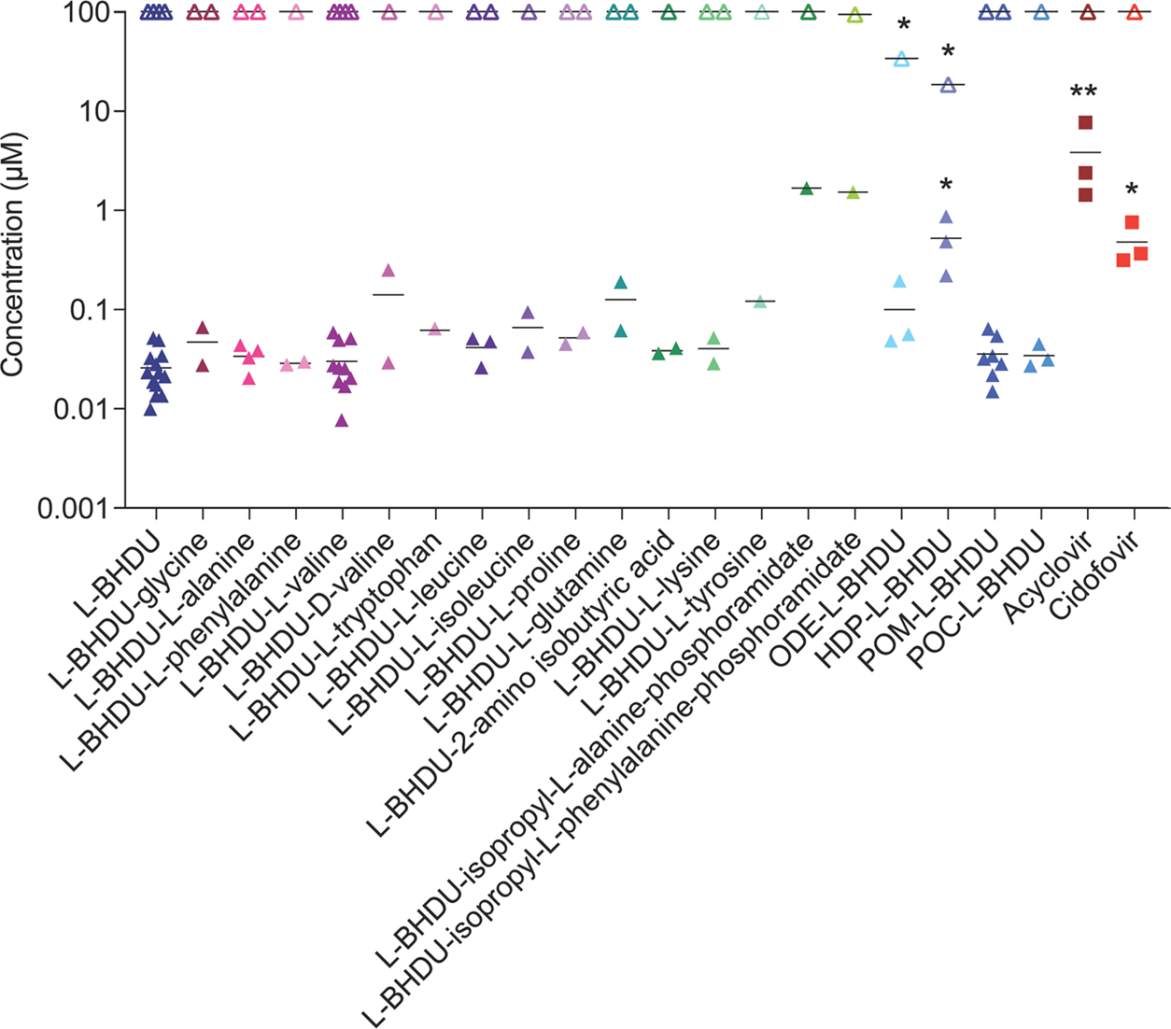
Antiviral activity and cytotoxicity of l-BHDU and its prodrugs against VZV. l-BHDU and its prodrugs were evaluated against VZV-ORF57-Luc in ARPE-19 cells. Closed symbols represent EC_50_ values; open symbols represent CC_50_ values. *N* = 1–13 individual experiments. Each symbol represents the average of 6 replicates per experiment. Asterisks indicate significance [*p < 0.05,**p < 0.01, one-way analysis of variance (ANOVA) with Dunnett’s *post hoc* test].

In this study, l-BHDU prevented VZV-ORF57-Luc spread in APRE-19 cells better than when it was evaluated against another reporter virus, VZV-BAC-Luc, in human foreskin fibroblasts (HFFs) (Figure 2).^16^ This suggests that the virus strain and/or cell type may slightly alter the effects of l-BHDU. Figure 2 shows the EC_50_ and CC_50_ values of synthesized prodrugs; all results are summarized in Table 1.

**Table 1:** Antiviral Activity of l-BHDU and its Prodrugs against Cell-Free VZV in ARPE-19 Cells

s.no.	compounds	EC_50_ (μM)^a^	CC_50_ (μM)^b^	SI (CC_50_/EC_50_)^c^
**1.**	l-BHDU (**1**)	0.0258	>100	>3878
**2.**	l-BHDU-glycine (**14**)	0.0469	>100	>2132
**3.**	l-BHDU-l-alanine (**15**)	0.0338	>100	>2959
**4.**	l-BHDU-l-phenylalanine (**16**)	0.0287	>100	>3484
**5.**	l-BHDU-l-valine (**17**)	0.0301	>100	>3322
**6.**	l-BHDU-d-valine (**18**)	0.141	>100	>709
**7.**	l-BHDU-l-tryptophan (**19**)	0.0645	>100	>1550
**8.**	l-BHDU-l-leucine (**20**)	0.0414	>100	>2415
**9.**	l-BHDU-l-isoleucine (**21**)	0.0658	>100	>1520
**10.**	l-BHDU-l-proline (**22**)	0.0517	>100	>1934
**11.**	l-BHDU-l-glutamine (**23**)	0.126	>100	>794
**12.**	l-BHDU-2-aminoisobutyric acid (**24**)	0.0385	>100	>2597
**13.**	l-BHDU-l-lysine (**25**)	0.0403	>100	>2481
**14.**	l-BHDU-l-tyrosine (**26**)	0.121	>100	>826
**15.**	l-BHDU-isopropyl-l-alanine-phosphoramidate (**33**)	1.689	>100	>206
**16.**	l-BHDU-isopropyl-l-phenylalanine-phosphoramidate (**34**)	1.530	93.72	61
**17.**	ODE-l-BHDU-MP (**38**)	0.100	33.93	339
**18.**	HDP-l-BHDU-MP (**39**)	0.526	18.47	35
**19.**	POM-l-BHDU-MP (**41**)	0.0356	>100	>2809
**20.**	POC-l-BHDU-MP (**47**)	0.0343	>100	>2915
**21.**	acyclovir (ACV)	3.866	>100	>26
**22.**	cidofovir (CDV)	0.482	>100	>208

a 50% inhibitory concentration 72 hpi determined by bioluminescence imaging, mean from at least three experiments.

b 50% cytotoxic concentration at 72 h determined by neutral red assay from 3 experiments.

c Selectivity index = CC_50_/EC_50_.

Most l-BHDU prodrugs had good anti-VZV activity without cytotoxicity, similar to the parent molecule. Among the synthesized amino acid ester prodrugs (**14**–**26**), l-phenylalanine (**16**) and l-valine (**17**) prodrugs were not cytotoxic and had the lowest EC_50_ values at 0.028 μM (SI > 3484) and 0.030 μM (SI > 3322), respectively. Interestingly, we observed a significant drop in antiviral activity when the d-valine configuration was used for the prodrug (**18**, EC_50_ = 0.14 μM). Based on this result, we did not synthesize additional d-amino acid analogues of l-BHDU. The other l-amino acid prodrugs also exhibited moderate to good anti-VZV activity. There was no cytotoxicity associated with l-BHDU-amino acid ester prodrugs (CC_50_ > 100 μM). Comparably, phosphoramidate prodrugs (**33**, **34**) had less antiviral activity. l-BHDU-isopropyl-l-alanine-phosphoramidate (**33**) had a weak antiviral activity with an EC_50_ of 1.689 μM but was not cytotoxic (CC_50_ > 100 μM). l-BHDU-isopropyl-l-phenylalanine-phosphoramidate (**34**) had a weak antiviral activity with an EC_50_ of 1.53 μM and was mildly cytotoxic (CC_50_ = 93.72 μM).

We also evaluated the long-chain lipid phosphate prodrugs octadecyloxyethyl-l-BHDU-MP (ODE-l-BHDU-MP, **38**) and hexadecyloxypropyl-l-BHDU-MP (HDP-l-BHDU-MP, **39**). ODE-l-BHDU-MP (**38**) had moderate anti-VZV activity with an EC_50_ of 0.10 μM, but was also moderately cytotoxic with a CC_50_ of 33.93 μM. HDP-l-BHDU-MP (**39**) had reduced antiviral activity with an EC_50_ of 0.53 μM, and was cytotoxic (CC_50_ = 18.47 μM). We believe that the slower metabolic rate of the long hydrocarbon in the cell causes higher cytotoxicity.

Finally, we tested the phosphate ester prodrugs, POM-l-BHDU-MP (**41**) and POC-l-BHDU-MP (**47**), against VZV in ARPE-19 cells. Both POM-l-BHDU-MP and POC-l-BHDU-MP had good antiviral efficacy and were not cytotoxic. POM-l-BHDU-MP and POC-l-BHDU-MP had EC_50_ values of 0.036 μM (SI > 2809) and 0.034 μM (SI > 2915), respectively, similar to its parent compound, l-BHDU. Other benefits of the long-chain phospholipid and phosphonate ester prodrugs of l-BHDU-MP are that these prodrugs resemble lysophosphatidylcholine^20^ and enhance cellular uptake.

Since l-BHDU (**1**), ODE-l-BHDU-MP (**38**), POM-l-BHDU-MP (**41**), and POC-l-BHDU-MP (**47**) had some of the best antiviral profiles against VZV-ORF57-Luc, we then screened them against VZV TK and TS (thymidylate synthase) mutants.^34^ These studies were performed in the same manner as the efficacy studies described above, except cell-associated VZV-ORF57-Luc, VZV-ORF57-ΔTK, VZV-ORF57-ΔTS, and VZV-ORF57-ΔTKTS were used to infect ARPE-19 cells. As before, CDV and ACV were used as positive controls.

**Figure 3:**
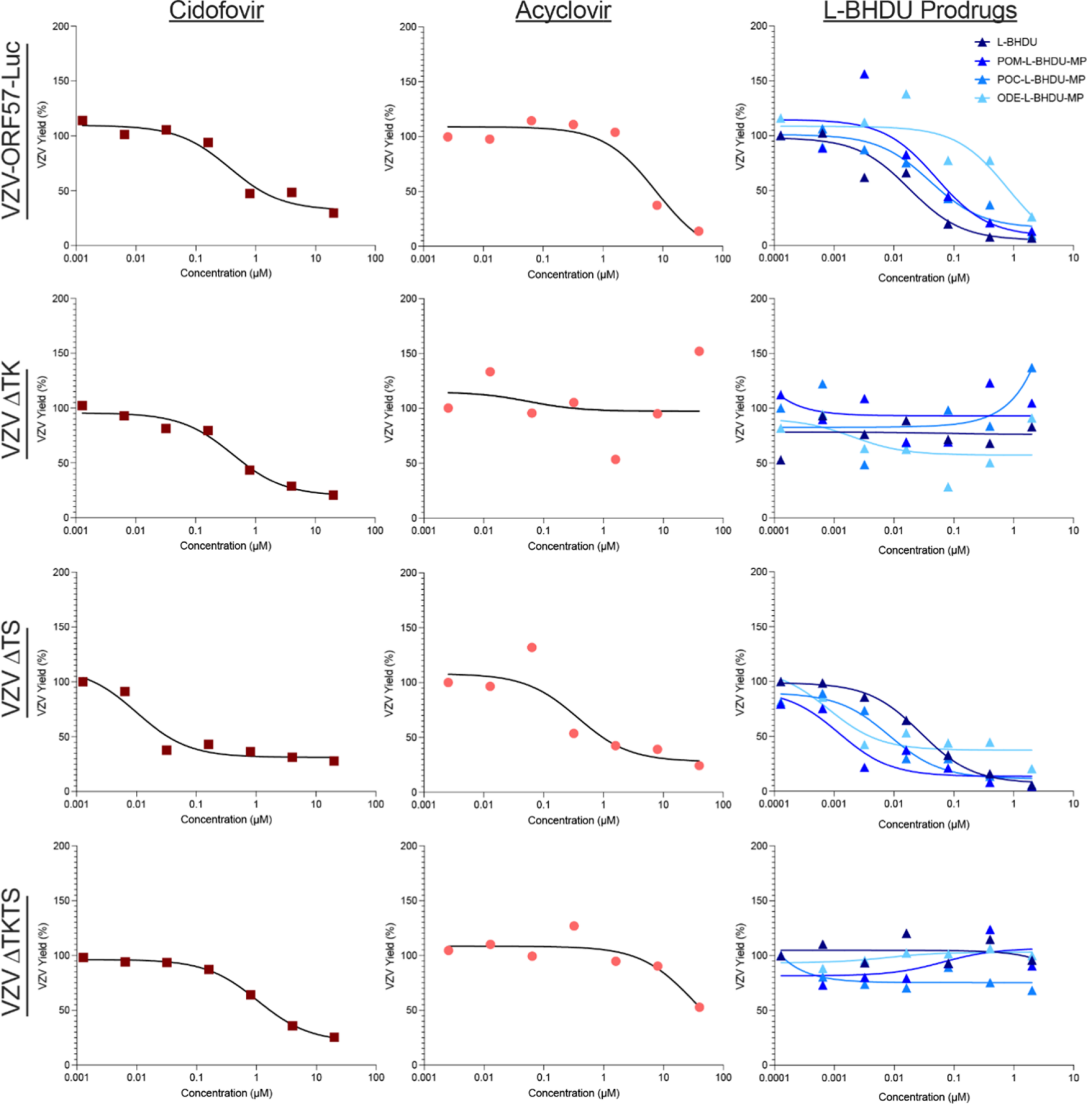
Antiviral screening results for l-BHDU and its C18 (ODE-l-BHDU-MP, **38**) POM (**41**), and POC-l-BHDU-MP (**47**) against cell-associated VZV-ORF57-Luc, VZV TK-, VZV TS-, and VZV TKTS-. Cidofovir and acyclovir are positive controls. Each symbol represents the average of 6 replicate wells; the line is the best-fit curve (error bars are omitted for clarity).

The EC_50_ for each compound against cell-associated VZV was similar to or a little higher than those obtained with cell-free VZV (Table 2). In most cases, l-BHDU and its prodrugs were more potent against VZV-ORF57-Luc than CDV or ACV (Table 2, Figure 3). The exception is ODE-l-BHDU-MP (**38**), which was less potent compared to CDV but 9-fold more potent compared to ACV. As expected, CDV was effective against VZV-ORF57-ΔTK and VZV-ORF57-ΔTKTS, while ACV was not due to its requirement for phosphorylation by viral TK. Unfortunately, l-BHDU and its prodrugs were not active against VZV-ORF57-ΔTK or VZV-ORF57-ΔTKTS.

**Table 2:** Antiviral Activity of l-BHDU and Several Prodrugs against Cell-Associated Wild-Type and Mutant VZV Viruses in ARPE-19 Cells

	EC_50_ (μM)					
viral strains	CDV	ACV	l-BHDU	POM-l-BHDU-MP (**41**)	POC-l-BHDU-MP (**47**)	ODE-l-BHDU-MP (**38**)
VZV-ORF57-Luc	0.37	7.75	0.18	0.051	0.040	0.82
VZV-ORF57-ΔTK	0.42	>20	>2.0	>2.0	>2.0	>2.0
VZV-ORF57-ΔTS	0.01	0.37	0.028	0.0012	0.0084	0.0008
VZV-ORF57-ΔTKTS	1.05	>20	>2.0	>2.0	>2.0	>2.0

Interestingly, l-BHDU and the prodrugs POM-l-BHDU-MP, POC-l-BHDU-MP, and ODE-l-BHDU-MP were more potent against the VZV-ORF57-ΔTS mutant virus than the wild type. This phenomenon was also observed for CDV and ACV. We previously noted this phenomenon with other antivirals against VZV-ORF57-ΔTS, including the thymidine analogue brivudine (BVDU).^34^ We suspect that the loss of TS reduces the dTMP pools in the cell, resulting in less competition with l-BHDU or its prodrugs for incorporation during viral DNA synthesis. Thus, while these prodrugs do not work against VZV TK mutants, l-BHDU and its prodrugs are still highly effective at reducing VZV spread and should be investigated further. In the event of viral thymidine kinase (TK) mutation, the nucleoside class of antivirals does not proceed to the first monophosphorylation step.

The unphosphorylated nucleoside remains in its inactive form and does not interfere with viral replication. Therefore, it was thought to select the prodrugs that bypass the viral TK-driven monophosphorylation or do not require the often rate-limiting initial phosphorylation step for activation. Considering these points, POM-l-BHDU-MP and ODE-l-BHDU-MP were selected for the elaborated *in vivo* studies *via* subcutaneous and oral routes. Studies to evaluate these prodrugs in human skin organ culture and human skin xenografts in athymic nude mice (NuSkin mouse model) are forthcoming.

### Pharmacokinetic Studies

**Figure 4:**
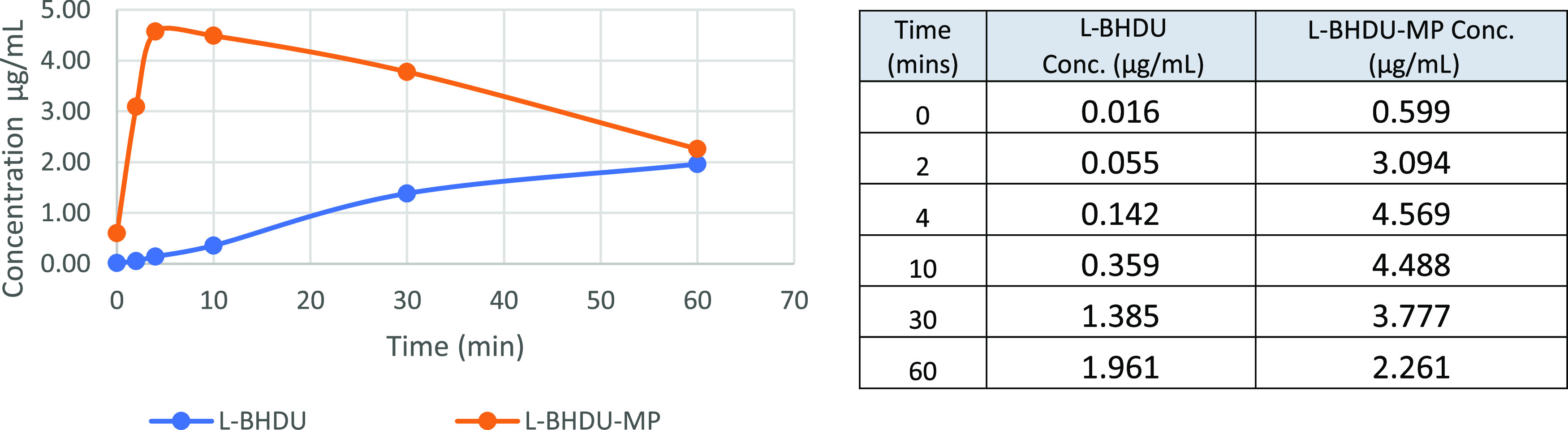
*In vitro* stability study of POM-l-BHUD-MP in mouse liver homogenates.

The antiviral efficacy of POM-l-BHDU-MP prompted us to perform pharmacokinetic studies on POM-l-BHDU-MP *in vitro* and *in vivo*. POM-l-BHDU-MP demonstrated good stability in gastric and intestinal juice (data not shown). In liver homogenates, POM-l-BHDU-MP converted to l-BHDU and l-BHDU-MP (Figure 4). POM-l-BHDU-MP incubated with homogenized mouse liver was unstable and converted to l-BHDU and l-BHDU-MP. The concentration of l-BHDU increased steadily, reaching 1.9 μg/mL after 60 min. l-BHDU-MP appeared rapidly and reached maximal concentration (4.6 μg/mL) after 4 min, which is 32-fold higher than the corresponding l-BHDU concentration. Thus, POM-l-BHDU-MP likely converts rapidly into l-BHDU-MP and more slowly into l-BHDU in the liver.

A pharmacokinetic absorption study was performed in BALB/c mice (Charles River). A single equimolar dose of 22.5 mg/kg POM-l-BHDU-MP or 11.4 mg/kg l-BHDU was dissolved in Cremophor–dimethyl sulfoxide (DMSO)–saline (1:1:8) and administered by oral gavage to two groups of mice with equal numbers of male and female mice (*N* = 15 each male and female).

**Figure 5:**
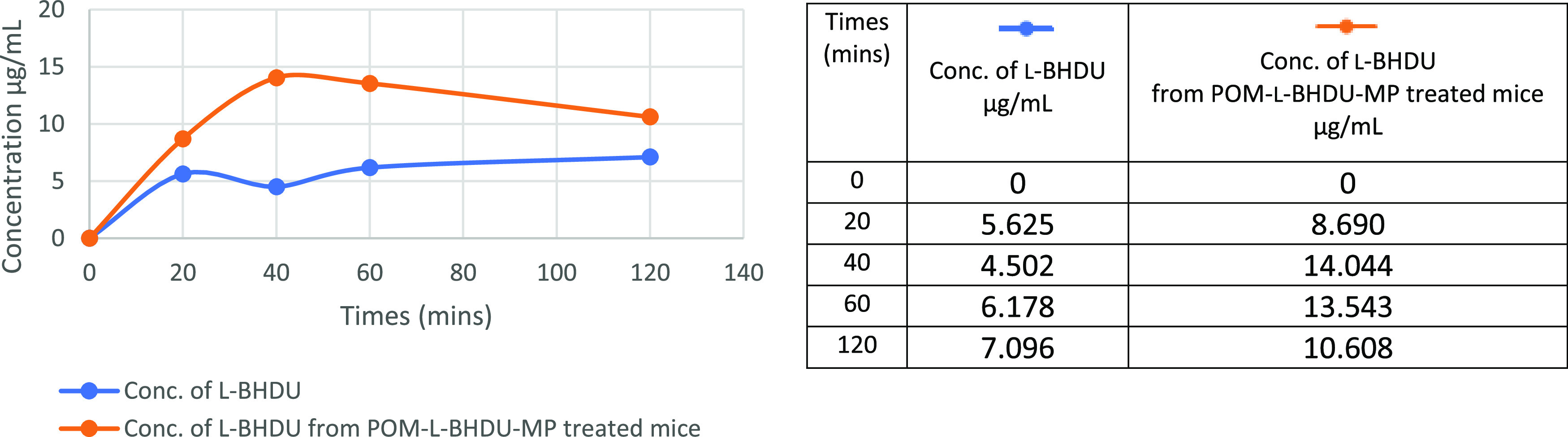
l-BHDU plasma concentration–time profiles after oral administration of l-BHDU or POM-l-BHDU-MP.

Mouse blood was collected by cardiac puncture at the times presented in Figure 5 (see table). Plasma was separated from red blood cells and immediately frozen at −80 °C. l-BHDU absorption profiles are shown in Figure 5. The results indicate that l-BHDU was significantly more bioavailable when administered as the prodrug form of POM-l-BHDU-MP.

The AUC value for l-BHDU in POM-l-BHDU-MP treated mice is approximately 2.2-fold higher than for l-BHDU treated mice (Table 3). In mice given POM-l-BHDU-MP, the maximal plasma concentration of l-BHDU was 14 μg/mL at 40 min (Figure 5), whereas a lower l-BHDU plasma concentration, 4.5 to 7.0 μg/mL, was observed in mice given l-BHDU. *C*_max_ for l-BHDU was significantly higher after administration of prodrug POM-l-BHDU-MP than the parent molecule (l-BHDU, Table 3). Thus, the POM prodrug had better oral absorption/availability than l-BHDU. We found that in mice administered POM-l-BHDU-MP, only the l-BHDU metabolite was detected *via* liquid chromatography/mass spectrometry (LC/MS) in plasma, and the trace amount of l-BHDU-MP detected was below the limit of quantitation. This suggests that POM-l-BHDU-MP is absorbed and rapidly cleaved by esterases at the (P–O) bond of l-BHDU-MP to release the parental nucleoside, l-BHDU.

**Table 3:** Pharmacokinetic Parameters after Oral Application of 22.5 mg/kg POM-l-BHDU-MP and 11.4 mg/kg l-BHDU to BALB/c Mice.^a^

	l-BHDU derived from POM-l-BHDU-MP	l-BHDU
AUC (μg·min/mL)	1680	764
*T*_max_ (min)	40	120
*C*_max_ (μg/mL)	10.6	7.1

a LC-MS quantification of l-BHDU from POM-l-BHDU-MP- and l-BHDU-treated mice. AUC: area under the curve from 0 to 120 min; *T*_max_: time taken to reach maximum conc.; and *C*_max_: maximum concentration.

## Conclusions

Here, we described the synthesis and antiviral evaluation of l-BHDU and its prodrugs, including 5′-amino acid esters, phosphoramidates, long-chain phospholipids, and phosphate esters. *In vitro*, most of these prodrugs exhibited significant anti-VZV activity. Of particular interest were POM-l-BHDU-MP, POC-l-BHDU-MP, and ODE-l-BHDU-MP. POM-l-BHDU-MP (**41**) and POC-l-BHDU-MP (**42**) had similar antiviral activity compared to l-BHDU, whereas ODE-l-BHDU-MP (**38**) was less active than the parental molecule. The pharmacokinetic study revealed that POM-l-BHDU-MP prodrugs have 2.2 times better oral absorption/availability in comparison to l-BHDU. Based on these data, we conclude that ODE-l-BHDU-MP (**38**), POM-l-BHDU-MP (**41**), and POC-l-BHDU-MP (**42**) have potent antiviral profiles and warrant continued development as novel antiviral treatments for VZV infections. Additional studies are planned to evaluate these compounds in more clinically relevant systems, including a human skin organ culture system and in the NuSkin mouse model,^38^ as well as the elaborated pharmacokinetics of the prodrugs to select them as potential clinical candidates.

## Experimental Section

### General Analytical Methods

Reagents and anhydrous solvents were purchased from commercial sources and used without further purification. Moisture-sensitive reactions were performed using oven-dried glassware under a nitrogen or argon atmosphere. Reactions were monitored by thin-layer chromatography plates (TLC silica gel GF 250 μm) that were visualized using a Spectroline UV lamp (254 nm) and developed with a 15% solution of sulfuric acid in methanol. Column chromatography was performed on silica gel 60 Å, 40–63 μM (230 × 400 mesh, Sorbent Technologies). Preparative normal phase chromatography was performed on a CombiFlash Rf 150 (Teledyne Isco) with prepacked RediSep Rf silica gel cartridges or on RediSep gold C18 reverse phase columns. Melting points were recorded on a Mel-temp II laboratory device and were uncorrected. Nuclear magnetic spectra were recorded on a Varian Inova 500 spectrometer at 500 MHz for ^1^H NMR, 202 MHz for ^31^P NMR, and 125 MHz for ^13^C NMR with tetramethylsilane as an internal standard. Chemical shifts (δ) are quoted as s (singlet), bs (broad singlet), d (doublet), t (triplet), q (quartet), m (multiplet), dd (double doublet), and dt (double triplet). Optical rotations were measured on a JASCO DIP-370 digital polarimeter. High-resolution mass spectroscopy (HRMS) spectra were measured on a Bruker Ultrahigh-resolution QTOF MS Impact II spectrometer. Samples were infused at 3 μL/min, and spectra were obtained in the positive or negative ionization mode with a typical resolution of 20,000 or greater. The purity of all tested compounds is ≥95%, as determined by their elemental analysis (Table S1) or by high-performance liquid chromatography/ultraviolet (HPLC/UV). Elemental analyses were performed by Atlantic Microlab Inc. Norcross, GA. HPLC/UV was determined with a Waters HPLC coupled to a photodiode array. Five microliters of sample 0.5 mg/mL in methanol, acetonitrile, or a mixture of DMSO/MeOH (0.5:10) was injected using an XBridge C18, 3.5 μm (4.6 × 150 mm) column at 25 °C with a flow rate of 0.8 mL/min or with UPLC BEH C18, 1.7 μm (100 × 2.1) mm at 50 °C with a flow rate of 0.55 mL/min. The mobile phases were a mixture of *A* = 10 mM ammonium acetate in water and *B* = acetonitrile (ACN), and *A* = 0.05% formic acid (FA) in water and *B* = 0.05% in acetonitrile (ACN). Purity is given as % of absorbance at the Max plot.

#### General Procedure for the Synthesis of Boc-*N-*protected-5′-amino Acid Ester of l-BHDU (**2**–**13**)

A solution of l-BHDU (1 mmol), Boc-protected amino acid (2.5 mmol), DMAP (2.5 mmol), and 1,3-diisopropylcarbodiimide (DIC, 2.5 mmol) in DCM (10 mL) was stirred at rt from 12 h. The mixture was diluted with DCM (10 mL) and washed with aqueous sodium bicarbonate (2 × 10 mL) and finally with water (2 × 10 mL). The organic layer was dried over Na_2_SO_4_, filtered, and concentrated under reduced pressure. The residue was purified by column chromatography to provide 5′-*O*-Boc-protected amino acid ester of l-BHDU in 60–85% yield.

#### l-BHDU-5′-*O*-l-Boc-glycine Ester (**2**)

Yield: 78%; ^1^H NMR (500 MHz, CDCl_3_) δ 9.59 (bs, 1H), 7.54 (s, 1H), 7.41 (d, *J* = 15.0 Hz, 1H), 6.73 (d, *J* = 13.6 Hz, 1H), 6.31 (d, *J* = 4.4 Hz, 1H), 5.18 (t, *J* = 3.2 Hz, 1H), 5.11 (s, 1H), 4.51 (dd, *J* = 12.4 & 3.3 Hz, 1H), 4.38 (dd, *J* = 12.4 & 3.0 Hz, 1H), 4.27 (d, *J* = 10.1 Hz, 1H), 4.19–4.16 (m, 1H), 3.98–3.88 (m, 2H), 1.44 (s, 9H); ^13^C NMR (125 MHz, CDCl_3_) δ 170.1, 161.4, 155.8, 149.6, 136.9, 128.1, 112.2, 110.9, 102.9, 81.5, 80.4, 71.3, 62.8, 42.3, 28.4; HRMS (EI) calcd for (C_17_H_22_BrN_3_O_8_ + H)^+^ 476.0669, found 476.0663.

#### l-BHDU-5′-*O*-l-Boc-alanine Ester (**3**)

Yield: 71%; ^1^H NMR (500 MHz, CDCl_3_) δ 9.53 (bs, 1H), 7.58 (s, 1H), 7.44 (d, *J* = 13.6 Hz, 1H), 6.76 (d, *J* = 13.6 Hz, 1H), 6.34 (dd, *J* = 5.7 & 1.8 Hz, 1H), 5.18 (t, *J* = 3.3 Hz, 1H), 5.03 (d, *J* = 6.4 Hz, 1H), 4.57 (d, *J* = 10.2 Hz, 1H), 4.39–4.23 (m, 3H), 4.18 (dd, *J* = 10.4 & 5.7 Hz, 1H), 1.43 (s, 9H), 1.38 (d, *J* = 7.3 Hz, 3H); ^13^C NMR (125 MHz, CDCl_3_) δ 173.2, 161.2, 155.2, 150.0, 137.0, 128.1, 112.3, 110.9, 102.9, 81.4, 71.8, 62.8, 49.3, 42.3, 28.3, 23.5, 18.1; HRMS (EI) calcd for (C_18_H_24_BrN_3_O_8_ – H)^−^ 488.0669, found 488.0670.

#### l-BHDU-5′-*O*-l-Boc-phenylalanine Ester (**4**)

Yield: 82%; ^1^H NMR (500 MHz, CDCl_3_) δ 9.69 (bs, 1H), 7.54 (s, 1H), 7.47 (d, *J* = 13.6 Hz, 1H), 7.32–7.25 (m, 3H), 7.13 (d, *J* = 13.6 Hz, 2H), 6.77 (d, *J* = 13.6 Hz, 1H), 6.33 (dd, *J* = 5.7 & 1.7 Hz, 1H), 5.12 (t, *J* = 3.4 Hz, 1H), 5.02 (d, *J* = 7.7 Hz, 1H), 4.58–4.52 (m, 2H), 4.28–4.23 (m, 2H), 4.20–4.14 (m, 1H), 3.13–3.0 (m, 1H), 3.03 (dd, *J* = 13.8 & 6.9 Hz, 1H), 1.41 (s, 9H); ^13^C NMR (125 MHz, CDCl_3_) δ 171.9, 161.5, 152.2, 149.7, 137.0, 135.6, 129.2, 128.8, 128.2, 127.3, 112.3, 111.0, 102.9, 81.4, 80.3, 71.1, 62.9, 54.6, 53.5, 38.1, 28.3; HRMS (EI) calcd for (C_24_H_28_BrN_3_O_8_ – H)^−^ 564.0982, found 564.0985.

#### l-BHDU-5′-*O*-l-Boc-valine Ester (**5**)

Yield: 83%; ^1^H NMR (500 MHz, CDCl_3_) δ 8.84 (bs, 1H), 7.60 (s, 1H), 7.48 (d, *J* = 13.6 Hz, 1H), 6.78 (d, *J* = 13.6 Hz, 1H), 6.33 (d, *J* = 7.1 Hz, 1H), 5.19 (t, *J* = 3.2 Hz, 1H), 4.96 (d, *J* = 8.8 Hz, 1H), 4.61 (dd, *J* = 12.3 & 4.0 Hz, 1H), 4.30–4.23 (m, 2H), 4.20–4.13 (m, 2H), 2.14–2.05 (m, 1H), 1.43 (s, 9H), 0.97 (d, *J* = 6.8 Hz, 3H), 0.90 (d, *J* = 6.8 Hz, 3H).^13^C NMR (125 MHz, CDCl_3_) δ 172.3, 161.1, 155.8, 149.5, 137.1, 128.2, 112.3, 110.0, 103.0, 81.5, 80.2, 71.2, 62.6, 58.9, 30.9, 28.4, 19.2, 17.8; HRMS (EI) calcd for (C_20_H_28_BrN_3_O_8_ + H)^+^ 518.1138, found 518.1132.

#### l-BHDU-5′-*O*-d-Boc-valine Ester (**6**)

Yield: 73%; ^1^H NMR (500 MHz, CDCl_3_) δ 9.39 (bs, 1H), 7.56 (s, 1H), 7.46 (d, *J* = 13.6 Hz, 1H), 6.75 (d, *J* = 13.6 Hz, 1H), 6.28 (d, *J* = 7.1 Hz, 1H), 5.19 (t, *J* = 3.7 Hz, 1H), 4.99 (d, *J* = 8.7 Hz, 1H), 4.51 (dd, *J* = 12.3 & 4.0 Hz, 1H), 4.37 (dd, *J* = 12.3 & 3.2 Hz, 1H), 4.25 (dd, *J* = 10.4 & 1.5 Hz, 1H), 4.22–4.15 (m, 2H), 2.15–2.08 (m, 1H), 1.43 (s, 9H), 0.97 (d, *J* = 6.8 Hz, 3H), 0.90 (d, *J* = 6.8 Hz, 3H); ^13^C NMR (125 MHz, CDCl_3_) δ 172.2, 161.2, 155.7, 149.4, 136.7, 128.1, 112.1, 111.0, 103.1, 81.9, 80.2, 71.7, 62.9, 58.8, 31.0, 28.4, 19.2, 17.7; HRMS (EI) calcd for (C_20_H_28_BrN_3_O_8_ + H)^+^ 518.1138, found 518.1135.

#### l-BHDU-5′-*O*-l-Boc-tryptophan Ester (**7**)

Yield: 71%; ^1^H NMR (500 MHz, CDCl_3_) δ 9.42 (bs, 1H), 8.34 (bs, 1H), 7.52 (d, *J* = 7.9 Hz, 1H), 7.49–7.38 (m, 2H), 7.33 (d, *J* = 8.1 Hz, 1H), 7.17 (t, *J* = 7.4 Hz, 1H), 7.09 (t, *J* = 7.2 Hz, 1H), 7.03 (s, 1H), 6.73 (d, *J* = 13.6 Hz, 1H), 6.23 (d, *J* = 5.6 Hz, 1H), 5.14 (d, *J* = 7.8 Hz, 1H), 4.96 (s, 1H), 4.61 (d, *J* = 6.9 Hz, 1H), 4.44 (d, *J* = 9.5 Hz, 1H), 4.22–4.14 (m, 2H), 4.04 (dd, *J* = 10.4 & 5.7 Hz, 1H), 3.24 (d, *J* = 6.0 Hz, 2H), 1.43 (s, 9H); ^13^C NMR (125 MHz, CDCl_3_) δ 172.3, 161.3, 155.3, 149.6, 137.0, 136.2, 128.2, 127.6, 123.0, 122.2, 119.7, 118.6, 112.3, 111.4, 111.0, 109.6, 103.0, 81.3, 80.3, 71.0, 62.8, 54.3, 53.5, 28.4; HRMS (EI) calcd for (C_26_H_29_BrN_4_O_8_ – H)^−^ 603.1091, found 603.1096.

#### l-BHDU-5′-*O*-l-Boc-leucine Ester (**8**)

Yield: 67%; ^1^H NMR (500 MHz, CDCl_3_) δ 9.48 (bs, 1H), 7.61 (s, 1H), 7.45 (d, *J* = 13.6 Hz, 1H), 6.78 (d, *J* = 13.6 Hz, 1H), 6.33 (dd, *J* = 5.7 & 1.9 Hz, 1H), 5.18 (t, *J* = 3.0 Hz, 1H), 4.95 (d, *J* = 8.1 Hz, 1H), 4.61 (d, *J* = 12.2 Hz, 1H), 4.28–4.21 (m, 3H), 4.17 (dd, *J* = 10.3 & 5.8 Hz, 1H), 1.74–1.69 (m, 1H), 1.60–1.48 (m, 2H), 1.41 (s, 9H), 0.92 (dd, *J* = 6.6 & 3.6 Hz, 6H); ^13^C NMR (125 MHz, CDCl_3_) δ 173.4, 161.5, 155.5, 150.0, 137.2, 128.3, 112.3, 111.0, 103.0, 81.5, 80.2, 71.2, 63.0, 52.4, 50.8, 41.1, 28.4, 25.0, 23.5, 23.0, 21.9; HRMS (EI) calcd for (C_21_H_30_BrN_3_O_8_ – H)^−^ 530.1138, found 530.1141.

#### l-BHDU-5′-*O*-l-Boc-isoleucine Ester (**9**)

Yield: 72%; ^1^H NMR (500 MHz, CDCl_3_) δ 9.62 (bs, 1H), 7.58 (s, 1H), 7.46 (d, *J* = 13.8 Hz, 1H), 6.77 (d, *J* = 13.6 Hz, 1H), 6.33 (dd, *J* = 5.7 & 1.7 Hz, 1H), 5.18 (t, *J* = 3.4 Hz, 1H), 5.02 (d, *J* = 8.7 Hz, 1H), 4.59 (dd, *J* = 12.3 & 2.9 Hz, 1H), 4.26 (d, *J* = 11.3 Hz, 2H), 4.21–4.15 (m, 2H), 1.85–1.77 (m, 1H), 1.42 (s, 10H), 1.20–1.12 (m, 1H), 0.93–0.87 (m, 6H); ^13^C NMR (125 MHz, CDCl_3_) δ 172.3, 161.5, 155.7, 149.7, 137.0, 128.2, 112.3, 111.0, 103.0, 81.5, 80.2, 71.1, 62.6, 58.3, 37.6, 28.5, 25.0, 15.7, 11.5; HRMS (EI) calcd for (C_21_H_30_BrN_3_O_8_ + H)^+^ 532.1295, found 532.1284.

#### l-BHDU-5′-*O*-l-Boc-proline Ester (**10**)

Yield: 75%; ^1^H NMR (500 MHz, CDCl_3_) δ 9.80 (bs, 1H), 7.7–7.58 (m, 1H), 7.43 (d, *J* = 11.0 Hz, 1H), 6.74 (dd, *J* = 28.4 & 13.6 Hz, 1H), 6.36 (t, *J* = 4.0 Hz, 1H), 5.19–5.15 (m, 1H), 4.57 (ddd, *J* = 38.3, 12.5 & 3.0 Hz, 1H), 4.30–4.14 (m, 4H), 3.59–3.36 (m, 2H), 2.26–2.11 (m 1H), 1.98–1.94 (m, 3H), 1.43–1.40 (m, 9H); ^13^C NMR (125 MHz, CDCl_3_) δ 172.7, 161.5, 154.5, 149.9, 137.7, 128.5, 112.4, 110.9, 103.0, 81.4, 80.2, 71.4, 62.4, 58.9, 53.6, 46.6, 29.9, 23.7; HRMS (EI) calcd for (C_20_H_26_BrN_3_O_8_ – H)^−^ 514.0825, found 514.0826.

#### l-BHDU-5′-*O*-l-Boc-glutamine Boc Amide Ester (**11**)

Yield: 79%; ^1^H NMR (500 MHz, CDCl_3_) δ 9.99 (s, 1H), 7.57 (s, 1H), 7.44 (d, *J* = 13.6 Hz, 1H), 6.78 (d, *J* = 13.6 Hz, 1H), 6.33 (d, *J* = 5.6 Hz, 1H), 6.08 (d, *J* = 36.8 Hz, 2H), 5.48 (d, *J* = 7.6 Hz, 1H), 5.20 (t, *J* = 3.1 Hz, 1H), 4.52 (d, *J* = 11.9 Hz, 1H), 4.36–4.15 (m, 4H), 2.33 (t, *J* = 7.1 Hz, 2H), 2.14–2.0 (m, 1H), 1.98–1.87 (m, 1H), 1.43 (s, 9H); ^13^C NMR (125 MHz, CDCl_3_) δ 175.1, 172.2, 161.9, 155.8, 150.0, 137.5, 128.5, 112.2, 110.5, 102.7, 81.3, 80.3, 70.8, 63.0, 53.4, 31.8, 28.4; HRMS (EI) calcd for (C_20_H_27_BrN_4_O_9_+Na)^+^ 569.0859, found 569.0851.

#### l-BHDU-5′-*O*-Boc-2-aminoisobutyric Ester (**12**)

Yield: 66%; ^1^H NMR (500 MHz, CDCl_3_) δ 9.57 (bs, 1H), 7.52 (s, 1H), 7.44 (d, *J* = 13.6 Hz, 1H), 6.73 (d, *J* = 13.6 Hz, 1H), 6.30 (d, *J* = 5.3 Hz, 1H), 5.17 (t, *J* = 3.8 Hz, 1H), 5.02 (bs, 1H), 4.46 (dd, *J* = 12.1 & 3.7 Hz, 1H), 4.33 (dd, *J* = 12.2 & 3.6 Hz, 1H), 4.23 (d, *J* = 10.4 Hz, 1H), 4.16 (dd, *J* = 10.4 & 5.6 Hz, 1H), 1.49 (s, 6H), 1.41 (s, 9H); ^13^C NMR (125 MHz, CDCl_3_) δ 174.2, 161.4, 154.6, 149.6, 136.9, 128.2, 112.3, 110.9, 103.2, 81.5, 71.4, 63.4, 56.1, 28.3, 25.6; HRMS (EI) calcd for (C_19_H_26_BrN_3_O_8_ – H)^−^ 502.0825, found 502.0830.

#### l-BHDU-5′-*O*-l-Boc-lysine Amide Ester (**13**)

Yield: 85%; ^1^H NMR (500 MHz, CDCl_3_) δ 9.67 (bs, 1H), 7.58 (s, 1H), 7.48–7.41 (m, 1H), 6.77 (d, *J* = 13.8 Hz, 1H), 6.33 (d, *J* = 5.4 Hz, 1H), 5.17 (t, *J* = 3.3 Hz, 2H), 4.67 (s, 1H), 4.56 (d, *J* = 12.4 Hz, 1H), 4.27 (d, *J* = 11.2 Hz, 2H), 4.22–4.13 (m, 2H), 3.11–3.04 (m, 2H), 1.80–1.73 (m, 1H), 1.67–1.58 (m, 1H), 1.47–1.40 (m, 22H); ^13^C NMR (125 MHz, CDCl_3_) δ 172.7, 161.4, 156.2, 155.6, 149.7, 137.1, 128.3, 112.3, 110.8, 102.9, 81.4, 80.2, 79.4, 71.0, 62.7, 53.6, 53.5, 39.9, 31.8, 29.7, 28.6, 28.4, 22.6; HRMS (EI) calcd for (C_26_H_39_BrN_4_O_10_ + H)^+^ 647.1928, found 647.1926.

#### General Procedure for the Synthesis of 5′-Amino Acid Ester of l-BHDU (**14–25**)

The Boc-protected amino acid prodrug (150 mg) was dissolved in DCM (10 mL), and the solution was cooled to 0 °C, then TFA (2 mL) was added to the solution with vigorous stirring. The mixture was warmed to rt and stirred for 1 h. The excess volatiles were removed under pressure. The obtained crude was dissolved in methanol (10 mL) and cooled to 0 °C, then 2 M HCl solution was added in ether and continued stirring for 20 min. The solvents were removed under reduced pressure, and the crude solid obtained was further washed with anhydrous ether to give l-BHDU-5′-*O*-amino acid ester as a hydrochloride salt in approximately 80–90% yield.

#### l-BHDU-5′-*O*-glycyl Ester Hydrochloride (**14**)

Yield: 82%; ^1^H NMR (500 MHz, CD_3_OD) δ 7.70 (s, 1H), 7.39 (d, *J* = 15.0 Hz, 1H), 6.86 (d, *J* = 15.0 Hz, 1H), 6.30 (dd, *J* = 5.0 & 2.5 Hz, 1H), 5.24 (t, *J* = 5.0 Hz, 1H), 4.56–4.49 (m, 2H), 4.38 (dd, *J* = 10.0 & 5.0 Hz, 1H), 4.15–4.13 (m, 1H), 3.87–3.77 (m, 2H); ^13^C NMR (125 MHz, CD_3_OD) δ 167.0, 162.1, 150.1, 138.3, 129.0, 111.3, 108.4, 102.4, 81.7, 70.2, 63.5, 39.6; anal. calcd for C_12_H_15_BrClN_3_O_6_·(H_2_O)_1.5_: C, 32.78; H, 4.13; N, 9.56. Found: C, 32.39, H, 3.90; N, 9.23; HRMS (EI) calcd for (C_12_H_14_BrN_3_O_6_ + H)^+^ 376.0144, found 376.0141.

#### l-BHDU-5′-*O*-l-alanyl Ester Hydrochloride (**15**)

Yield: 89%; [α]_D_^24^ = +4.27 (*c* 0.5, MeOH); ^1^H NMR (500 MHz, DMSO-*d*_6_) δ 11.6 (s, 1H, NH), 8.42 (bs, 3H), 7.65 (s, 1H), 7.30 (d, *J* = 15.0 Hz, 1H), 6.99 (d, *J* = 10.0 Hz, 1H), 6.23 (dd, *J* = 5.0 & 2.5 Hz, 1H), 5.16 (t, *J* = 2.5 Hz, 1H), 4.46–4.40 (m, 2H), 4.32 (dd, *J* = 10.0 & 5.0 Hz, 1H), 4.14–4.06 (m, 2H), 1.33 (d, *J* = 10.0 Hz, 3H); ^13^C NMR (125 MHz, DMSO-*d*_6_) δ 169.5, 162.1, 150.0, 138.2, 129.0, 111.4, 108.4, 102.3, 81.7, 70.1, 63.8, 14.8; anal. calcd for C_13_H_17_BrClN_3_O_6_·(H_2_O)_1.0_: C, 35.11; H, 4.31; N, 9.45. Found: C, 35.05, H, 4.32; N, 9.28; HRMS (EI) calcd for (C_13_H_16_BrN_3_O_6_ + H)^+^ 390.0301, found 390.0296.

#### l-BHDU-5′-*O*-l-phenylalanyl Ester Hydrochloride (**16**)

Yield: 85%; [α]_D_^24^ = −11.26 (*c* 0.5, MeOH); ^1^H NMR (500 MHz, CD_3_OD) δ 7.63 (s, 1H), 7.38–7.28 (m, 4H), 7.18 (d, *J* = 8.5 Hz, 2H), 6.82 (d, *J* = 14.0 Hz, 1H), 6.30 (dd, *J* = 5.5 & 1.5 Hz, 1H), 5.17 (t, *J* = 3.5 Hz, 1H), 4.52 (dd, *J* = 12.5 & 3.5 Hz, 1H), 4.44 (dd, *J* = 12.5 & 3.0 Hz, 1H), 4.38 (dd, *J* = 10.5 & 1.5 Hz, 1H), 4.27 (t, *J* = 6.5 Hz, 1H), 4.19–4.15 (m, 1H), 3.21–3.10 (m, 2H); ^13^C NMR (125 MHz, CD_3_OD) δ 168.6, 162.0, 150.0, 138.1, 133.4, 129.1, 127.8, 111.5, 108.5, 102.1, 100.0, 81.6, 69.9, 63.7, 53.8, 36.1; anal. calcd for C_19_H_21_BrClN_3_O_6_·(H_2_O)_1.0_: C, 43.82; H, 4.45; N, 8.07. Found: C, 43.75, H, 4.51; N, 7.98; HRMS (EI) calcd for (C_19_H_20_BrN_3_O_6_ + H)^+^ 466.0614, found 466.0615.

#### l-BHDU-5′-*O*-l-valyl Ester Hydrochloride (**17**)

Yield: 84%; [α]_D_^24^ = −7.4 (*c* 0.5, MeOH); ^1^H NMR (500 MHz, CD_3_OD) δ 7.74 (s, 1H), 7.47 (d, *J* = 13.5 Hz, 1H), 6.91 (d, *J* = 14.0 Hz, 1H), 6.37 (d, *J* = 4.5 Hz, 1H), 5.25 (t, *J* = 3.0 Hz, 1H), 4.63–4.54 (m, 2H), 4.45 (d, *J* = 10.5 Hz, 1H), 4.26–4.23 (m, 1H), 3.95 (d, *J* = 4.5 Hz, 1H), 2.31–2.28 (m, 1H), 1.09 (d, *J* = 7.0 Hz, 3H), 1.05 (d, *J* = 7.0 Hz, 3H); ^13^C NMR (125 MHz, CD_3_OD) δ 168.6, 162.1, 150.1, 138.3, 129.0, 111.4, 108.5, 102.2, 81.8, 70.0, 63.8, 58.1, 29.7, 17.2, 16.6; anal. calcd for C_15_H_21_BrClN_3_O_6_·(H_2_O)_0.5_: C, 38.85; H, 4.78; N, 9.06. Found: C, 38.82, H, 4.85; N, 8.96; HRMS (EI) calcd for (C_15_H_20_BrN_3_O_6_ + H)^+^ 418.0614, found 418.0612.

#### l-BHDU-5′-*O*-d-valyl Ester Hydrochloride (**18**)

Yield: 86%; [α]_D_^24^ = −15.85 (*c* 0.5, MeOH); ^1^H NMR (500 MHz, CD_3_OD) δ 7.70 (s, 1H), 7.39 (dd, *J* = 13.5 & 3.5 Hz, 1H), 6.84 (d, *J* = 13.0 Hz, 1H), 6.27 (dd, *J* = 6.0 & 2.0 Hz, 1H), 5.25 (t, *J* = 3.5 Hz, 1H), 4.60–4.57 (m, 2H), 4.38 (dd, *J* = 5.5 & 1.5 Hz, 1H), 4.20–4.17 (m, 1H), 3.85 (d, *J* = 4.5 Hz, 1H), 2.31–2.24 (m, 1H), 1.04 (d, *J* = 7.0 Hz, 6H); ^13^C NMR (125 MHz, CD_3_OD) δ 168.5, 162.1, 150.0, 138.1, 129.0, 111.2, 108.4, 102.3, 82.0, 70.3, 63.5, 58.0, 29.7, 17.2, 16.5; anal. calcd for C_15_H_21_BrClN_3_O_6_·(H_2_O)_1.2_: C, 37.82; H, 4.95; N, 8.82. Found: C, 37.69, H, 5.12; N, 8.65; HRMS (EI) calcd for (C_15_H_20_BrN_3_O_6_ + H)^+^ 418.0614, found 418.0611.

#### l-BHDU-5′-*O*-l-tryptophan Ester Hydrochloride (**19**)

Yield: 90%; [α]_D_^24^ = −39.12 (*c* 0.5, MeOH); ^1^H NMR (500 MHz, CD_3_OD) δ 7.69 (s, 1H), 7.51 (d, *J* = 7.8 Hz, 1H), 7.37–7.32 (m, 2H), 7.12–7.07 (m, 2H), 7.01 (t, *J* = 7.5 Hz, 1H), 6.84 (d, *J* = 13.6 Hz, 1H), 6.29 (d, *J* = 4.2 Hz, 1H), 5.11 (t, *J* = 3.0 Hz, 1H), 4.45 (dd, *J* = 12.5 & 3.1 Hz, 1H), 4.35 (d, *J* = 11.9 Hz, 1H), 4.25 (dd, *J* = 12.5 & 3.0 Hz, 1H), 4.16 (dd, *J* = 10.4 & 5.8 Hz, 1H), 3.81 (t, *J* = 6.0 Hz, 1H), 3.17 (dd, *J* = 6.0, 2.8 Hz, 2H); ^13^C NMR (125 MHz, CD_3_OD) δ 168.8, 162.0, 150.0, 138.0, 136.9, 128.9, 126.9, 124.3, 121.7, 119.1, 117.4, 111.3, 108.5, 105.6, 102.1, 81.6, 70.1, 63.9, 53.3, 26.1; anal. calcd for C_15_H_21_BrClN_3_O_6_·(H_2_O)_1.0_: C, 45.06; H, 4.32; N, 10.01. Found: C, 44.75, H, 4.46; N, 9.78; HRMS (EI) calcd for (C_21_H_21_BrN_4_O_6_ + H)^+^ 505.0723, found 505.0717.

#### l-BHDU-5′-*O*-l-leucine Ester Hydrochloride (**20**)

Yield: 89%; [α]_D_^24^ = −4.9 (*c* 0.5, MeOH); ^1^H NMR (500 MHz, CD_3_OD) δ 7.70 (s, 1H), 7.45 (d, *J* = 13.6 Hz, 1H), 6.90 (d, *J* = 13.6 Hz, 1H), 6.36 (dd, *J* = 5.8 & 1.8 Hz, 1H), 5.30 (t, *J* = 3.7 Hz, 1H), 4.62 (dd, *J* = 12.2 & 3.8 Hz, 1H), 4.50 (dd, *J* = 12.2 & 3.7 Hz, 1H), 4.43 (dd, *J* = 10.4 & 1.7 Hz, 1H), 4.24 (dd, *J* = 10.4 & 5.9 Hz, 1H), 4.06 (dd, *J* = 7.5 & 6.3 Hz, 1H), 1.87–1.70 (m, 3H), 1.00 (dd, *J* = 6.2 & 3.3 Hz, 6H); ^13^C NMR (125 MHz, CD_3_OD) δ 169.6, 162.0, 138.1, 129.0, 111.5, 108.5, 102.1, 81.7, 70.0, 64.1, 51.1, 39.2, 24.1, 21.2, 21.0; anal. calcd for C_16_H_23_BrClN_3_O_6_·(H_2_O)_1.0_: C, 39.48; H, 5.18; N, 8.63. Found: C, 39.29, H, 5.31; N, 8.41; HRMS (EI) calcd for (C_16_H_22_BrN_3_O_6_ + H)^+^ 432.0770, found 432.0774.

#### l-BHDU-5′-*O*-l-isoleucine Ester Hydrochloride (**21**)

Yield: 86%; [α]_D_^26^ = −1.9 (*c* 0.5, MeOH); ^1^H NMR (500 MHz, CD_3_OD) δ 7.73 (s, 1H), 7.46 (d, *J* = 13.6 Hz, 1H), 6.91 (d, *J* = 13.6 Hz, 1H), 6.37 (dd, *J* = 5.8 & 1.6 Hz, 1H), 5.30 (t, *J* = 3.4 Hz, 1H), 4.61 (dd, *J* = 12.3 & 3.3 Hz, 1H), 4.53 (dd, *J* = 12.3 & 3.5 Hz, 1H), 4.45 (dd, *J* = 10.4 & 1.5 Hz, 1H), 4.25 (dd, *J* = 10.4 & 5.9 Hz, 1H), 4.01 (d, *J* = 4.1 Hz, 1H), 2.03–1.96 (m, 1H), 1.56–1.46 (m, 1H), 1.39–1.31 (m, 1H), 1.06 (d, *J* = 7.0 Hz, 3H), 0.98 (t, *J* = 7.4 Hz, 3H); ^13^C NMR (125 MHz, CD_3_OD) δ 168.5, 162.1, 150.1, 138.3, 129.0, 111.5, 108.5, 102.2, 81.7, 70.0, 63.8, 57.3, 36.4, 24.9, 13.8, 10.6; anal. calcd for C_16_H_23_BrClN_3_O_6_: C, 41.00; H, 4.95; N, 8.96. Found: C, 40.82, H, 4.93; N, 8.74; HRMS (EI) calcd for (C_16_H_22_BrN_3_O_6_ + H)^+^ 432.0770, found 432.0767.

#### l-BHDU-5′-*O*-l-proline Ester Hydrochloride (**22**)

Yield: 85%; [α]_D_^25^ = −15.78 (*c* 0.5, MeOH); ^1^H NMR (500 MHz, CD_3_OD) δ 7.74 (s, 1H), 7.46 (d, *J* = 13.6 Hz, 1H), 6.92 (d, *J* = 14.1 Hz, 1H), 6.36 (dd, *J* = 5.8 & 1.7 Hz, 1H), 5.30 (t, *J* = 3.1 Hz, 1H), 4.62 (dd, *J* = 12.4 & 3.2 Hz, 1H), 4.55 (dd, *J* = 12.4 & 3.1 Hz, 1H), 4.47–4.42 (m, 2H), 4.24 (dd, *J* = 10.4 & 5.9 Hz, 1H), 3.48–3.42 (m, 1H), 3.40–3.36 (m, 1H), 2.44–2.36 (m, 1H), 2.19–2.07 (m, 3H); ^13^C NMR (125 MHz, CD_3_OD) δ 168.5, 162.1, 150.0, 138.3, 129.1, 111.4, 108.4, 102.2, 100.0, 81.7, 70.1, 64.0, 59.3, 28.1, 23.2; anal. calcd for C_15_H_19_BrClN_3_O_6_·(H_2_O)_1.0_: C, 38.28; H, 4.50; N, 8.93. Found: C, 38.30, H, 4.50; N, 8.82; HRMS (EI) calcd for (C_15_H_18_BrN_3_O_6_ + H)^+^ 416.0457, found 416.0457.

#### l-BHDU-5′-*O*-l-glutamine Ester Hydrochloride (**23**)

Yield: 87%; [α]_D_^25^ = +1.9 (*c* 0.5, MeOH); ^1^H NMR (500 MHz, CD_3_OD) δ 7.74 (s, 1H), 7.45 (d, *J* = 13.5 Hz, 1H), 6.91 (d, *J* = 13.6 Hz, 1H), 6.35 (dd, *J* = 5.8 & 1.8 Hz, 1H), 5.30 (t, *J* = 3.8 Hz, 1H), 4.61 (dd, *J* = 12.1 & 4.0 Hz, 1H), 4.52 (dd, *J* = 12.1 & 3.7 Hz, 1H), 4.43 (dd, *J* = 10.3 & 1.7 Hz, 1H), 4.24 (dd, *J* = 10.4 & 5.9 Hz, 1H), 4.16 (t, *J* = 6.3 Hz, 1H), 2.54–2.50 (m, 2H), 2.26–2.13 (m, 2H); ^13^C NMR (125 MHz, CD_3_OD) δ 168.7, 162.1, 150.0, 138.1, 129.0, 111.5, 108.5, 102.1, 100.0, 81.9, 70.1, 64.3, 52.3, 30.3, 25.5; anal. calcd for C_15_H_20_BrClN_4_O_7_·(H_2_O)_1.5_: C, 35.28; H, 4.54; N, 10.97. Found: C, 35.43, H, 4.63; N, 10.63; HRMS (EI) calcd for (C_15_H_19_BrN_4_O_7_ + H)^+^ 447.0515, found 447.0508.

#### l-BHDU-5′-*O*-2-aminoisobutyric Acid Ester Hydrochloride (**24**)

Yield: 82%; ^1^H NMR (500 MHz, CD_3_OD) δ 7.71 (s, 1H), 7.44 (d, *J* = 13.6 Hz, 1H), 6.90 (d, *J* = 13.6 Hz, 1H), 6.35 (dd, *J* = 5.9 & 1.8 Hz, 1H), 5.29 (t, *J* = 3.8 Hz, 1H), 4.60 (dd, *J* = 12.2 & 4.0 Hz, 1H), 4.54 (dd, *J* = 12.2 & 3.6 Hz, 1H), 4.44 (dd, *J* = 10.4 & 1.8 Hz, 1H), 4.25 (dd, *J* = 10.4 & 5.9 Hz, 1H), 1.63 (s, 3H), 1.61 (s, 3H); ^13^C NMR (125 MHz, CD_3_OD) δ 171.23, 162.1, 150.0, 138.0, 129.0, 111.5, 108.4, 102.2, 81.9, 70.1, 64.3, 56.6, 22.7; anal. calcd for C_14_H_19_BrClN_3_O_6_·(H_2_O)_1.0_: C, 36.66; H, 4.61; N, 9.16. Found: C, 36.94, H, 4.64; N, 8.77; HRMS (EI) calcd for (C_14_H_18_BrN_3_O_6_ + H)^+^ 404.0457, found 404.0451.

#### l-BHDU-5′-*O*-l-lysine Ester Hydrochloride (**25**)

Yield: 85%; [α]_D_^25^ = −1.6 (*c* 0.5, MeOH); ^1^H NMR (500 MHz, CD_3_OD) δ 7.74 (s, 1H), 7.46 (d, *J* = 13.5 Hz, 1H), 6.92 (d, *J* = 13.5 Hz, 1H), 6.37 (dd, *J* = 5.8 & 1.6 Hz, 1H), 5.32 (t, *J* = 3.6 Hz, 1H), 4.61 (dd, *J* = 12.2 & 3.7 Hz, 1H), 4.54 (dd, *J* = 12.2 & 3.4 Hz, 1H), 4.46 (dd, *J* = 10.4 & 1.6 Hz, 1H), 4.25 (dd, *J* = 10.4 & 5.9 Hz, 1H), 4.11 (t, *J* = 6.5 Hz, 1H), 3.02–2.95 (m, 2H), 2.06–1.89 (m, 2H), 1.78–1.72 (m, 2H), 1.62–1.48 (m, 2H); ^13^C NMR (125 MHz, CD_3_OD) δ 168.9, 162.2, 150.1, 138.4, 129.0, 111.4, 108.5, 102.2, 100.0, 70.0, 64.2, 52.4, 39.0, 29.7, 26.7, 21.7; anal. calcd for C_16_H_25_BrCl_2_N_4_O_6_·(H_2_O)_1.5_: C, 35.12; H, 5.16; N, 10.24. Found: C, 35.26, H, 5.28; N, 9.87; HRMS (EI) calcd for (C_16_H_23_BrN_4_O_6_ + H)^+^ 447.0879, found 447.0869.

#### (*S*)-2-((*tert*-Butoxycarbonyl)amino)-3-(4-((*tert*-butyldimethylsilyl)oxy)phenyl)propanoic Acid (**28**)

To a solution of *(tert-*butoxycarbonyl*)-*L*-*tyrosine (**27**, 0.50 g, 1.77 mmol) in DMF (10 mL) were added TBDMSCl (0.4 g, 2.65 mmol) and imidazole (0.24 g, 3.54 mmol) at 0 °C, then the reaction mixture was stirred at rt for 16 h. It was quenched with water (15 mL), and the aqueous layer was extracted with diethyl ether (25 mL × 2); the combined organic layer was washed with brine (25 mL), dried over Na_2_SO_4,_ and concentrated under reduced pressure. The crude residue was purified by silica gel chromatography (10–25% EtOAc/hexane) to give compound **28** as a viscous solid. Yield (0.44 g, 62%). ^1^H NMR (500 MHz, CDCl_3_) δ 7.04 (d, *J* = 8.0 Hz, 2H), 6.78 (d, *J* = 7.9 Hz, 2H), 4.89 (d, *J* = 8.0 Hz, 1H), 4.54 (s, 1H), 3.12 (d, *J* = 12.3 Hz, 1H), 3.00 (d, *J* = 14.6 Hz, 1H), 1.41 (s, 9H), 0.97 (s, 9H), 0.18 (s, 6H); HRMS (EI) calcd For (C_20_H_33_NO_5_Si+Na)^+^ 418.2026, found *m*/*z* 418.2033.

#### ((2*S*,4*S*)-4-(5-((*E*)-2-Bromovinyl)-2,4-dioxo-3,4-dihydropyrimidin-1(2*H*)-yl)-1,3-dioxolan-2-yl)methyl (*S*)-2-((*tert*-Butoxycarbonyl)amino)-3-(4-((*tert*-butyldimethylsilyl)oxy)phenyl) Propanoate (**29**)

Compound **28** was coupled with l-BHDU by following the above-described coupling procedure for compounds **2**–**13**. Yield: 80%; ^1^H NMR (500 MHz, CDCl_3_) δ 8.54 (bs, 1H), 7.55 (s, 1H), 7.48 (d, *J* = 13.6 Hz, 1H), 6.99 (d, *J* = 9.3 Hz, 3H), 6.76 (d, *J* = 8.2 Hz, 1H), 6.31 (d, *J* = 5.6 Hz, 1H), 5.12 (t, *J* = 3.3 Hz, 1H), 4.90 (d, *J* = 7.4 Hz, 1H), 4.61–4.53 (m, 1H), 4.47 (d, *J* = 8.0 Hz, 1H), 4.28–4.09 (m, 3H), 3.05–2.91 (m, 2H), 1.41 (s, 9H), 0.97 (s, 9H), 0.10 (s, 6H); HRMS (EI) calcd for (C_30_H_42_BrN_3_O_9_Si + H)^+^ 696.1952, found *m*/*z* 696.1957.

#### ((2*S*,4*S*)-4-(5-((*E*)-2-Bromovinyl)-2,4-dioxo-3,4-dihydropyrimidin-1(2*H*)-yl)-1,3-dioxolan-2-yl)methyl (*tert*-Butoxycarbonyl)-l-tyrosinate (**30**)

To a stirred solution of compound **29** (100 mg, 0.143 mmol) in THF (5 mL) was added 1 M TBAF in THF (0.21 mL) at 0 °C. The mixture was warmed to rt and stirred for 3 h. Then, the mixture was quenched with water (10 mL), and the aqueous layer was extracted with EtOAc (15 mL × 2); the combined organic layer was washed with brine (15 mL), dried over Na_2_SO_4_, and concentrated under reduced pressure. The obtained crude compound was purified by silica gel chromatography (45–50% EtOAc/hexane) to give **30** as a sticky solid. Yield (65 mg, 78%). ^1^H NMR (500 MHz, CDCl_3_) δ 9.95 (bs, 1H), 7.50–7.37 (m, 2H), 6.92 (d, *J* = 7.7 Hz, 2H), 6.72 (d, *J* = 9.9 Hz, 3H), 6.28 (s, 1H), 5.11 (d, *J* = 7.5 Hz, 1H), 5.03 (s, 1H), 4.44 (d, *J* = 12.5 Hz, 2H), 4.32–4.18 (m, 2H), 4.15–4.07 (m, 1H), 2.99–2.89 (m, 2H), 1.42 (s, 9H); ^13^C NMR (125 MHz, CDCl_3_) δ 174.3, 161.7, 155.4, 149.6, 137.3, 130.3, 128.2, 126.9, 115.6, 112.3, 110.9, 102.6, 81.3, 80.5, 70.9, 62.9, 54.9, 53.5, 37.3, 28.5; HRMS (EI) calcd for (C_24_H_28_BrN_3_O_9_ – H)^−^ 580.0931, found 580.0936.

#### l-BHDU-5′-*O*-l-tyrosine Ester Hydrochloride (**26**)

To a stirred solution of compound **30** (100 mg, 0.171 mmol) in DCM (5 mL) at 0 °C was added TFA (1 mL), and the mixture was stirred at rt for 1 h. After checking TLC, the volatiles were removed under reduced pressure. The obtained residue was dissolved in methanol (3 mL), cooled to 0 °C, and a solution of 2 M HCl was added in ether and stirred for 30 min. The volatiles were distilled off under reduced pressure, and the crude solid was washed with anhydrous ether to obtain **26** as the HCl salt as a white solid. Yield (75 mg, 85%). [α]_D_^25^ = −2.4 (*c* 0.5, MeOH); ^1^H NMR (500 MHz, CD_3_OD) δ 7.68 (s, 1H), 7.40 (d, *J* = 13.5 Hz, 1H), 7.03 (d, *J* = 5.9 Hz, 2H), 6.87 (d, *J* = 13.1 Hz, 1H), 6.79–6.74 (m, 2H), 6.34 (dd, *J* = 5.9 & 1.7 Hz, 1H), 5.22 (t, *J* = 3.3 Hz, 1H), 4.56 (dd, *J* = 12.4 & 3.4 Hz, 1H), 4.49 (dd, *J* = 12.4 & 3.2 Hz, 1H), 4.42 (dd, *J* = 10.4 & 1.6 Hz, 1H), 4.28–4.19 (m, 2H), 3.17–3.04 (m, 2H); ^13^C NMR (125 MHz, CD_3_OD) δ 168.7, 162.1, 157.1, 150.1, 138.2, 130.3, 129.0, 123.7, 115.7, 111.5, 108.5, 102.2, 81.7, 70.0, 63.7, 54.0, 35.2; anal. calcd for C_19_H_21_BrClN_3_O_7_·(H_2_O)_1.0_: C, 42.52; H, 4.32; N, 7.83. Found: C, 42.54, H, 4.42; N, 7.68; HRMS (EI) calcd for (C_19_H_20_BrN_3_O_7_ – H)^−^ 480.0406, found 480.0410.

#### Isopropyl (Chloro(phenoxy)phosphoryl)-l-alaninate (**31**)

To a solution of phenyl dichlorophosphate (1.0 mol) and l-alanine isopropyl ester hydrochloride (1.0 mol) in anhydrous DCM at −78 °C, triethylamine (2.0 mol) was added dropwise, and the mixture was stirred at the same temperature for 1 h. The reaction mixture was slowly warmed to rt and stirred for 2 h. The solvent was removed under reduced pressure, and the crude residue was resuspended in anhydrous ether and filtered through a Celite bed under nitrogen. The filtrate was concentrated in vacuo to give compound **31** as an oily liquid, which was used as such for the next step. ^1^H NMR (500 MHz, CDCl_3_) δ 7.38–7.24 (m, 2H), 7.23–7.21 (m, 3H), 5.11–5.04 (m, 1H), 4.33–4.07 (m, 2H), 1.49 (d, *J* = 10.0 Hz, 3H), 1.26 (dd, *J* = 15.0 & 5.0 Hz, 6H); ^31^P NMR (CDCl_3_, 202 MHz): δ 8.69, 8.31.

#### Isopropyl (Chloro(phenoxy)phosphoryl)-l-phenylalaninate (**32**)

To a solution of phenyl dichlorophosphate (1.0 mol) and the l-phenylalanine isopropyl ester hydrochloride (1.0 mol) in anhydrous DCM at −78 °C, triethylamine (2.0 mol) was added dropwise, and the mixture was stirred at the same temperature for 1 h. The reaction mixture was slowly warmed to rt and stirred for 2 h. The solvent was removed under reduced pressure, and the crude residue was resuspended in anhydrous ether and filtered through a Celite bed under nitrogen. The filtrate was concentrated in vacuo to give compound **32** as an oily liquid, which was used as such for the next step. ^1^H NMR (500 MHz, CDCl_3_) δ 7.37–7.14 (m, 10H), 5.04–4.95 (m, 1H), 4.42–4.30 (m, 1H), 4.17–4.03 (m, 1H), 3.18–3.06 (m, 1H), 1.19 (dd, *J* = 10.0 & 5.0 Hz, 6H); ^31^P NMR (CDCl_3_, 202 MHz): δ 8.63, 8.60.

#### Isopropyl ((((2*S*,4*S*)-4-(5-((*E*)-2-Bromovinyl)-2,4-dioxo-3,4-dihydropyrimidin-1(2*H*)-yl)-1,3-dioxolan-2-yl)methoxy)(phenoxy)phosphoryl)-l-alaninate (**33**)

To a stirred suspension of l-BHDU, **1** (0.1 g, 0.31 mmol) in anhydrous THF (8 mL) and *N-*methylimidazole (0.15 mL, 1.86 mmol) were added at 0 °C. Phosphorochloridate **31** (0.37 g, 1.24 mmol) in THF (10 mL) was added dropwise, and the mixture was stirred for 12 h at ambient temperature. The volatiles were removed under reduced pressure, and the residue was purified by silica gel column chromatography (2% methanol/DCM) to give compound **33** as an off-white solid. Yield (0.12 g, 66%). Mp 63–65 °C; ^1^H NMR (500 MHz, CDCl_3_) δ 7.71–7.65 (m, 1H), 7.45–7.41 (m, 1H), 7.31–7.28 (m, 2H), 7.25–7.18 (m, 1H), 7.13 (t, *J* = 15.0 & 5.0 Hz, 1H), 6.87 (s, 1H), 6.70 (dd, *J* = 10.0 & 5.0 Hz, 1H), 6.30 (dd, *J* = 5.0 & 2.5 Hz, 1H), 5.17–5.15 (m, 2H), 5.01–4.95 (m, 1H), 4.46–4.21 (m, 2H), 4.18–4.14 (m, 2H), 4.02–3.67 (m, 2H), 1.32 (d, *J* = 5.0 Hz, 3H), 1.19 (dd, *J* = 10.0 & 5.0 Hz, 6H); ^31^P NMR (CDCl_3_, 202 MHz): δ 4.07, 3.73; ^13^C NMR (125 MHz, CDCl_3_) δ 173.0, 161.4, 150.4, 149.6, 137.3, 129.9, 129.5, 128.4, 125.2, 120.3, 112.1, 110.6, 103.3, 81.4, 71.9, 69.5, 64.2, 50.5, 33.4, 21.8, 21.0; anal. calcd for C_22_H_27_BrN_3_O_9_P: C, 44.91; H, 4.63; N, 7.14. Found: C, 45.23, H, 4.92; N, 6.85; HRMS (EI) calcd for (C_22_H_27_BrN_3_O_9_P + H)^+^ 588.0747; found 588.0743.

#### Isopropyl ((((2*S*,4*S*)-4-(5-((*E*)-2-Bromovinyl)-2,4-dioxo-3,4-dihydropyrimidin-1(2*H*)-yl)-1,3-dioxolan-2-yl)methoxy)(phenoxy)phosphoryl)-l-phenylalaninate (**34**)

To a stirring suspension of l-BHDU, **1** (0.1 g, 0.31 mmol) in anhydrous THF and *N-*methylimidazole, (NMI, 0.15 mL, 1.86 mmol), were added at 0 °C. Phosphorochloridate **32** (0.47 g, 1.24 mmol) in THF (10 mL) was added dropwise, and the mixture was stirred for 12 h at ambient temperature. The volatiles were removed under reduced pressure, and the residue was purified by silica gel column chromatography (1.5% methanol/DCM) to give compound **34** as a white solid. Yield (0.12g, 57%). Mp 71–73 °C; ^1^H NMR (500 MHz, CDCl_3_) δ 8.27 (m, 1H), 7.64 (d, *J* = 5.0 Hz, 1H), 7.45–7.39 (m, 1H), 7.31–7.29 (m, 2H), 7.28–7.02 (m, 8H), 6.70 (d, *J* = 15.0 Hz, 1H), 6.29–6.26 (m, 1H), 5.08–5.07 (m, 1H), 4.97–4.91 (m, 1H), 4.30–4.05 (m, 5H), 3.52–3.34 (m, 1H), 3.02–2.91 (m, 2H), 1.17 (dd, *J* = 10.0 & 5.0 Hz, 6H); ^31^P NMR (CDCl_3_, 202 MHz): δ 3.91, 3.82; ^13^C NMR (125 MHz, CDCl_3_) δ 171.9, 161.0, 150.4, 149.3, 137.3, 135.5, 129.8, 129.7, 129.6, 128.6, 128.3, 127.2, 125.3, 125.2, 120.2, 112.1, 110.6, 103.4, 81.6, 71.6, 69.6, 63.8, 55.6, 40.4, 21.8; anal. calcd for C_28_H_31_BrN_3_O_9_P: C, 50.61; H, 4.70; N, 6.32. Found: C, 50.92, H, 4.95; N, 6.11; HRMS (EI) calcd for (C_28_H_31_BrN_3_O_9_P + H)^+^ 664.1060; found 664.1056.

#### l-BHDU-5′-[(2-octadecyloxyethyl)phosphate] (ODE-l-BHDU-MP, **38**)

A solution of 1,2,4-triazole (0.28 g, 4.1 mmol) and triethylamine (0.57 mL, 4.1 mmol) in anhydrous THF (10 mL) was added to a solution of 2-chlorophenyl dichlorophosphate (**35**, 0.5 g, 2.0 mmol) in THF (10 mL). The reaction mixture was stirred at rt for 30 min and filtered. In another flask, to a prestirred (20 min.) solution of l-BHDU (**1**, 0.49 g, 1.5 mmol) in dry THF (20 mL), NMI (0.17 mL, 2.0 mmol) was added above the filtrate intermediate at 0 °C and the mixture was stirred at rt for 1 h. Then, 2-(octadecyloxy)ethanol (0.48 g, 1.5 mmol) was added to the mixture and stirred overnight at rt. The solvent was evaporated under reduced pressure, and the obtained crude was purified by silica gel column chromatography (3% MeOH/DCM) to produce l-BHDU 5′-[(2-chlorophenyl 2-octadecyloxyethyl) phosphate] **36** as a white solid. Yield (0.85 g, 69%). ^1^H NMR (500 MHz, CDCl_3_) δ 9.13 (bs, 1H, NH), 7.71 (d, *J* = 2.5 Hz, 1H), 7.48–7.38 (m, 3H), 7.20 (t, *J* = 16.0 Hz, 1H), 7.10 (t, *J* = 15.5 Hz, 1H), 6.72 (dd, *J* = 13.5 & 4.5 Hz, 1H); 6.33 (dd, *J* = 16.5 & 7.5 Hz, 1H), 5.17–5.16 (m, 1H), 4.55–4.43 (m, 2H), 4.37–4.32 (m, 2H), 4.22–4.16 (m, 2H), 3.66–3.64 (m, 2H), 3.42 (t, *J* = 13.5 & 8.0 Hz, 2H), 1.52–1.48 (m, 2H), 1.29–1.23 (m, 30H), 0.86 (t, *J* = 7.0 Hz, 3H); ^31^P NMR (202 MHz, CDCl_3_): δ −6.10, 6.36; ^13^C NMR (125 MHz, CDCl_3_) δ 160.9, 149.3, 146.3, 130.9, 130.5, 128.2, 127.6, 126.6, 126.0, 125.5, 112.1, 110.8, 110.4, 81.5, 81.4, 81.2, 71.5, 71.4, 68.3, 31.9, 29.7, 29.5, 29.4, 26.0, 22.7, 14.2. The obtained intermediate **36** (0.45 g, 0.55 mmol) was dissolved in THF (5 mL), and 0.5 N NaOH solution (1.5 mL) was added at 0 °C. The mixture was stirred at 50 °C for 2 h and neutralized with 1 N HCl at 0 °C. The volatiles were removed under reduced pressure, and the residue was purified by silica gel column chromatography (10% MeOH/DCM) to give **38** as a white solid. Yield (0.29 g, 74%). Mp 115–117 °C; ^1^H NMR (500 MHz, DMSO-*d*_6_) δ 11.59 (s, 1H), 8.20 (s, 1H), 7.39 (d, *J* = 13.5 Hz, 1H), 7.26 (d, *J* = 14.0 Hz, 1H), 6.18 (d, *J* = 4.5 Hz, 1H), 5.04–5.03 (m, 1H), 4.23 (d, *J* = 9.5 Hz, 1H), 4.08 (t, *J* = 4.5 Hz, 1H), 3.91–3.93 (m, 2H), 3.72–3.69 (m, 2H), 3.43 (t, *J* = 4.5 Hz, 2H), 3.35–3.34 (m, 2H), 1.46–1.43 (m, 2H), 1.28–1.23 (m, 30H), 0.85 (t, *J* = 8.6 Hz, 3H); ^31^P NMR (202 MHz, DMSO-*d*_6_): δ −0.7; ^13^C NMR (125 MHz, DMSO-*d*_6_) δ 162.3, 150.0, 139.4, 130.6, 110.9, 107.7, 103.2, 81.3, 70.8, 69.6, 66.0, 64.6, 31.9, 29.7, 29.6, 29.4, 29.2, 26.1, 22.6, 14.5; anal. calcd for C_30_H_52_BrN_2_O_9_P·(H_2_O)_0.5_: C, 51.14; H, 7.58; N, 3.98. Found: C, 51.20, H, 7.55; N, 3.92; HRMS (EI) calcd for (C_30_H_52_BrN_2_O_9_P + Na)^+^ 717.2492, found 717.2485.

#### l-BHDU-5′-[(2-hexadecyloxyethyl)phosphate] (HDP-l-BHDU-MP, **39**)

Compound **39** (50 mg) was synthesized in qualitative yield by following the same procedure explained for compound **38**. Yield 85%; mp 122–123 °C; ^1^H NMR (500 MHz, CD_3_OD) δ 8.02 (s, 1H), 7.46 (d, *J* = 14.0 Hz, 1H), 7.04 (d, *J* = 13.5 Hz, 1H), 6.32 (dd, *J* = 7.5 & 1.5 Hz, 1H), 5.19 (s, 1H), 4.29 (dd, *J* = 7.0 & 2.0 Hz, 1H), 4.22–4.19 (m, 1H), 4.15–4.14 (m, 2H), 3.99 (d, *J* = 6.5 & 2.0 Hz, 2H), 3.55 (t, *J* = 7.0 Hz, 2H), 3.43 (t, *J* = 6.5 Hz, 2H), 1.92–1.89 (m, 2H), 1.56–1.52 (m, 2H), 1.37–1.31 (m, 26H), 0.93 (t, *J* = 7.0 Hz, 3H); ^31^P NMR (202 MHz, CD_3_OD): δ 0.58; ^13^C NMR (125 MHz, CD_3_OD) δ 162.3, 150.0, 138.6, 129.6, 129.1, 111.2, 108.3, 107.8, 104.5, 104.0, 81.7, 71.1, 70.7, 67.0, 63.6, 62.4, 31.7, 30.8, 30.7, 30.6, 29.4, 29.3, 29.1, 25.9, 22.4, 13.0; anal. calcd for C_29_H_50_BrN_2_O_9_P·(H_2_O)_0.9_: C, 49.92; H, 7.48; N, 4.01. Found: C, 49.65, H, 7.62; N, 3.69; HRMS (EI) calcd for (C_29_H_50_BrN_2_O_9_P + H)^+^ 681.2516, found 681.2507.

#### Bis(POM) Prodrug of l-BHDU-MP (POM-l-BHDU-MP, **41**)

To a stirred solution of l-BHDU (**1**, 80 mg, 0.25 mmol) and *N*-methylimidazole (0.16 mL, 2.0 mmol) in dry THF (3 mL), bis(POM)phosphorochloridate^31^**40** (500 mg, 1.23 mmol) at 0 °C was added by dissolving in dry THF (3 mL) and stirred for 15 min. Then, the reaction was warmed to rt and stirred for 3 h. The mixture was quenched with methanol, and the solvent was removed under reduced pressure. The crude was purified by silica gel column chromatography (0.5% MeOH/DCM) to give **41** as a colorless sticky oil, which was crystallized in DCM-pentane to render an off-white solid. Yield: (75 mg, 47%). Mp: 80–85 °C; ^1^H NMR (500 MHz, CDCl_3_) δ 8.97 (bs, 1H), 7.71 (s, 1H), 7.45 (d, *J* = 13.6 Hz, 1H), 6.79 (d, *J* = 13.6 Hz, 1H), 6.35 (d, *J* = 4.5 Hz, 1H), 5.72–5.65 (m, 4H), 5.15 (s, 1H), 4.43–4.39 (m, 1H), 4.35–4.31 (m, 1H), 4.26–4.21 (m, 1H), 4.20–4.17 (m, 1H), 1.23 (s, 18H); ^31^P NMR (202 MHz, CDCl_3_) δ −3.02; ^13^C NMR (125 MHz, CDCl_3_) δ 176.9, 161.1, 149.5, 137.3, 128.4, 112.2, 110.6, 102.9, 83.2, 81.4, 71.6, 65.1, 38.9, 26.9; anal. calcd for C_22_H_32_BrN_2_O_12_P: C, 42.12; H, 5.14; N, 4.47. Found: C, 42.34, H, 5.23; N, 4.26; HRMS (EI) calcd for (C_22_H_32_BrN_2_O_12_P + H)^+^ 627.0954, found *m*/*z* 627.0953.

#### ((Bis(benzyloxy)phosphoryl)oxy)methyl Isopropyl Carbonate (**43**)

To a stirred mixture of compound **42** (560 mg, 2.0 mmol) and cesium carbonate (1.6 g, 4.97 mmol) in acetone (10 mL), POC-I (610 mg, 2.38 mmol) was added dropwise at rt and stirred overnight. The reaction mixture was filtered through a Buchner funnel, the obtained filtrate was concentrated under reduced pressure, and the residue was purified by silica gel column chromatography (20% EtOAc/hexane) to produce compound **43** as a colorless oil. Yield (650 mg; 82%). ^1^H NMR (500 MHz, CDCl_3_) δ 7.35–7.30 (m, 10H), 5.61–5.58 (d, *J* = 15.0 Hz, 2H), 5.07–5.05 (d, *J* = 10.0 Hz, 4H), 4.90–4.85 (m, 1H), 1.29–1.28 (d, *J* = 5.0 Hz, 6H); ^31^P NMR (202 MHz, CDCl_3_) δ −2.02; HRMS (EI) calcd for (C_19_H_23_O_7_P + Na)^+^ 417.1079, found *m*/*z* 417.1085.

#### (((Benzyloxy)(hydroxy)phosphoryl)oxy)methyl Isopropyl Carbonate (**44**)

To a stirred solution of compound **43** (1.0 g, 2.54 mmol) in acetonitrile (20 mL), NaI (0.76 g, 5.07 mmol) was added and stirred at 45 °C for 12 h. The reaction mixture was concentrated under reduced pressure, and the obtained crude was washed with dry ether and dried under high vacuum. The obtained residue was used as such for the next step without further purification.

#### ((Benzyloxyphosphoryl)bis(oxy))bis(methylene) Diisopropyl Bis(carbonate) (**45**)

To a stirred mixture of compound **44** (231 mg, 0.75 mmol) and cesium carbonate (371 mg, 1.13 mmol) in acetone (10 mL), POC-I (240 mg, 0.98 mmol) was added dropwise at rt and continued stirring overnight. The reaction mixture was filtered through a Buchner funnel, the obtained filtrate was concentrated under reduced pressure, and the residue was purified by silica gel column chromatography (15% EtOAc/hexane) to produce **45** as a colorless oil. Yield (210 mg, 66%) ^1^H NMR (500 MHz, CDCl_3_) δ 7.38–7.33 (m, 5H), 5.64–5.62 (d, *J* = 10.0 Hz, 4H), 5.13–5.12 (d, *J* = 5.0 Hz, 2H), 4.94–4.86 (s, 2H), 1.30–1.28 (dd, *J* = 10.0 Hz, 12H); ^31^P NMR (202 MHz, CDCl_3_) δ −3.77; HRMS (EI) calcd for (C_17_H_25_O_10_P + H)^+^ 420.1185, found *m*/*z* 420.1192.

#### ((Hydroxyphosphoryl)bis(oxy))bis(methylene) Diisopropyl Bis(carbonate) (**46**)

A suspension of compound **45** (300 mg, mmol) and 10% Pd/C (30 mg) in methanol at ambient temperature was treated with H_2_ at 5 psi for 2 h. The mixture was passed through a Celite bed and concentrated under reduced pressure to give **46** as a colorless sticky liquid. Yield (200 mg, 85%). Compound **46** was used as such for the next step reaction without further purification. ^1^H NMR (500 MHz, CDCl_3_) δ 7.99 (bs, 1H), 5.63–5.60 (d, *J* = 15.0 Hz, 4H), 4.95–4.88 (s, 2H), 1.31–1.30 (d, *J* = 5.0 & 2.0 Hz, 12H); ^13^C NMR (125 MHz, CDCl_3_) δ 153.1, 85.4, 73.4, 21.7; ^31^P NMR (202 MHz, CDCl_3_) δ −3.36; HRMS (EI) calcd for (C_10_H_19_O_10_P + Na)^+^ 353.0614, found *m*/*z* 353.0621.

#### Bis(POC) Prodrug of l-BHDU-MP (POC-l-BHDU-MP, **47**)

Compound **46** (92 mg, 0.282 mmol) was taken in NEt_3_ (1 mL) and pyridine (0.5 mL), stirred at rt for 10 min, and then the contents were concentrated under reduced pressure followed by co-evaporation with toluene (3 mL). The residue was dissolved in dry THF (3 mL) and cooled to 0 °C. After that, l-BHDU (30 mg, 0.094) was added, followed by the addition of DIPEA (0.05 mL, 0.282 mmol), BOP-Cl (48.0 mg, 0.189 mmol), and 3-nitro-1,2,4-triazole sequentially (21 mg, 0.189 mmol). The mixture was stirred at the same temperature for 2 h and diluted with EtOAc (50 mL). The organic layer was washed with saturated NaHCO_3_ solution (20 mL × 2), followed by brine solution (10 mL), and dried over Na_2_SO_4_. The solvent was removed under reduced pressure, and the obtained crude was purified by silica gel column chromatography (0.8% Methanol/DCM) to give compound **47** as a colorless sticky solid. Yield (13 mg, 22%). ^1^H NMR (500 MHz, CDCl_3_) δ 8.92 (s, 1H), 7.70 (s, 1H), 7.44 (d, *J* = 13.6 Hz, 1H), 6.78 (d, *J* = 13.6 Hz, 1H), 6.36 (dd, *J* = 5.6 & 2.0 Hz, 1H), 5.73–5.64 (m, 4H), 5.16 (d, *J* = 2.0 Hz, 1H), 4.92 (dq, *J* = 12.4 & 6.2 Hz, 2H), 4.46–4.33 (m, 2H), 4.28–4.17 (m, 2H), 1.33–1.30 (m, 12H); ^31^P NMR (202 MHz, CDCl_3_) −3.2; ^13^C NMR (125 MHz, CDCl_3_) δ 161.0, 153.0, 149.4, 137.3, 128.3, 112.2, 110.5, 102.9, 85.8, 81.4, 77.4, 73.7, 71.5, 65.2, 21.7; HRMS (EI) calcd for (C_20_H_28_BrN_2_O_14_P + H)^+^ 631.0540, found 631.0538; HPLC purity ≥ 97%.

### Cells and Viruses

Adherent retinal pigmented epithelial cells (ARPE-19; CRL-2302; ATCC) were used for all cell culture-based assays and can be used for at least 30 passages. Cells were grown in Dulbecco’s Modified Eagle Medium with 4.5 g/l-glucose, l-glutamine, and sodium pyruvate (DMEM, 1X, Corning, Manassas, VA) supplemented with 10% heat-inactivated fetal bovine serum (Benchmark FBS; Gemini Bio Products, West Sacramento, CA), penicillin–streptomycin (5000 IU/mL), and amphotericin B (250 μg/mL). The VZV strains used for the cell-based assays included VZV-ORF57-Luc, along with the mutant strains VZV-ORF57-ΔTK, VZV-ORF57-ΔTS, and VZV-ORF57-ΔTKTS.^34^ All viruses were passaged no more than 10 times in ARPE-19 cells.

### Compounds and Formulations

All compounds were prepared as 10 mM stocks in DMSO and stored at −80 °C. The control compounds, cidofovir (CDV; BEI Resources, Manassas, VA) and acyclovir (ACV; Millipore Sigma, Burlington, MA), are commercially available. Stock compounds were diluted in DMSO and/or complete tissue culture media prior to being added to cells.

### Efficacy and Cytotoxicity Assays in ARPE-19 Cells

Antiviral activity against the VZV strains used here was evaluated in ARPE-19 cells as previously described.^16,36^ Briefly, confluent ARPE-19 cells in a 96-well plate were infected with VZV at an approximate MOI of 0.01 for 2 h. Virus inoculum was removed, then the infected cells were treated with l-BHDU, its prodrugs, or the control compounds, CDV or ACV, at a range of concentrations. Virus spread was measured by bioluminescence (Total Flux) after 72 h. VZV yield was calculated as the average total flux for each concentration divided by the average Total Flux of the untreated wells. Cytotoxicity studies were performed using a neutral red assay as previously described.^16,37^ Cytotoxicity was measured after 72 h of co-culture with the test compounds under the same conditions as the antiviral evaluations at higher concentration ranges.

### Bioluminescence Imaging

Imaging was performed using the IVIS 50 instrument (Caliper Life Sciences/Xenogen, Hopkinton, Massachusetts) as previously described.^37^ Infected 96-well plates were imaged for 30 s^–1^ min, and VZV spread was measured as Total Flux (photons/s/cm^2^/steradian) in each well or region of interest (ROI).

### Statistical Analysis

Calculations for the 50% effective concentrations (EC_50_) and 50% cytotoxic concentrations (CC_50_) were performed using GraphPad software (San Diego, California, www.graphpad.com). Other calculations and graphs were also performed using GraphPad. A *p*-value of ≤0.05 was considered statistically significant.

### Procedure for Liver Homogenate Samples

Untreated BALB/c mice were euthanized, and their livers were immediately removed. Liver tissue was homogenized using a Qiagen TissueLyser LT (Qiagen LLC, Germantown MD). Exactly 0.95 mL of liver homogenate was combined with POM-l-BHDU-MP (0.3 μg/mL) and spiked with the internal standard azidothymidine (a final concentration of 20 ng/mL, Cayman Chemical, Ann Arbor, MI). The samples were incubated at 37 °C, and the enzymatic reactions were stopped by adding 2× volumes of methanol at 0, 2, 4, 10, 30, and 60 min. The samples were vortexed and centrifuged to remove the precipitated protein. Supernatants were dried using an N-EVAP 116 analytical nitrogen evaporator and reconstituted with 50% methanol in water. The concentration of POM-l-BHDU-MP, l-BHDU, and l-BHDU-MP were determined using LC-MS/MS.

### Collection of Mouse Plasma Samples for PK Analysis

This animal protocol #282 was approved by the IACUC at SUNY Upstate Medical University, which has been fully accredited by AAALAC since 7/31/1999. This method was previously used to evaluate l-BHDU effects on 5-fluorouracil metabolism.^16^ Briefly, a single equimolar dose of 22.5 mg/kg POM-l-BHDU-MP or 11.4 mg/kg l-BHDU was dissolved in Cremophor–DMSO–saline (1:1:8) and administered by oral gavage to two groups of BALB/c mice (Charles River) with equal numbers of male and female mice (*N* = 15 each male and female). Six untreated mice were used for blank controls and were labeled time 0. At 20, 40, 60, and 120 min, three mice from the POM-l-BHDU-MP group and three mice from the l-BHDU group were anesthetized with inhaled isoflurane and then exsanguinated by cardiac puncture. Blood samples were collected into a tube containing heparin. Plasma was separated from red blood cells by centrifugation at 10,000 rpm for 5 min. Plasma was transferred to a new tube and immediately frozen at −80 °C.

### Plasma Preparation for LC-MS/MS

Plasma samples were thawed, and exactly 0.95 mL was removed for analysis. The internal standard azidothymidine was added to mouse plasma samples at a final concentration of 20 ng/mL. Proteins were precipitated from the plasma by adding 2× volumes of methanol, then the samples were clarified by centrifugation. The supernatants were dried using an N-EVAP 116 analytical nitrogen evaporator and then reconstituted with 50% methanol in water. LC-MS/MS was used to analyze the concentrations of POM-l-BHDU-MP, l-BHDU, and l-BHDU-MP. Calibration curves were made using BALB/c mouse plasma (Innovative Research, Inc. Novi MI) prior to analyzing the experimental samples.

### Protocol of LC/MS Analysis

The primary stock solutions for the calibration were prepared at 0.5 mg/mL for l-BHDU, 1 mg/mL for POM-l-BHDU-MP, and 1 mg/mL for l-BHDU-MP. The primary stock solutions were diluted to working solutions that were in turn used to spike the biological matrix of interest. l-BHDU calibration points were 100.0, 400.0, 800.0, 1000.0, 4000.0, 6000.0, 8000.0, 10,000.0, and 12,000.0 ng/mL. l-BHDU-MP calibration points were 100.0, 200.0, 400.0, 800.0, 1000.0, 2000.0, 4000.0, 6000.0, and 8000.0 ng/mL. The ISTD working solution of AZT was prepared at 20 ng/mL. The standard stock solutions were stored in −80 °C freezer until used. A Waters Xevo Micro TQS UPLC Mass spectrometer with an ESI (−) source was operated for the LC/MS analysis. Waters MassLynx 4.2 software (Milford, MA) was used for instrumentation and obtaining data. An Acquity Premier Oligonucleotide BEH C18, 130 Å, 1.7 μm 2.1 × 150 mm column coupled with an Acquity UPLC BEH C18 1.7 μm (2.1 × 5 mm) VanGuard Pre-Column was used to separate the analytes. The column temperature was set at 30 °C, and the autosampler was set at 5 °C. The mobile phase A was 5 mM ammonium formate in water, and the mobile phase B was 5 mM ammonium formate in methanol. The injection volume was 5 μL. The analytes were separated using a gradient method, with a 0.15 mL/min flow rate (time/minute, % mobile phase b): (0/10), (2/10), (20/95), (20.1/10), and (30,10). The samples were analyzed in a negative ESI mode. The capillary voltage was 1.50 kV, and the cone voltage was 25 V. The desolvation gas was nitrogen and was used at a flow rate of 650 L/h. The desolvation temperature was 350 °C, and the source temperature was 150 °C. The collision gas was argon with a collision cell pressure of 3.18 × 10^–3^ mbar, and the collision energy was 30 V. A multiple reaction monitoring (MRM) function was used to quantify the analytes using the following ion transitions: 266.1 → 223.2 for AZT (ISTD), 318.7 → 80.7 for l-BHDU, 398.7 → 180.9 for l-BHDU-MP, and 626.6 → 325 for POM-l-BHDU-MP.

## Supporting Information Available

^1^H, ^13^C, and ^31^P NMR spectra of all intermediates and final compounds, elemental analysis of compounds (Table S1); HPLC methods and trace for compounds **1**, **24**, **34**, **38**, **39**, **41**, and **47** (PDF)
Molecular formula strings (CSV)

## Abbreviations

ACV: acyclovir
BVU: bromovinyluracil
CDV: cidofovir
DPD: dihydropyrimidine dehydrogenase
DIC: 1,3-diisopropylcarbodiimide
DIPEA: *N*,*N*-diisopropylethylamine
DMAP: 4-(dimethylamino)pyridine
Et_3_N: triethylamine
FCV: famciclovir
5-FU: 5-fluorouracil
HFFs: human foreskin fibroblasts
HBV: hepatitis B virus
HDP: hexadecyloxypropyl
HIV: human immunodeficiency virus
NMP: nucleoside monophosphate
NMI: *N*-methylimidazole
ODE: octadecyloxyethyl
POM: bis(pivaloyloxymethyl)
POC: bis(isopropyloxymethyl carbonate)
TK: thymidine kinase
TBDMSCl: *tert*-butyldimethylsilyl chloride
TBAF: tetrabutylammonium fluoride
TFA: trifluoro acetic acid
VZV: varicella zoster virus
VACV: valacyclovir

## References

[ref1] Gershon A. A.; Breuer J.; Cohen J. I.; Cohrs R. J.; Gershon M. D.; Gilden D.; Grose C.; Hambleton S.; Kennedy P. G. E.; Oxman M. N.; Seward J. F.; Yamanishi K. Varicella Zoster Virus Infection. Nat. Rev. Dis. Primers 2015, 1, 15016 10.1038/nrdp.2015.16. 27188665 PMC5381807

[ref2] Marin M.; Leung J.; Gershon A. A. Transmission of Vaccine-Strain Varicella-Zoster Virus: A Systematic Review. Pediatrics 2019, 144, e20191305 10.1542/peds.2019-1305. 31471448 PMC6957073

[ref3] Bharucha T.; Ming D.; Breuer J. A Critical Appraisal of ″Shingrix’, A Novel Herpes Zoster Subunit Vaccine (HZ/Su or GSK1437173A) for Varicella Zoster Virus. Hum. Vaccines Immunother. 2017, 13, 1789–1797. 10.1080/21645515.2017.1317410. PMC5557227 28426274

[ref4] https://www.cdc.gov/shingles/index.html.

[ref5] Bruxelle J.; Pinchinat S. Effectiveness of Antiviral Treatment on Acute Phase of Herpes Zoster and Development of Post Herpetic Neuralgia: Review of International Publications. Med. Mal. Infect. 2012, 42, 53–58. 10.1016/j.medmal.2011.11.001. 22169279

[ref6] Sampathkumar P.; Drage L. A.; Martin D. P. Herpes Zoster (Shingles) and Postherpetic Neuralgia. Mayo Clin. Proc. 2009, 84, 274–280. 10.4065/84.3.274. 19252116 PMC2664599

[ref7] Field H. J.; Hodge R. A. V. Recent Developments in Anti-herpesvirus Drugs. Br. Med. Bull. 2013, 106, 213–249. 10.1093/bmb/ldt011. 23596085

[ref8] Andrei G.; Snoeck R. Advances and Perspectives in the Management of Varicella-Zoster Virus Infections. Molecules 2021, 26, 1132 10.3390/molecules26041132. 33672709 PMC7924330

[ref9] Patil A.; Goldust M.; Wollina U. Herpes zoster: A Review of Clinical Manifestations and Management. Viruses 2022, 14, 192 10.3390/v14020192. 35215786 PMC8876683

[ref10] Lee M. Y.; Kim K. S.; Lee W. K. Intravitreal Foscarnet for the Treatment of Acyclovir-resistant Acute Retinal Necrosis Caused by Varicella Zoster Virus. Ocul. Immunol. Inflammation 2011, 19, 212–213. 10.3109/09273948.2010.544857. 21595539

[ref11] Hoffman J. Overview of Antiviral Medications Used in Ophthalmology. Community Eye Health 2020, 33, 85–88. 32395035 PMC7205172

[ref12] Clercq E. D. (E)-5-(2-bromovinyl)-2′-deoxyuridine (BVDU). Med. Res. Rev. 2005, 25, 1–20. 10.1002/med.20011. 15389733

[ref13] Clercq E. D. Discovery and Development of BVDU (brivudin) as a Therapeutic for the Treatment of Herpes zoster. Biochem. Pharmacol. 2004, 68, 2301–2315. 10.1016/j.bcp.2004.07.039. 15548377

[ref14] Keizer H. J.; Debruijn E. A.; Tjaden U. R.; Declercq E. Inhibition of Fluorouracil Catabolism in Cancer-Patients by the Antiviral Agent (E)-5-(2-Bromovinyl)-2′-Deoxyuridine. J. Cancer Res. Clin. Oncol. 1994, 120, 545–549. 10.1007/BF01221032. 8045919 PMC12200904

[ref15] Choi Y.; Li L.; Grill S.; Gullen E.; Lee C. S.; Gumina G.; Tsujii E.; Cheng Y. C.; Chu C. K. Structure-Activity Relationships of (E)-5-(2-bromovinyl) Uracil and Related Pyrimidine Nucleosides as Antiviral Agents for Herpes Viruses. J. Med. Chem. 2000, 43, 2538–2546. 10.1021/jm990543n. 10891113

[ref16] De C.; Liu D. M.; Zheng B.; Singh U. S.; Chavre S.; White C.; Arnold R. D.; Hagen F. K.; Chu C. K.; Moffat J. F. beta-L-1-[5-(E-2-bromovinyl)-2-(hydroxymethyl)-1,3-(dioxolan-4-yl)] uracil (L-BHDU) Prevents Varicella-Zoster Virus Replication in a SCID-Hu Mouse Model and Does not Interfere with 5-Fluorouracil Catabolism. Antiviral Res. 2014, 110, 10–19. 10.1016/j.antiviral.2014.07.007. 25051026 PMC4171207

[ref17] Krečmerová M. Amino Acid Ester Prodrugs of Nucleoside and Nucleotide Antivirals. Mini. Rev. Med. Chem. 2017, 17, 818–833. 10.2174/1389557517666170216151601. 28215138

[ref18] Coen N.; Singh U.; Vuyyuru V.; Van den Oord J. J.; Balzarini J.; Duraffour S.; Snoeck R.; Cheng Y. C.; Chu C. K.; Andrei G. Activity and Mechanism of Action of HDVD, a Novel Pyrimidine Nucleoside Derivative with High Levels of Selectivity and Potency Against Gammaherpesviruses. J. Virol. 2013, 87, 3839–3851. 10.1128/JVI.03338-12. 23345517 PMC3624198

[ref19] Peterson L. W.; McKenna C. E. Prodrug Approaches to Improving the Oral Absorption of Antiviral Nucleotide Analogues. Expert Opin. Drug Delivery 2009, 6, 405–420. 10.1517/17425240902824808. PMC5117106 19382883

[ref20] Hostetler K. Y. Alkoxyalkyl Prodrugs of Acyclic Nucleoside Phosphonates Enhance Oral Antiviral Activity and Reduce Toxicity: Current State of the Art. Antiviral Res. 2009, 82, A84–A98. 10.1016/j.antiviral.2009.01.005. 19425198 PMC2768545

[ref21] Beadle J. R.; Hartline C.; Aldern K. A.; Rodriguez N.; Harden E.; Kern E. R.; Hostetler K. Y. Alkoxyalkyl Esters of Cidofovir and Cyclic Cidofovir Exhibit Multiple-log Enhancement of Antiviral Activity Against Cytomegalovirus and Herpesvirus Replication in vitro. Antimicrob. Agents Chemother. 2002, 46, 2381–2386. 10.1128/AAC.46.8.2381-2386.2002. 12121908 PMC127379

[ref22] Naesens L.; Neyts J.; Balzarini J.; Bischofberger N.; Declercq E. In-Vivo Antiretroviral Efficacy of Oral Bis(Pom)-Pmea, the Bis(Pivaloyloxymethyl)Prodrug of 9-(2-Phosphonylmethoxyethyl)Adenine (Pmea). Nucleosides, Nucleotides Nucleic Acids 1995, 14, 767–770. 10.1080/15257779508012468.

[ref23] Marcellin P.; Chang T.; Lim S. G.; et al. Adefovir dipivoxil for the Treatment of Hepatitis B e Antigen-Positive Chronic Hepatitis B. N. Engl. J. Med. 2003, 348, 808–816. 10.1056/NEJMoa020681. 12606735

[ref24] Fung H. B.; Stone E. A.; Piacenti F. J. Tenofovir Disoproxil Fumarate: A Nucleotide Reverse Transcriptase Inhibitor for the Treatment of HIV Infection. Clin. Ther. 2002, 24, 1515–1548. 10.1016/S0149-2918(02)80058-3. 12462284

[ref25] Pradere U.; Garnier-Amblard E. C.; Coats S. J.; Amblard F.; Schinazi R. F. Synthesis of Nucleoside Phosphate and Phosphonate Prodrugs. Chem. Rev. 2014, 114, 9154–9218. 10.1021/cr5002035. 25144792 PMC4173794

[ref26] Wiemer A. J.; Wiemer D. F. Prodrugs of Phosphonates and Phosphates: Crossing the Membrane Barrier. In Topics in Current Chemistry; Springer International Publishing, 2015; Vol. 360, pp 115–160. 25391982 10.1007/128_2014_561 PMC4774048

[ref27] Heidel K. M.; Dowd C. S. Phosphonate Prodrugs: An Overview and Recent Advances. Future Med. Chem. 2019, 11, 1625–1643. 10.4155/fmc-2018-0591. 31469328 PMC6722485

[ref28] McGuigan C.; Tsang H. W.; Sutton P. W.; De Clercq E.; Balzarini J. Synthesis and Anti-HIV Activity of Some Novel Chain-Extended Phosphoramidate Derivatives of d4T (Stavudine): Esterase Hydrolysis as a Rapid Predictive Test for Antiviral Potency. Antiviral Chem. Chemother. 1998, 9, 109–115. 10.1177/095632029800900202. 9875382

[ref29] Singh U. S.; Mulamoottil V. A.; Chu C. K. Synthesis of an Anti-hepatitis B Agent, 2′-Fluoro-6′-methylene-carbocyclic Adenosine (FMCA) and Its Phosphoramidate (FMCAP). J. Org. Chem. 2019, 84, 752–759. 10.1021/acs.joc.8b02599. 30589264

[ref30] Liang Y.; Narayanasamy J.; Schinazi R. F.; Chu C. K. Phosphoramidate and Phosphate Prodrugs of (-)-beta-D-(2R,4R)-dioxolane-thymine: Synthesis, Anti-HIV Activity and Stability Studies. Bioorg. Med. Chem. 2006, 14, 2178–2189. 10.1016/j.bmc.2005.11.008. 16314108

[ref31] Hwang Y. S.; Cole P. A. Efficient Synthesis of Phosphorylated Prodrugs with Bis(POM)-phosphoryl Chloride. Org. Lett. 2004, 6, 1555–1556. 10.1021/ol049714v. 15128234

[ref32] Oliver S. L.; Zerboni L.; Sommer M.; Rajamani J.; Arvin A. M. Development of Recombinant Varicella-Zoster Viruses Expressing Luciferase Fusion Proteins for Live in vivo Imaging in Human Skin and Dorsal Root Ganglia Xenografts. J. Virol. Methods 2008, 154, 182–193. 10.1016/j.jviromet.2008.07.033. 18761377 PMC2657092

[ref33] De C.; Liu D.; Singh U. S.; Chu C. K.; Moffat J. F. β-L-1-[5-(E-2-Bromovinyl)-2-(Hydroxymethyl)-1,3 Dioxolan-4-yl)] Uracil (L-BHDU) Inhibits Varicella Zoster Virus Replication by Depleting the Cellular dTTP Pool. bioRxiv 2020, 10.1101/2020.02.13.948216.

[ref34] Lloyd M. G.; Yee M. B.; Flot J. S.; Liu D. M.; Geiler B. W.; Kinchington P. R.; Moffat J. F. Development of Robust Varicella Zoster Virus Luciferase Reporter Viruses for In Vivo Monitoring of Virus Growth and Its Antiviral Inhibition in Culture, Skin, and Humanized Mice. Viruses 2022, 14, 826 10.3390/v14040826. 35458556 PMC9032946

[ref35] Lloyd M. G.; Liu D.; Lyu J.; Fan J.; Overhulse J. M.; Kashemirov B. A.; Prichard M. N.; McKenna C. E.; Moffat J. F. An Acyclic Phosphonate Prodrug of HPMPC is Effective Against VZV in Skin Organ Culture and Mice. Antiviral Res. 2022, 199, 105275 10.1016/j.antiviral.2022.105275. 35248614 PMC12911940

[ref36] Lloyd M. G.; Liu D.; Legendre M.; Markovitz D. M.; Moffat J. F. H84T BanLec has Broad Spectrum Antiviral Activity Against Human Herpesviruses in Cells, Skin, and Mice. Sci. Rep. 2022, 12, 1641 10.1038/s41598-022-05580-6. 35102178 PMC8803833

[ref37] Rowe J.; Greenblatt R. J.; Liu D. M.; Moffat J. F. Compounds that Target Host Cell Proteins Prevent Varicella-Zoster Virus Replication in Culture, Ex vivo, and in SCID-Hu Mice. Antiviral Res. 2010, 86, 276–285. 10.1016/j.antiviral.2010.03.007. 20307580 PMC2866756

[ref38] Lloyd M. G.; Smith N. A.; Tighe M.; Travis K. L.; Liu D.; Upadhyaya P. K.; Kinchington P. R.; Chan G. C.; Moffat J. F. A Novel Human Skin Tissue Model To Study Varicella-Zoster Virus and Human Cytomegalovirus. J. Virol. 2020, 94, e01082-20 10.1128/JVI.01082-20. 32878893 PMC7592229

