# Supplementary material for: Prodrug Strategies for the Development of β-l-5-((E)-2-Bromovinyl)-1-((2S,4S)-2-(hydroxymethyl)-1,3-(dioxolane-4-yl))uracil (l-BHDU) against Varicella Zoster Virus (VZV)
Source: J Med Chem. 2023 May 4;66(10):7038–53. doi: 10.1021/acs.jmedchem.3c00545 (PMC10226046; doi:10.1021/acs.jmedchem.3c00545)
Supplement: Supplementary file 1 — jm3c00545_si_001.pdf [file jm3c00545_si_001.pdf]

Supporting Information

Prodrug Strategies for the Development of β-L-5-((*E*)-2-bromovinyl)-1-((2S,4S)-2-(hydroxymethyl)-1,3-(dioxolane-4-yl)uracil (L-BHDU) Against Varicella Zoster Virus (VZV)

Uma S. Singh,*† Ananda K. Konreddy,† Yugandhar Kothapalli,† Dongmei Liu,‡ Megan G. Lloyd,‡ Vidya Annavarapu,† Catherine A. White,† Michael. G. Bartlett,† Jennifer F. Moffat,‡ Chung K. Chu*†

†Department of Pharmaceutical and Biomedical Science, College of Pharmacy, University of Georgia, Athens, Georgia 30602, United States

‡State University of New York, Upstate Medical University, Syracuse, NY 13210, United States

Corresponding author: Uma S. Singh, ussingh@uga.edu

Corresponding author: Chung K. Chu, dchu@uga.edu

Table of Contents

S.No	Description of data	Page number
1.	^{1}H & ^{13}C NMR of L-BHDU-amino acid ester prodrugs (**2-26**)	S2 - S26
2.	^{1}H, ^{13}C & ^{31}P NMR of L-BHDU-phosphoramidate prodrugs (**33-34**)	S27 - S29
3.	^{1}H, ^{13}C & ^{31}P NMR of ODE-L-BHDU-MP (**38**) and HDP-L-BHDU-MP (**39**) prodrugs	S30 - S32
4.	^{1}H, ^{13}C & ^{31}P NMR of POM-L-BHDU-MP (**41**) and POC-L-BHDU-MP (**47**) prodrugs	S33 - S35
5.	Elemental Analysis data for final compounds	S-36
6.	HPLC methods and trace for compounds **1**, **24**, **34**, **38**, **39**,**41** & **47**	S37-S43

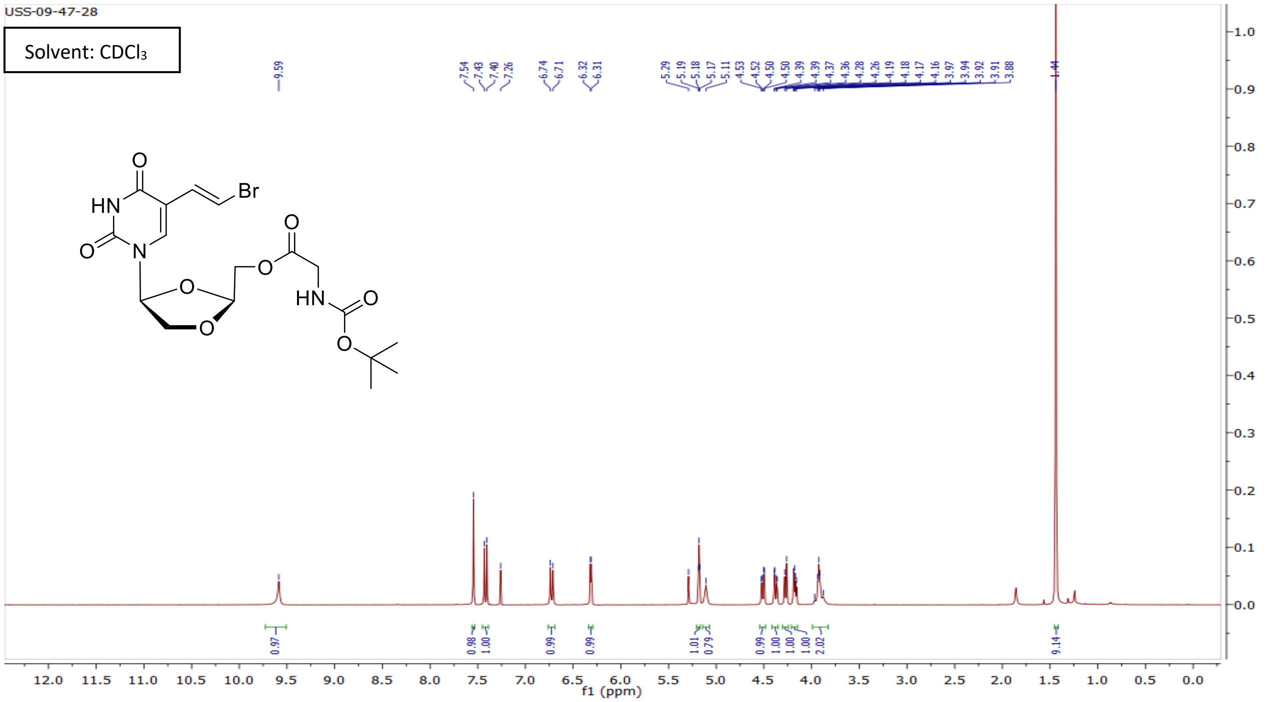

^{1}H NMR of L-BHDU-5'-*O*-L-Boc-glycine ester (**2**)

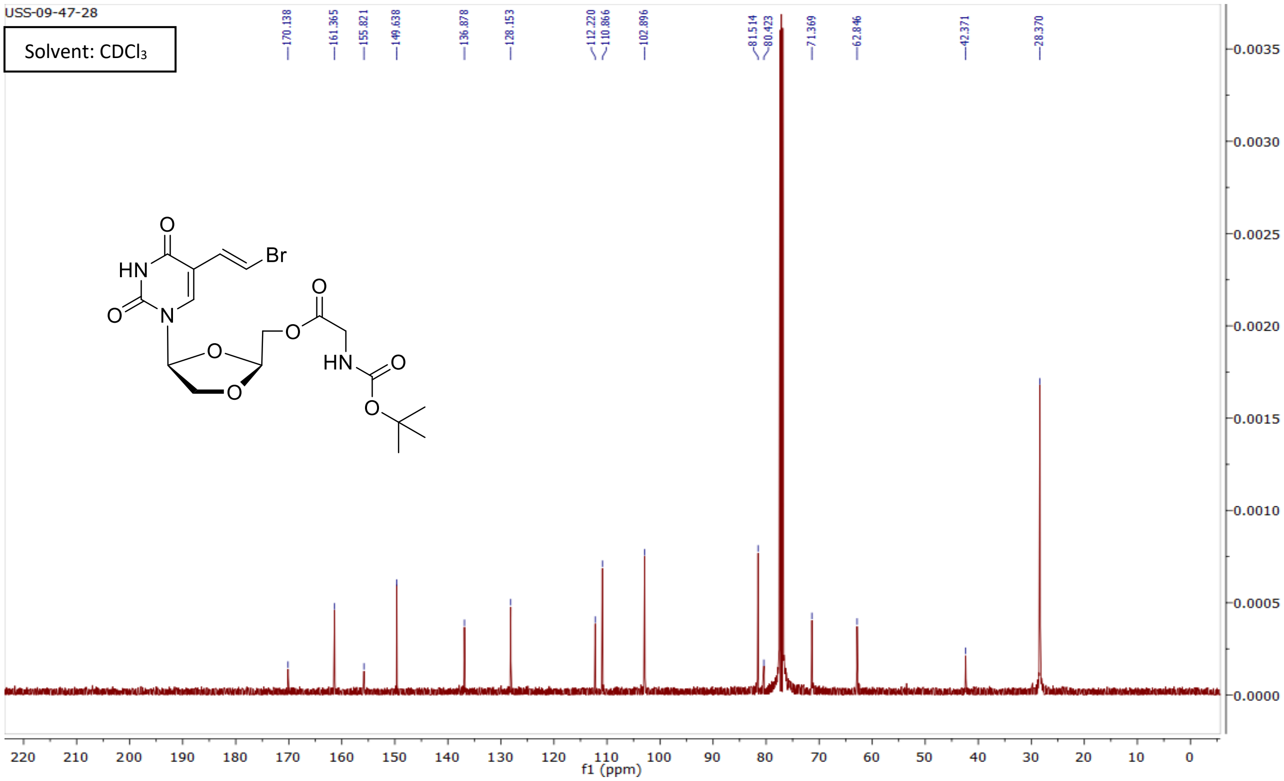

^{13}C NMR of L-BHDU-5'-*O*-*L*-Boc-glycine ester (**2**)

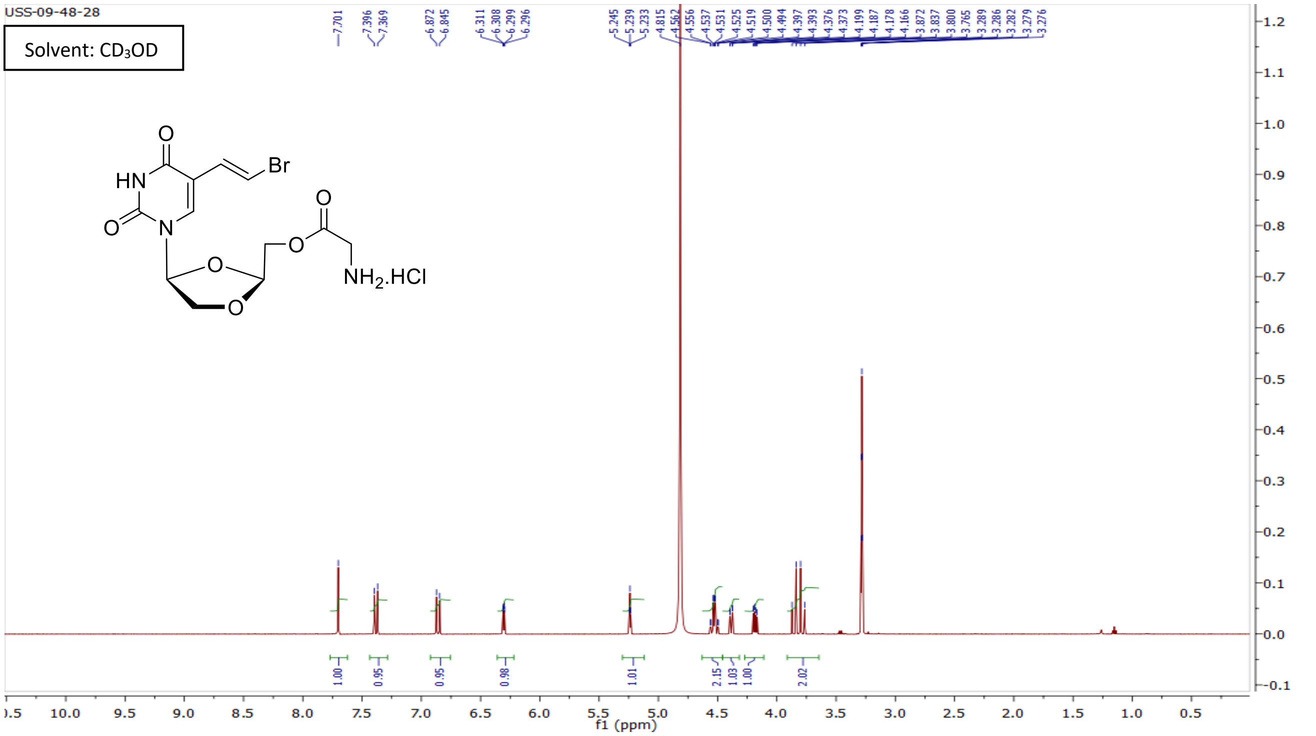

^{1}H NMR of L-BHDU-5′-*O*-glycyl ester hydrochloride (**14**)

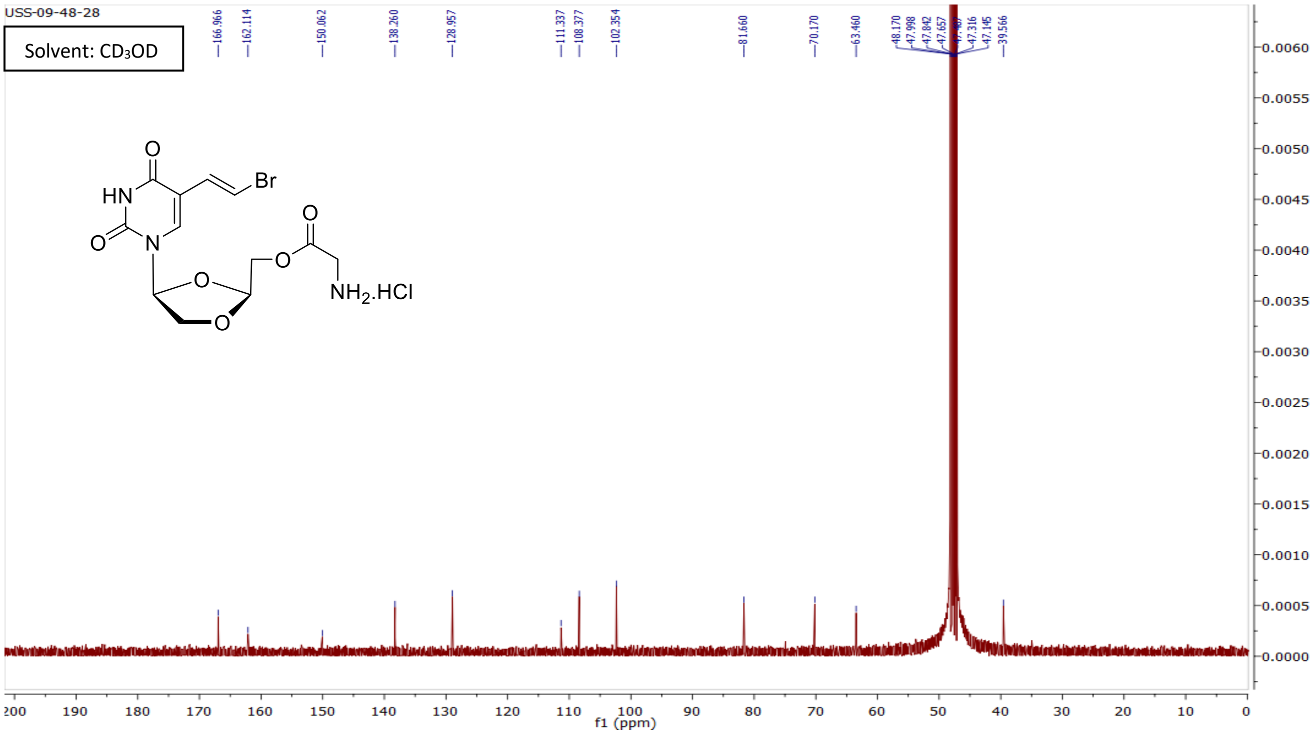

^{13}C NMR of L-BHDU-5′-*O*-glycyl ester hydrochloride (**14**)

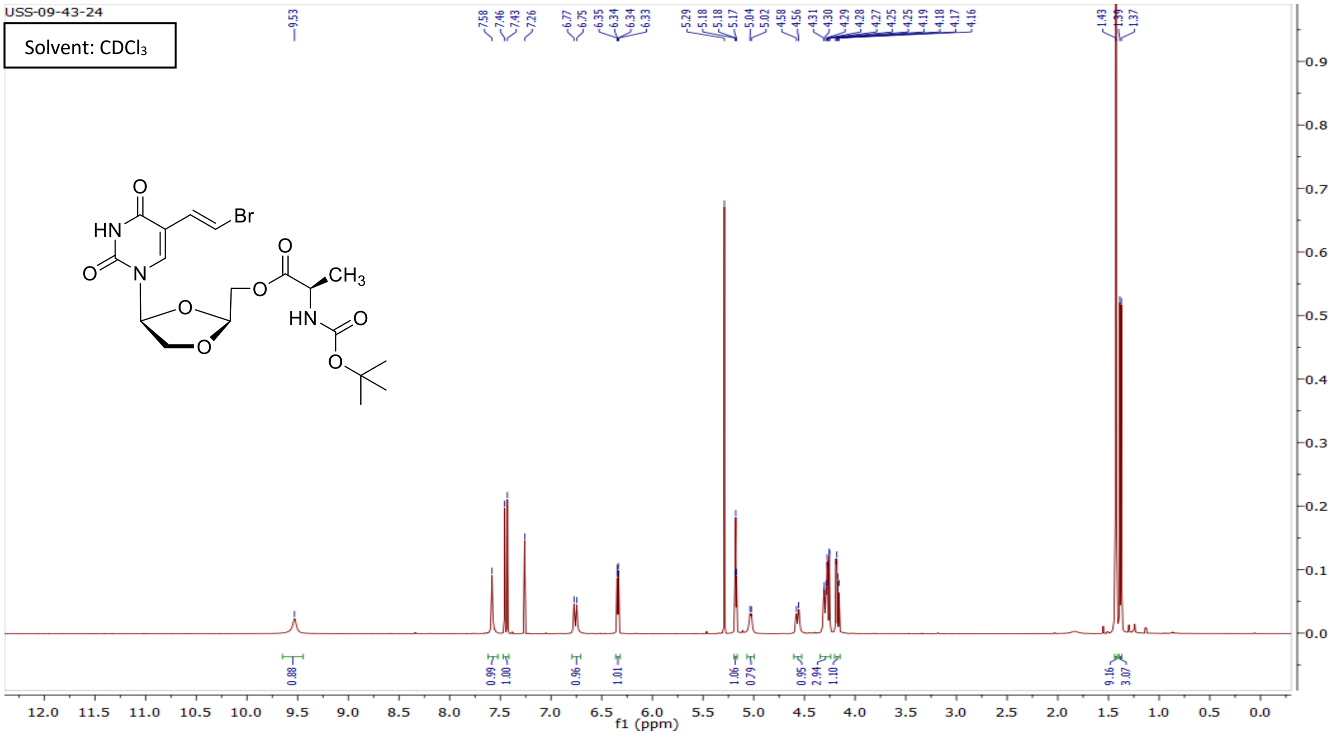

^{1}H NMR of L-BHDU-5'-*O*-L- Boc-alanine ester (**3**)

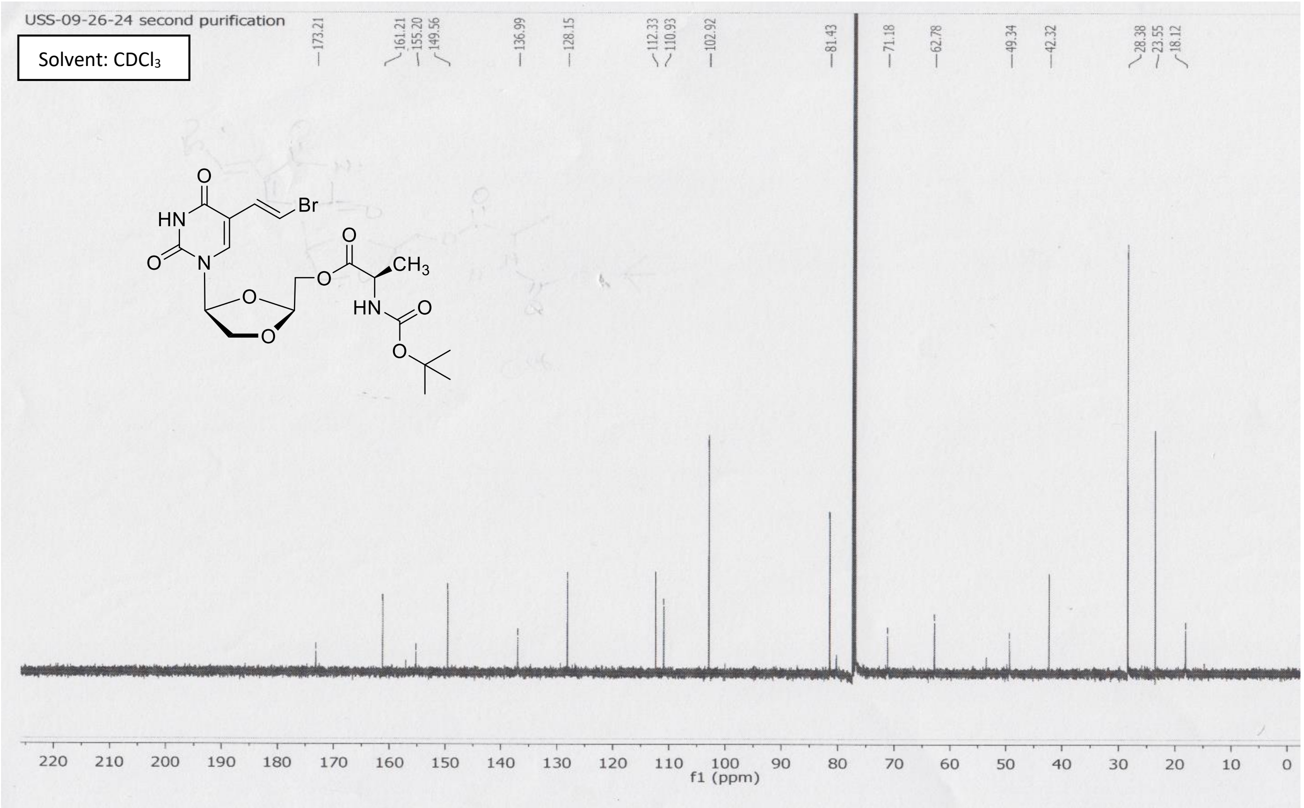

^{13}C NMR of L-BHDU-5'-*O*-L- Boc-alanine ester (**3**)

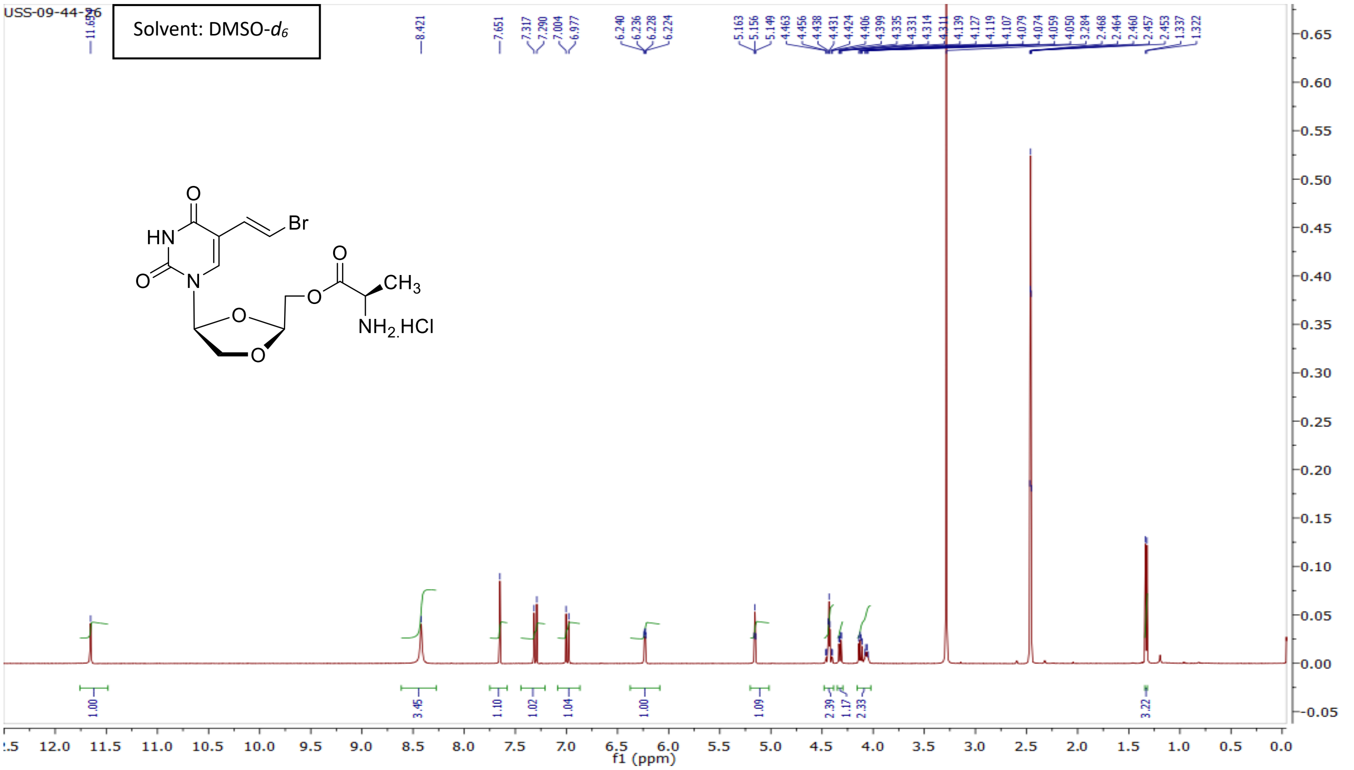

^{1}H NMR of L-BHDU-5′-*O*-L-alanyl ester hydrochloride (**15**)

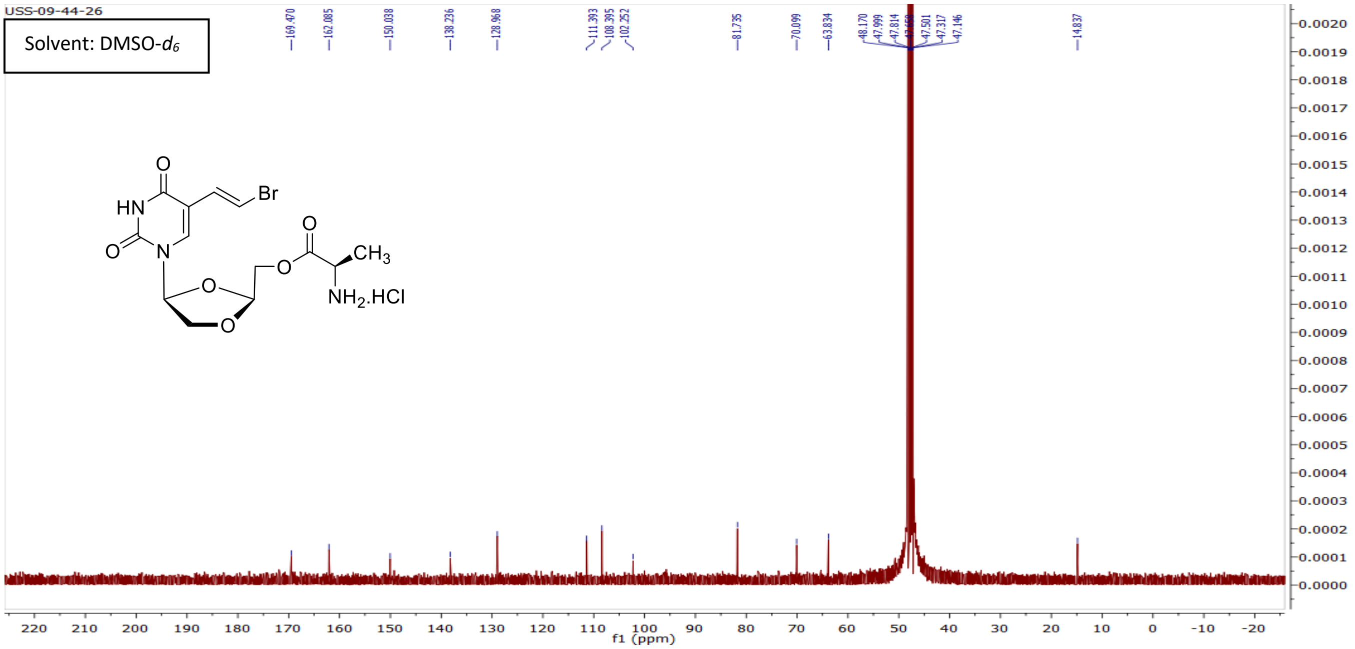

^{13}C NMR of L-BHDU-5′-*O*-L-alanyl ester hydrochloride (**15**)

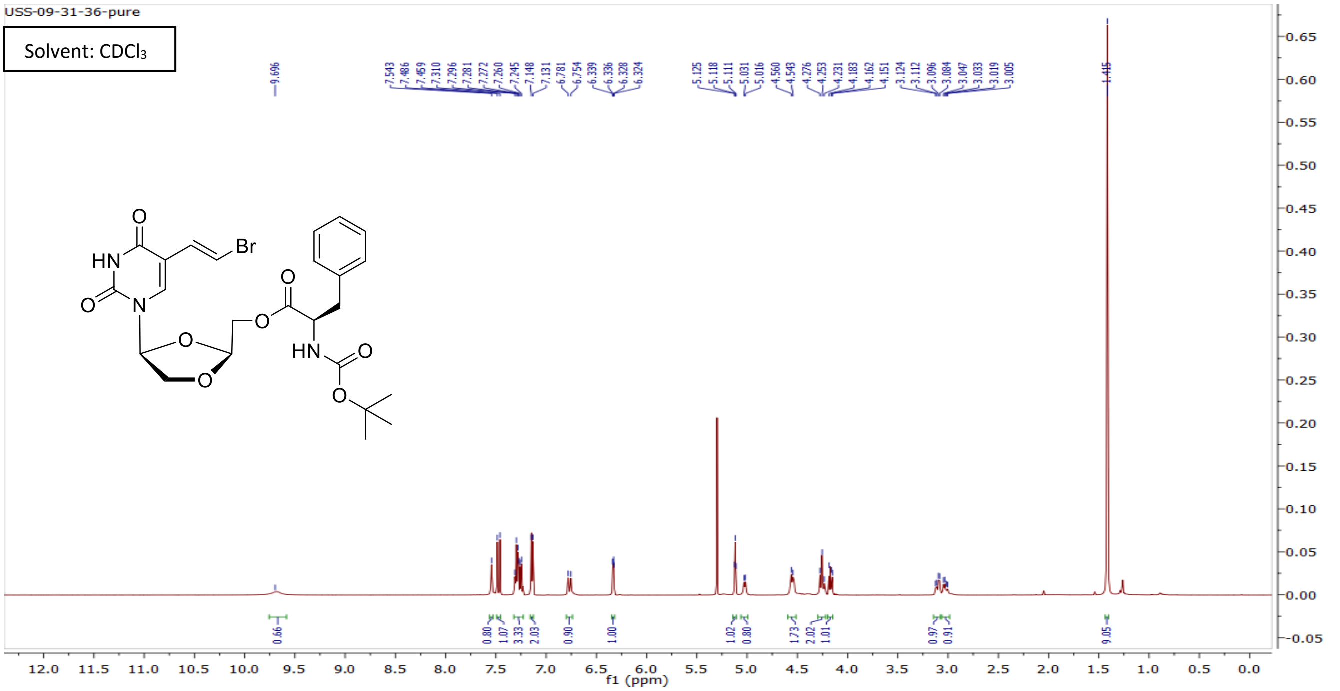

^{1}H NMR of L-BHDU-5'-*O*-L-Boc-phenylalanine ester (**4**)

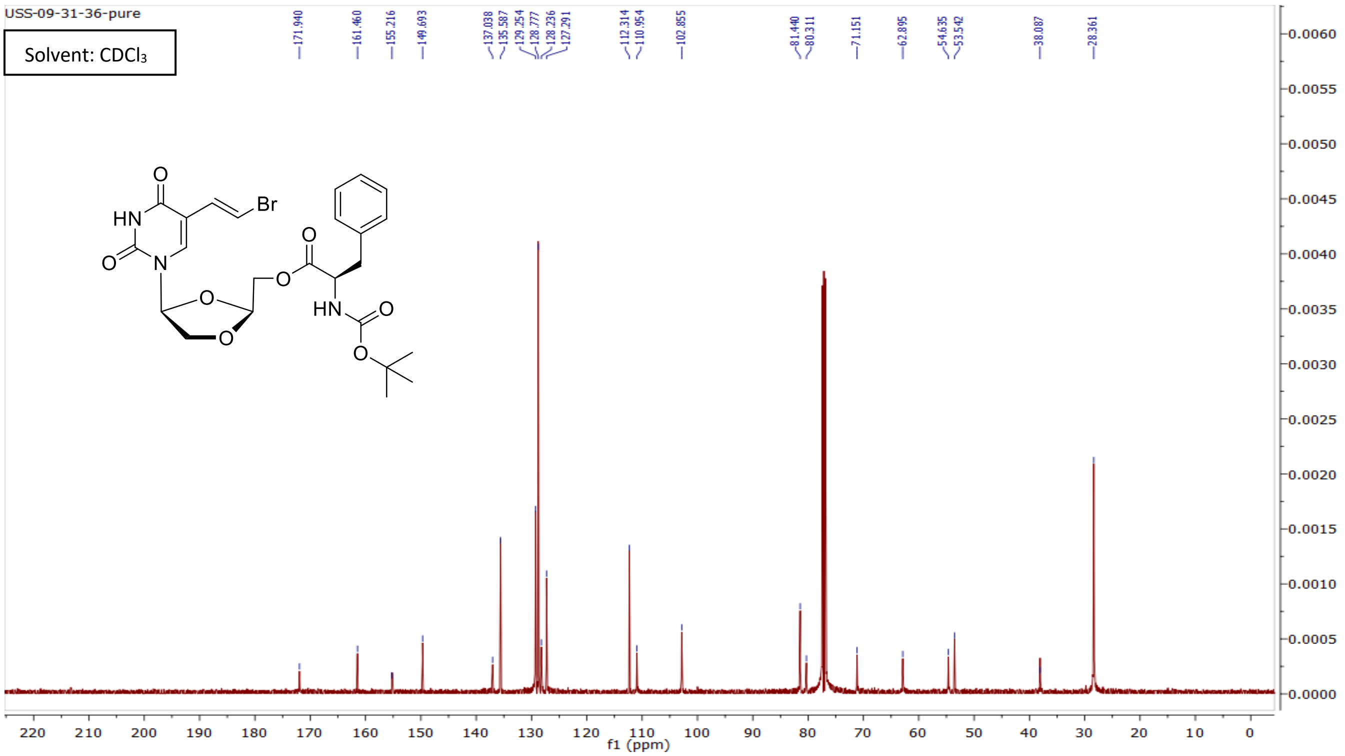

^{13}C NMR of L-BHDU-5'-*O*-L-Boc-phenylalanine ester (**4**)

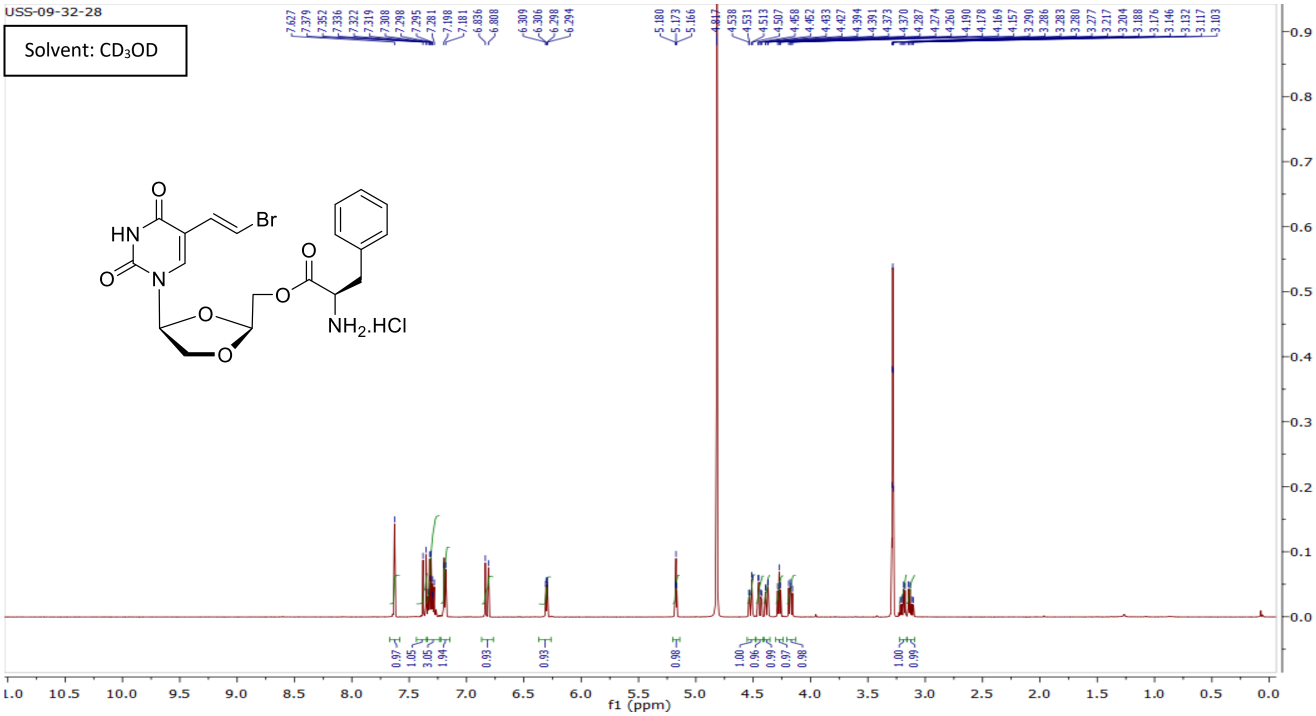

^{1}H NMR of L-BHDU-5′-*O*-L-phenylalanyl ester hydrochloride (**16**)

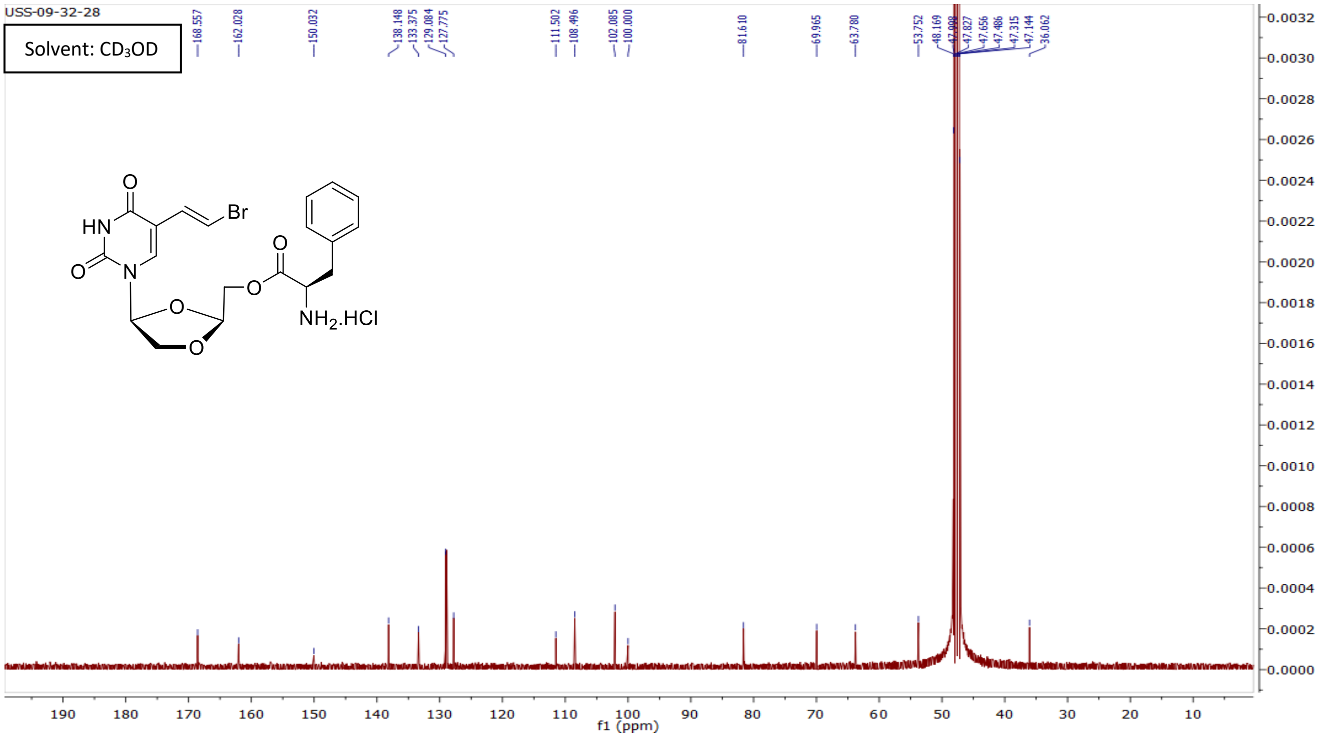

^{13}C NMR of L-BHDU-5′-*O*-L-phenylalanyl ester hydrochloride (**16**)

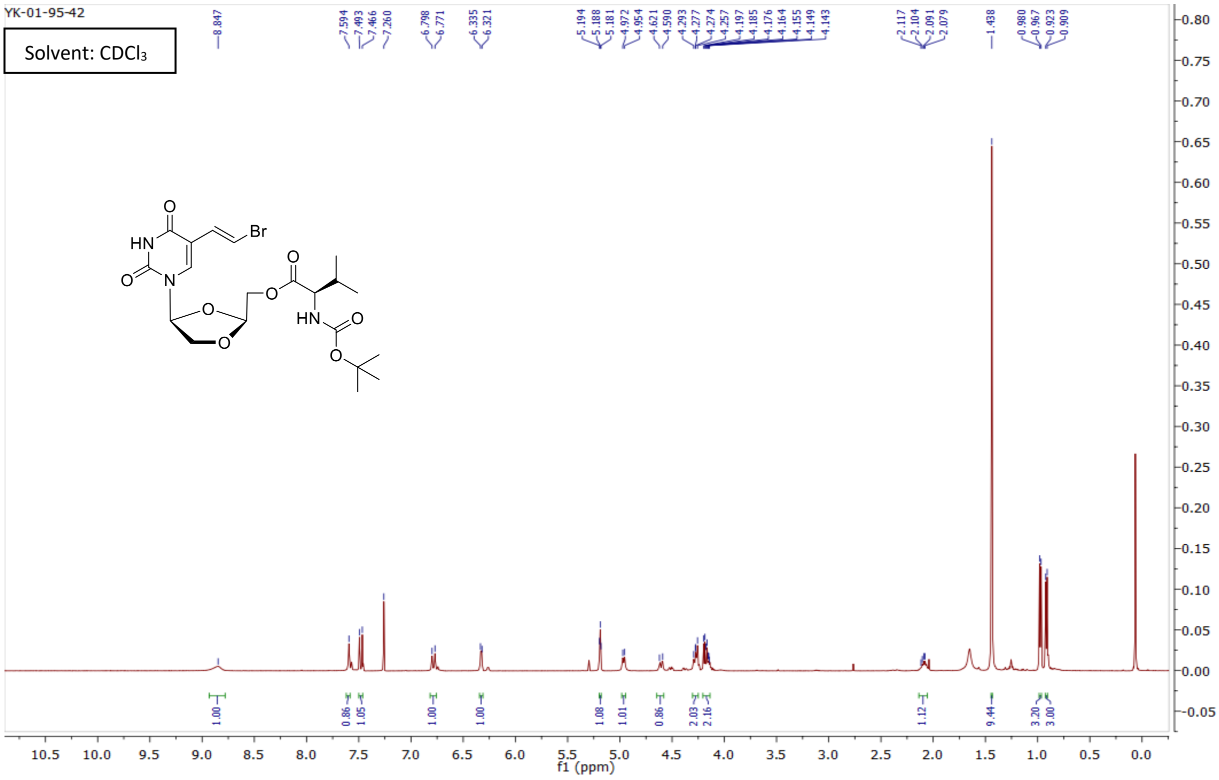

^{1}H NMR of L-BHDU-5'-*O*-L-Boc-valine ester **(5)**

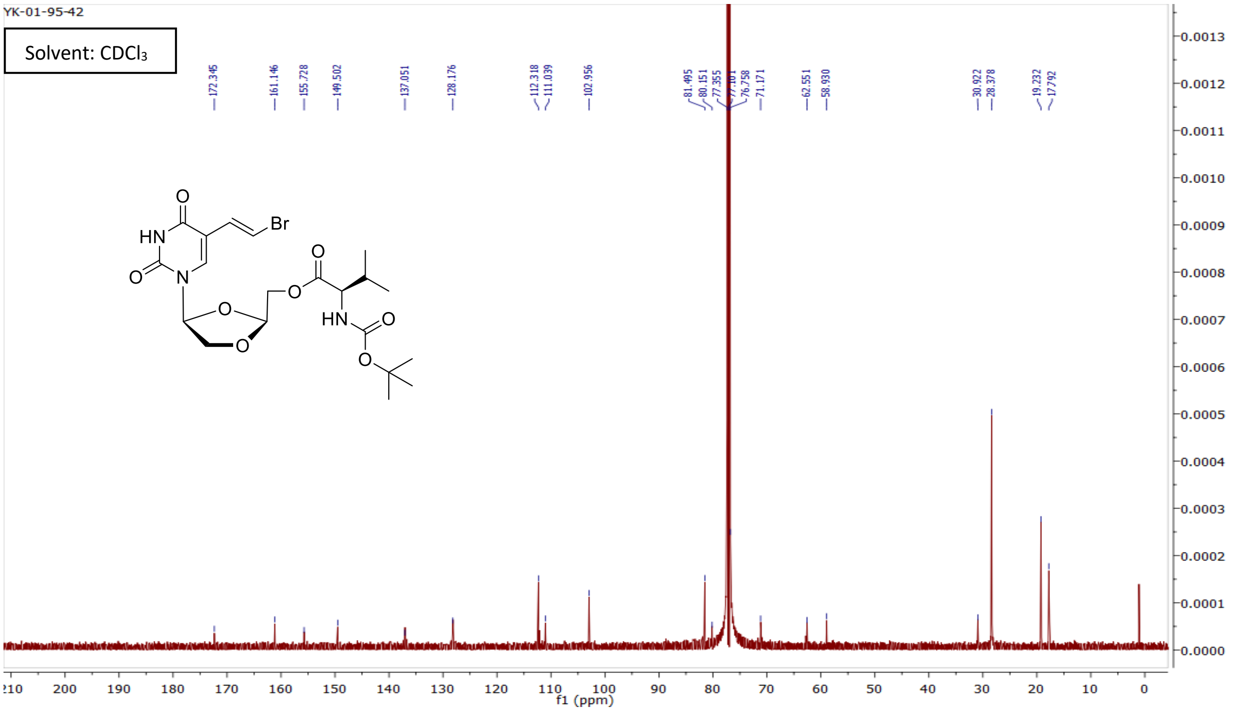

^{13}C NMR of L-BHDU-5'-*O*-L-Boc-valine ester **(5)**

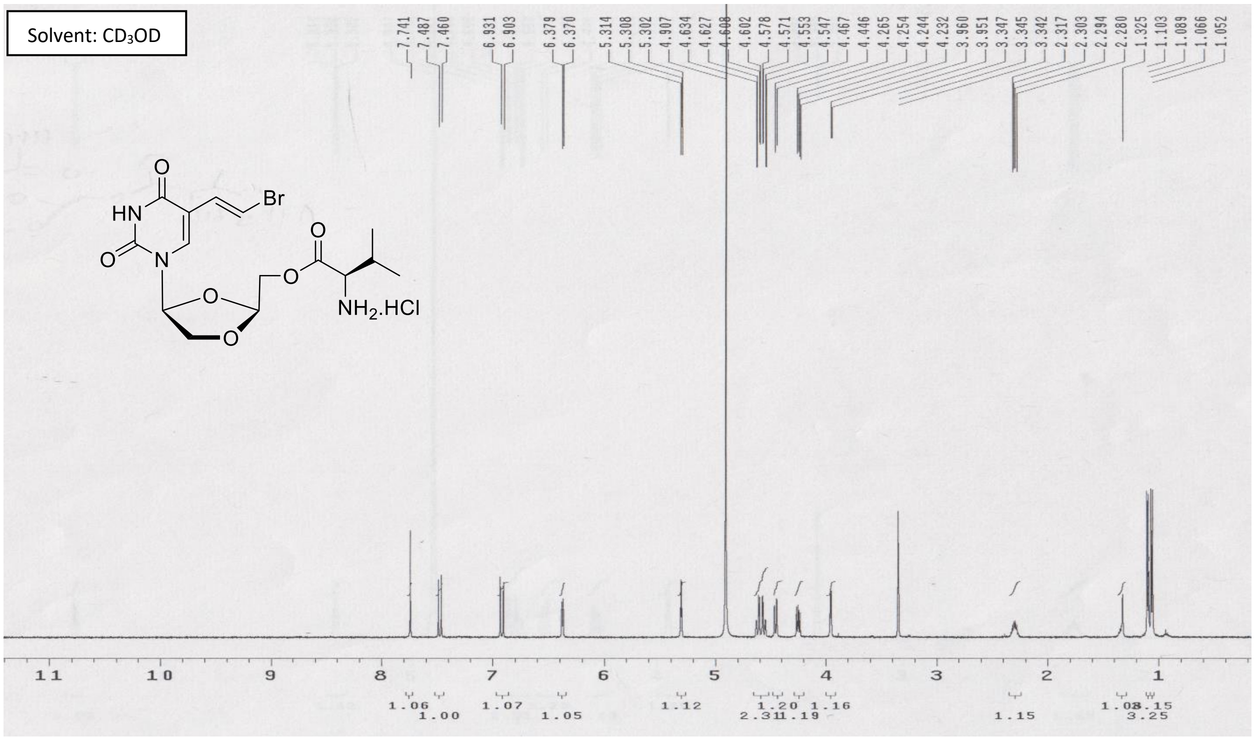

^{1}H NMR of L-BHDU-5′-*O*-L-valyl ester hydrochloride **(17)**

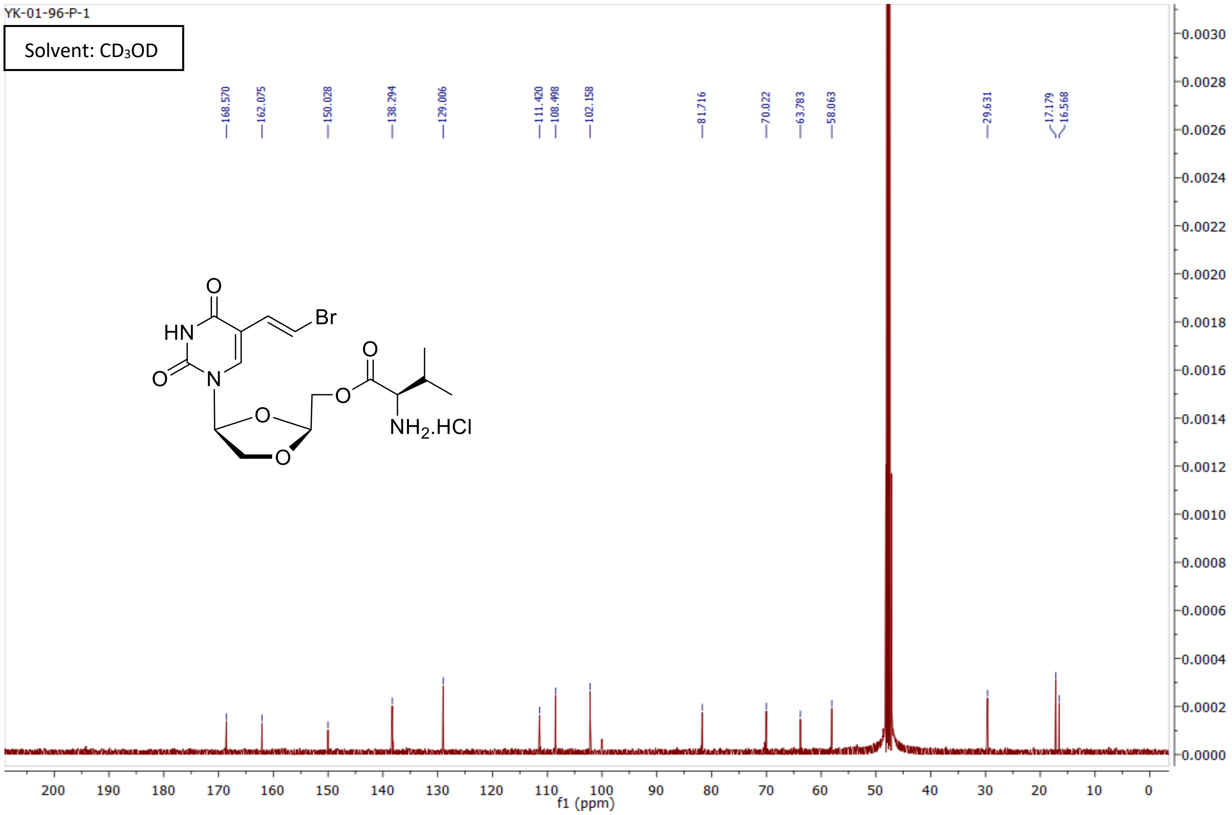

^{13}C NMR of L-BHDU-5′-O-L-valyl ester hydrochloride **(17)**

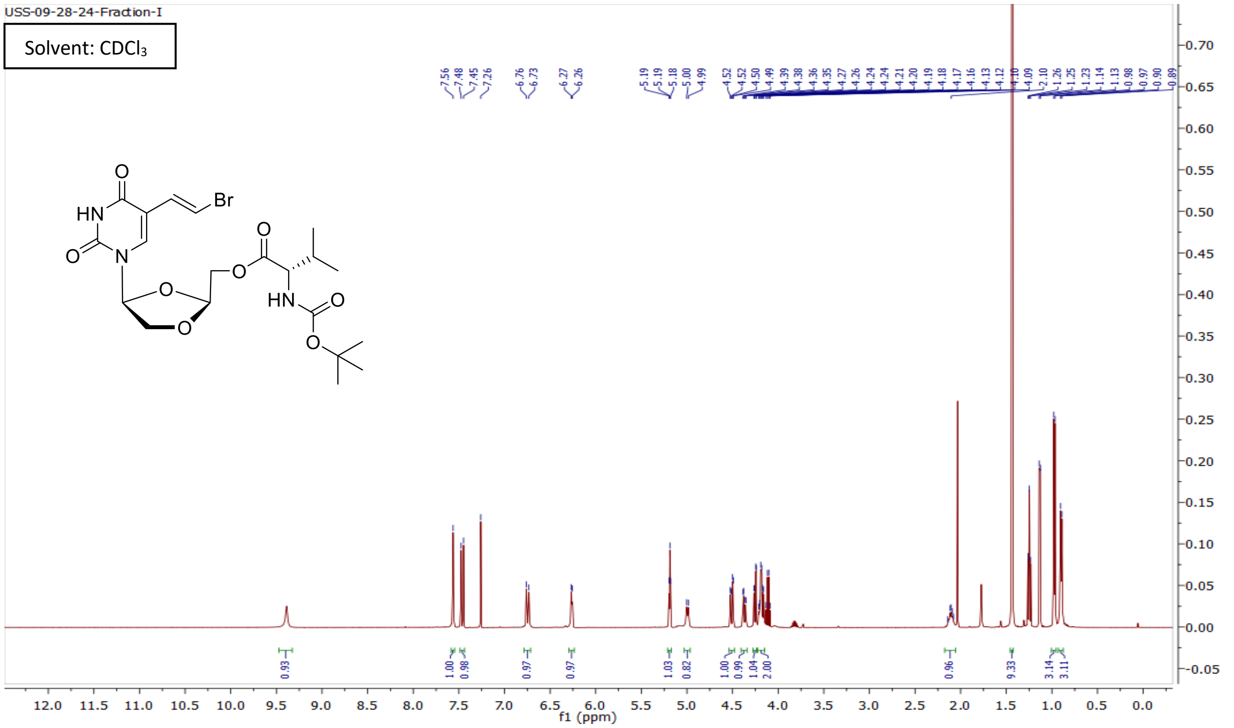

^{1}H NMR of L-BHDU-5'-*O*-D-Boc-valine ester **(6)**

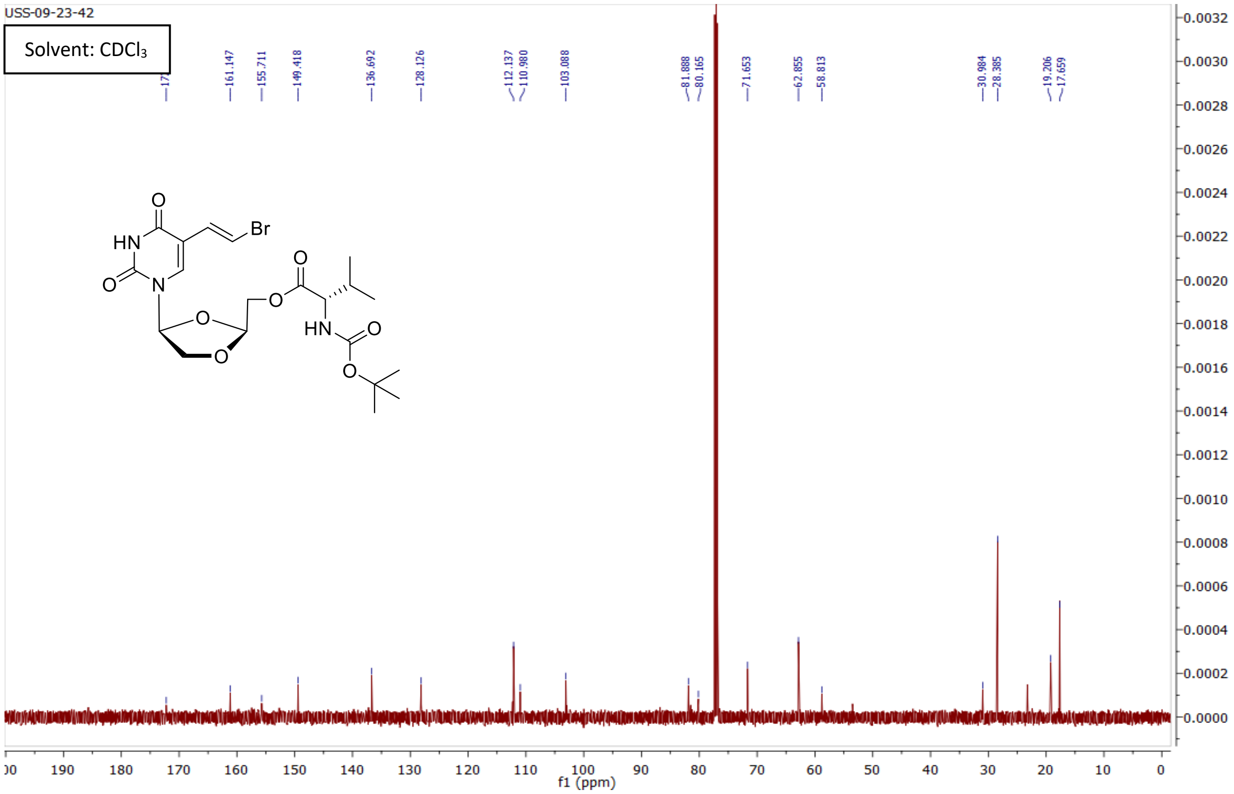

^{13}C NMR of L-BHDU-5'-*O*-D-Boc-valine ester **(6)**

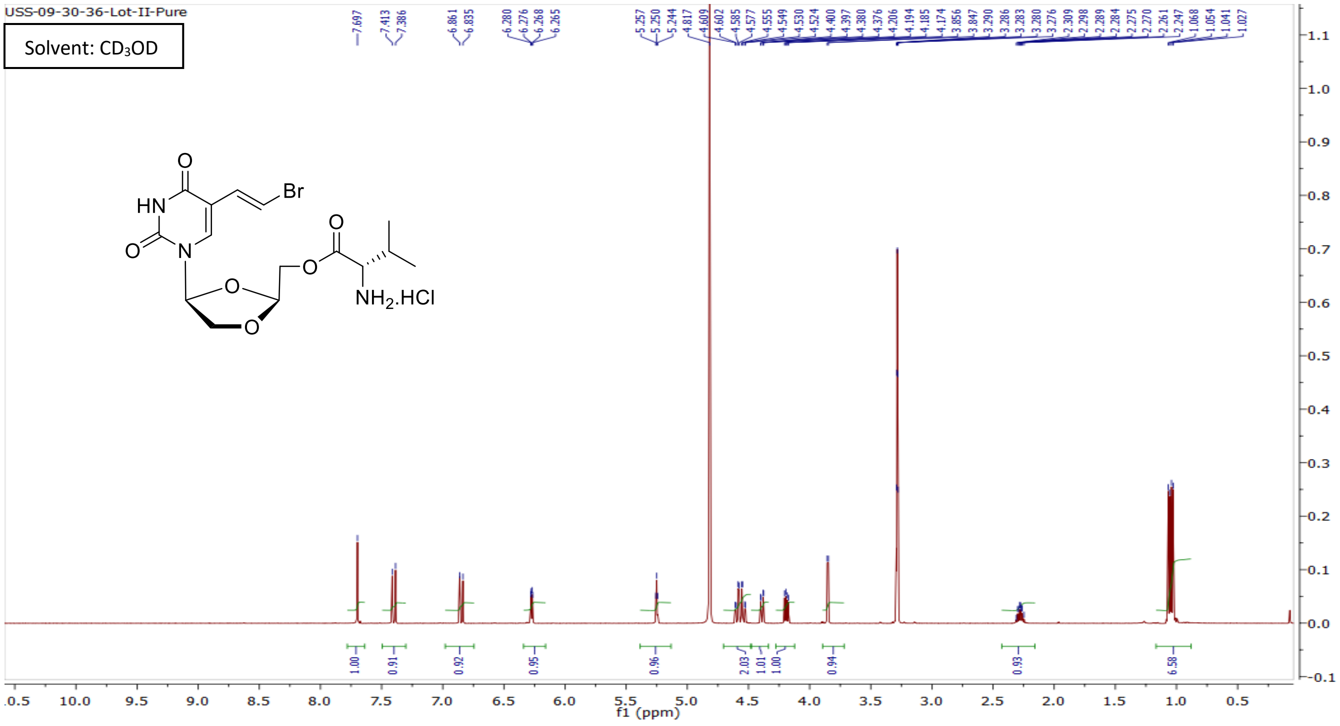

^{1}H NMR of L-BHDU-5′-*O*-D-valyl ester hydrochloride **(18)**

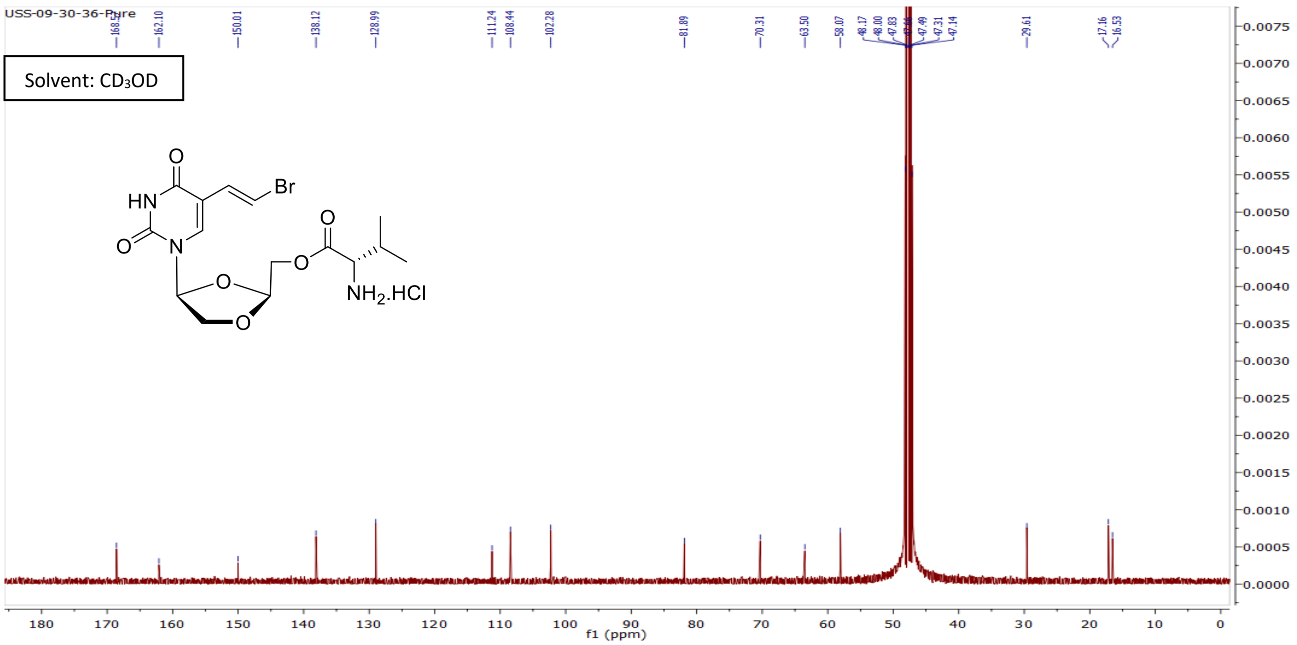

^{13}C NMR of L-BHDU-5′-*O*-D-valyl ester hydrochloride **(18)**

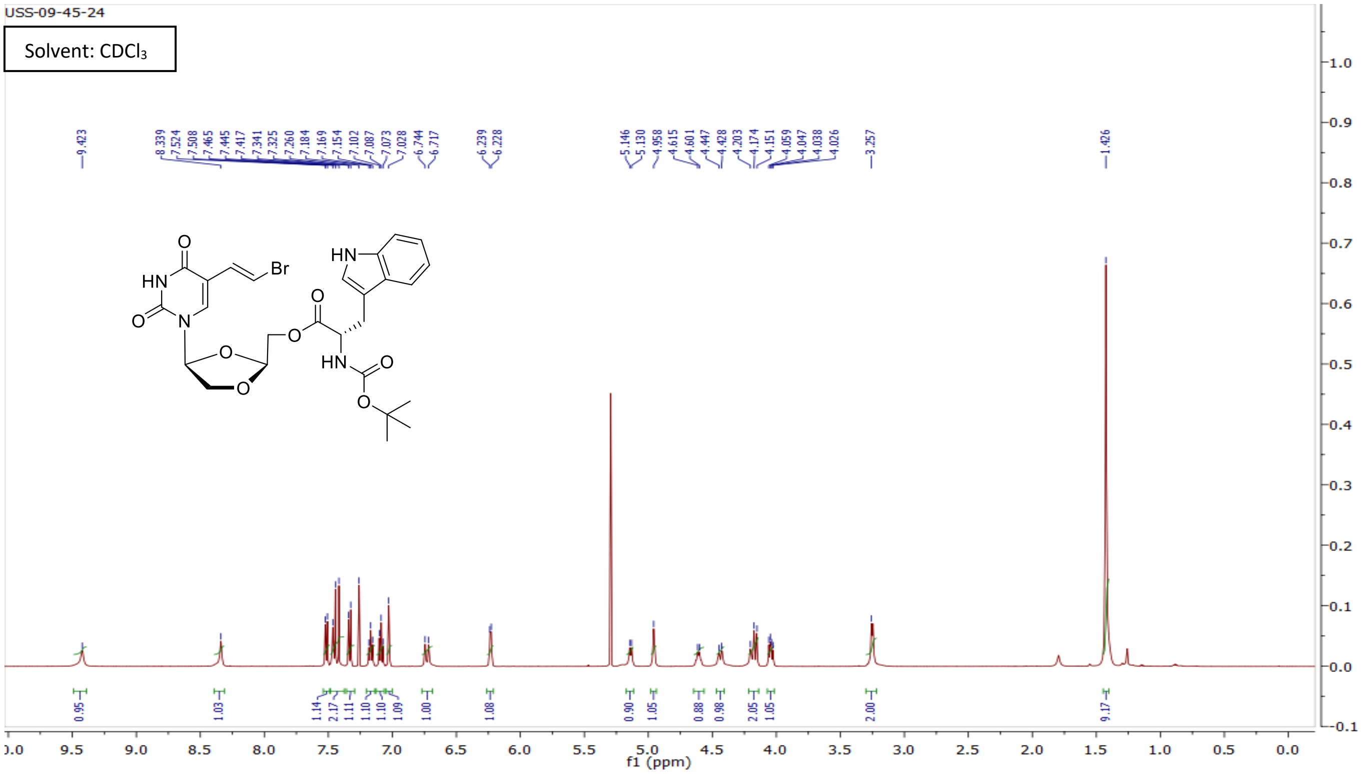

^{1}H NMR of L-BHDU-5'-*O*-L-Boc-tryptophan ester **(7)**

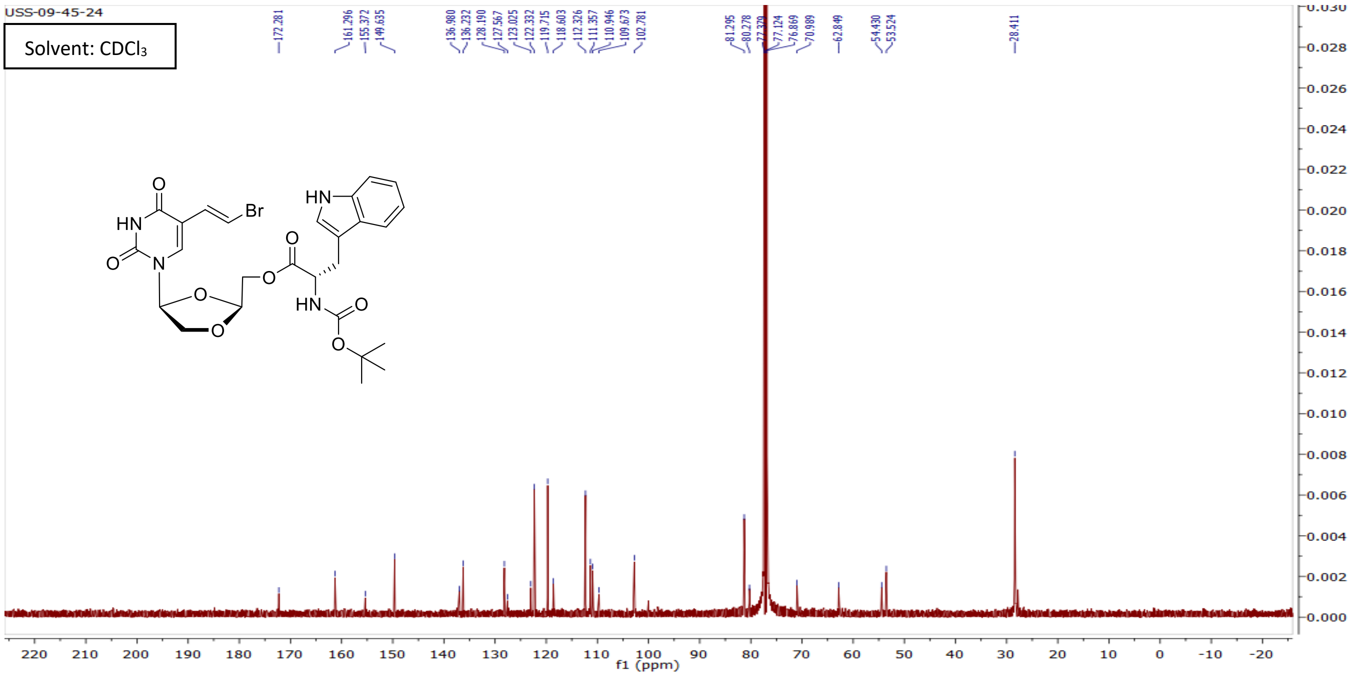

^{13}C NMR of L-BHDU-5'-*O*-L-Boc-tryptophan ester **(7)**

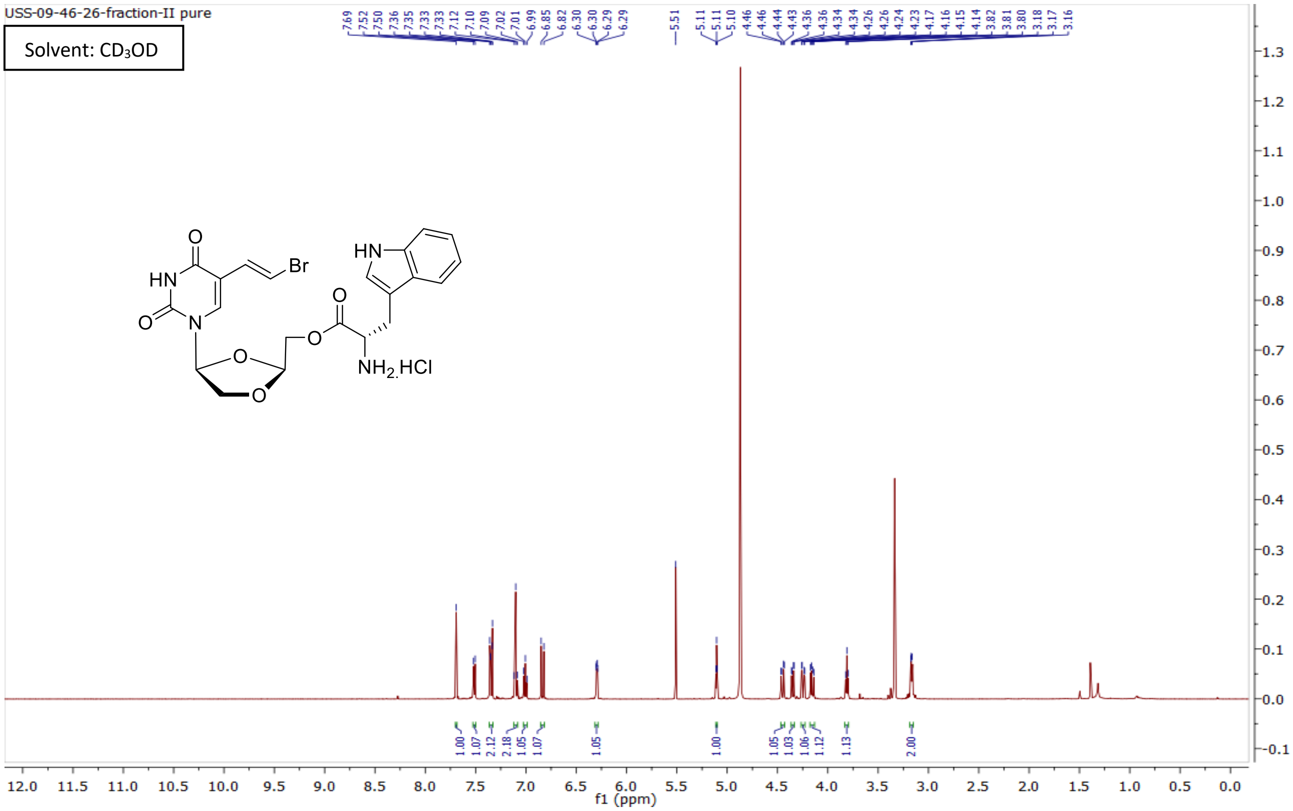

^{1}H NMR of L-BHDU-5′-*O*-L-tryptophan ester hydrochloride **(19)**

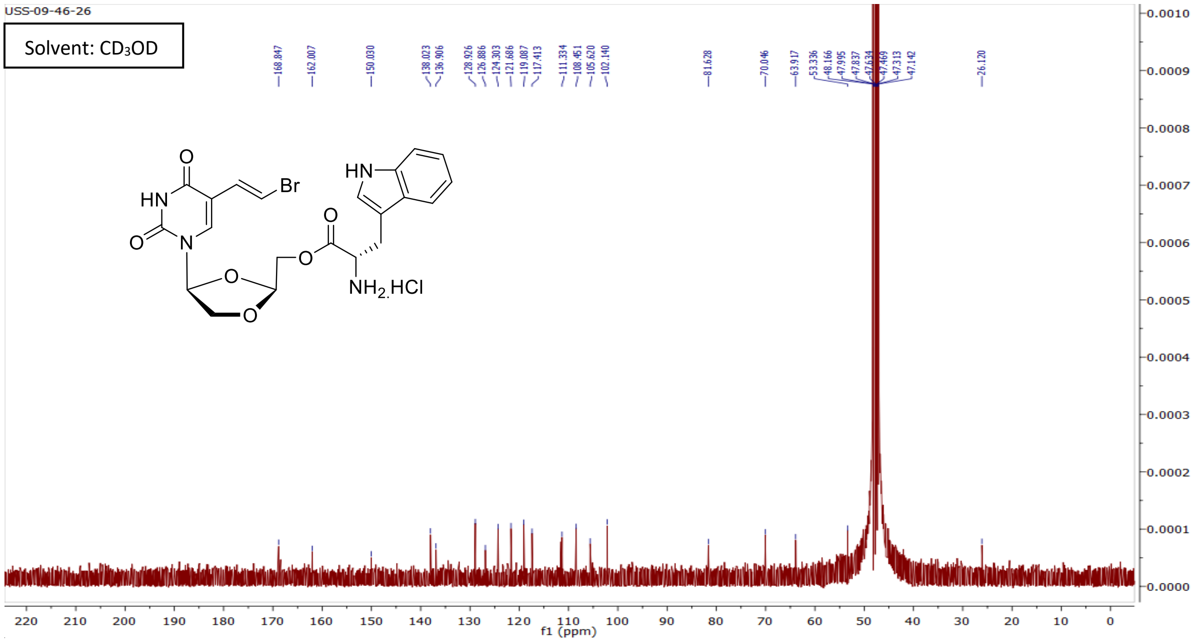

^{13}C NMR of L-BHDU-5′-*O*-L-tryptophan ester hydrochloride **(19)**

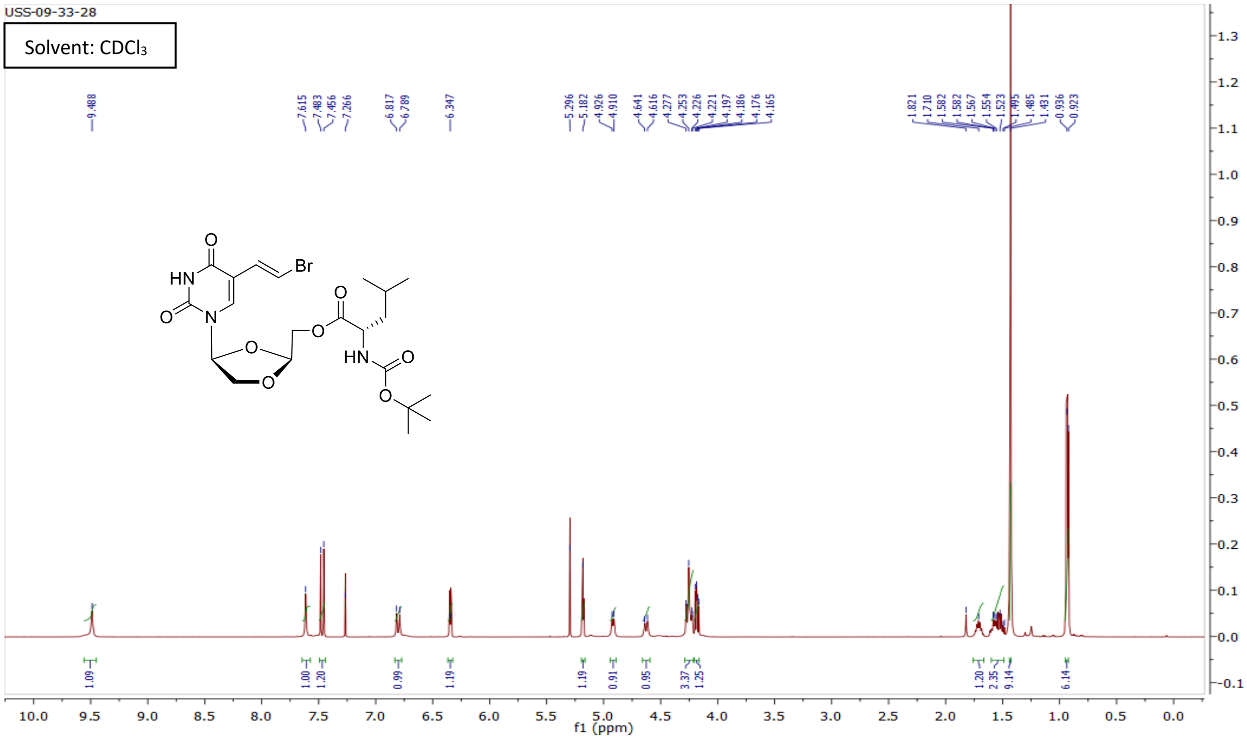

^{1}H NMR of L-BHDU-5'-*O*-L-Boc-leucine ester **(8)**

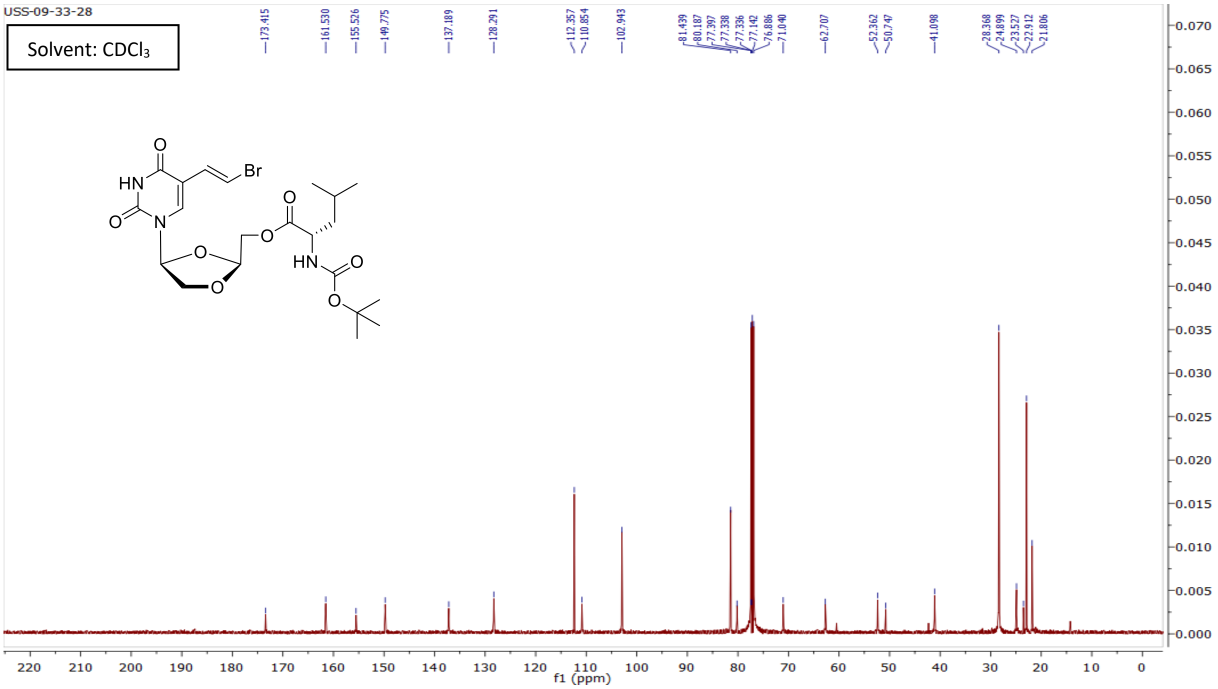

^{13}C NMR of L-BHDU-5'-*O*-L-Boc-leucine ester **(8)**

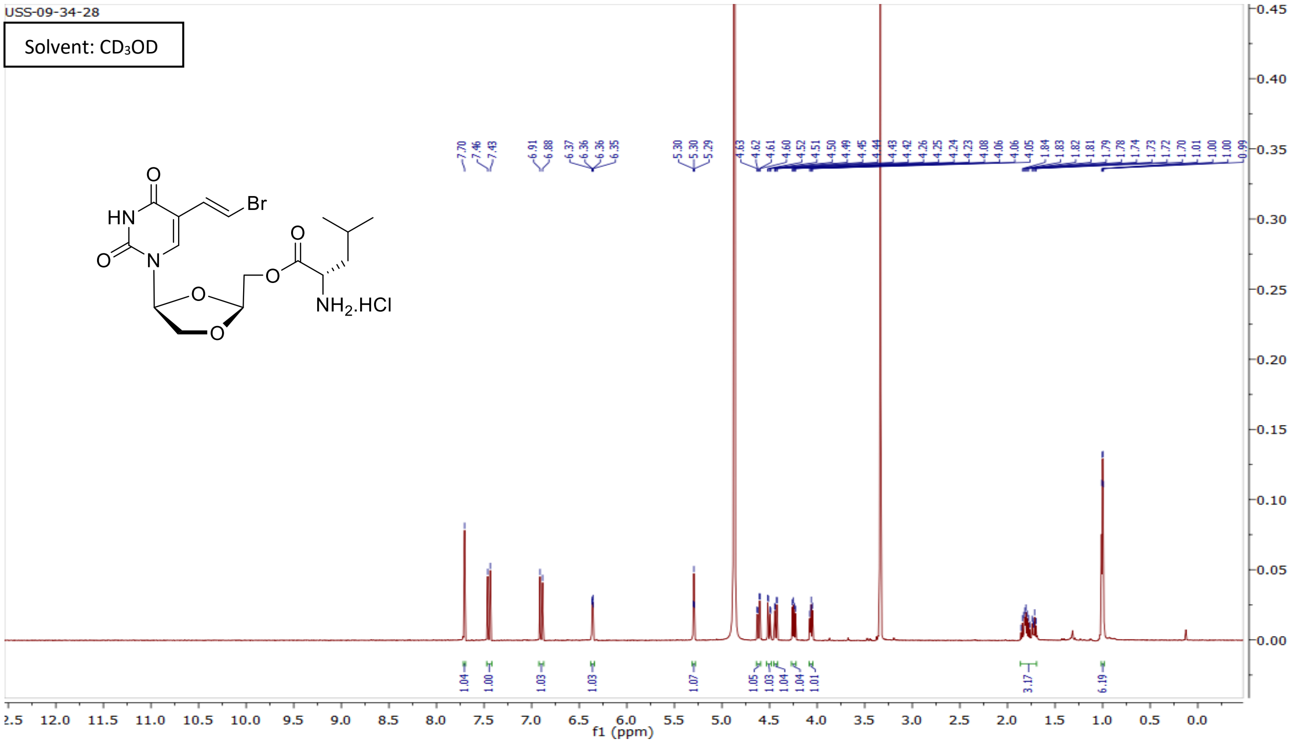

^{1}H NMR of L-BHDU-5'-*O*-L-leucine ester hydrochloride **(20)**

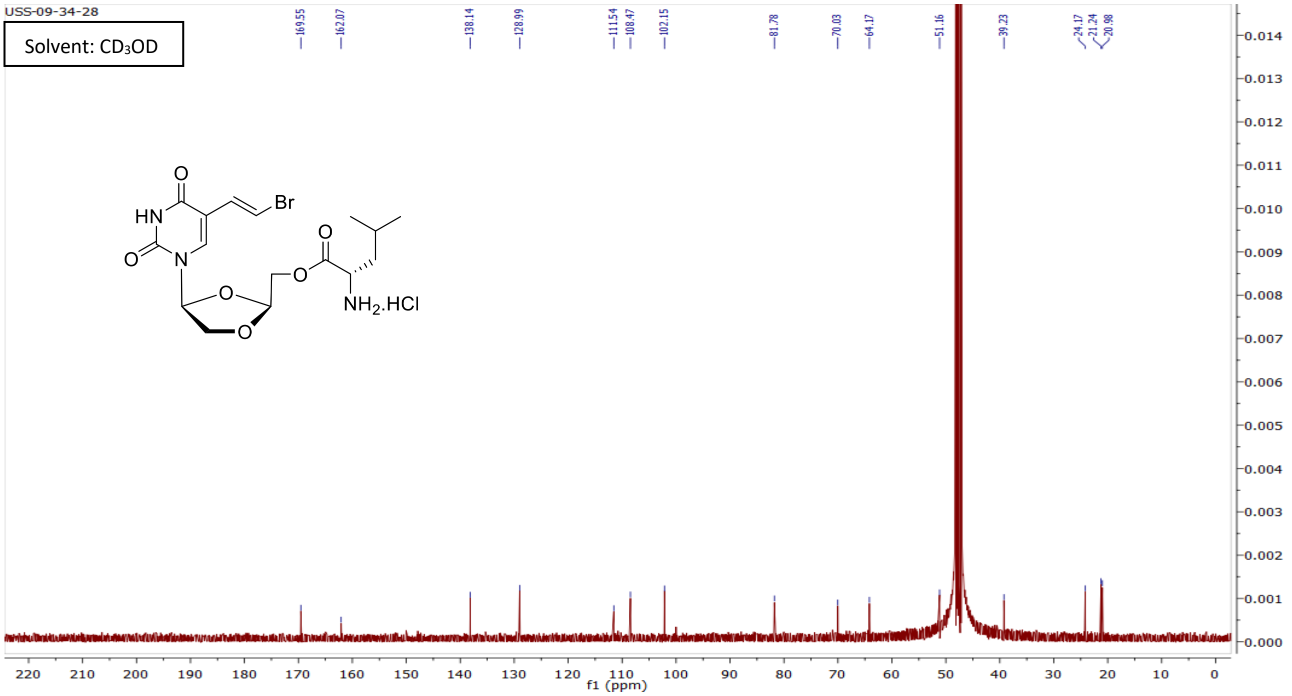

^{13}C NMR of L-BHDU-5'-*O*-L-leucine ester hydrochloride **(20)**

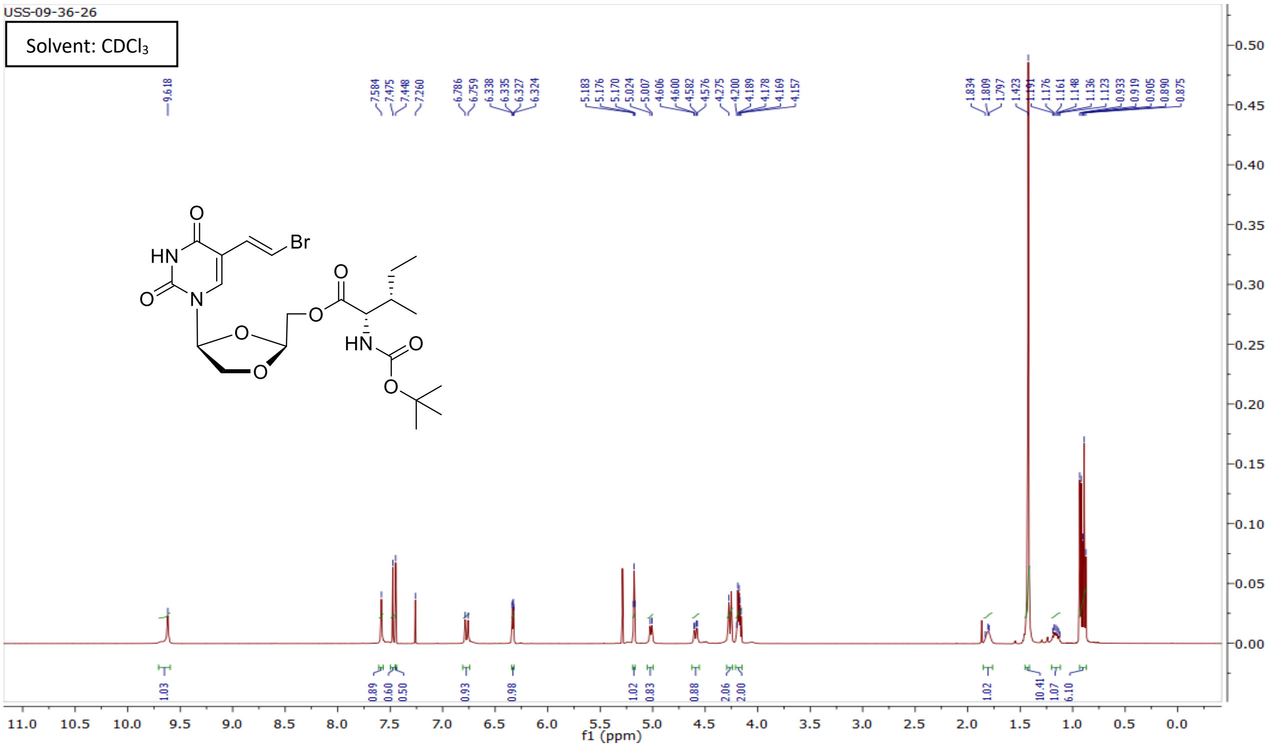

^{1}H NMR of L-BHDU-5'-*O*-L-Boc-iso-leucine ester **(9)**

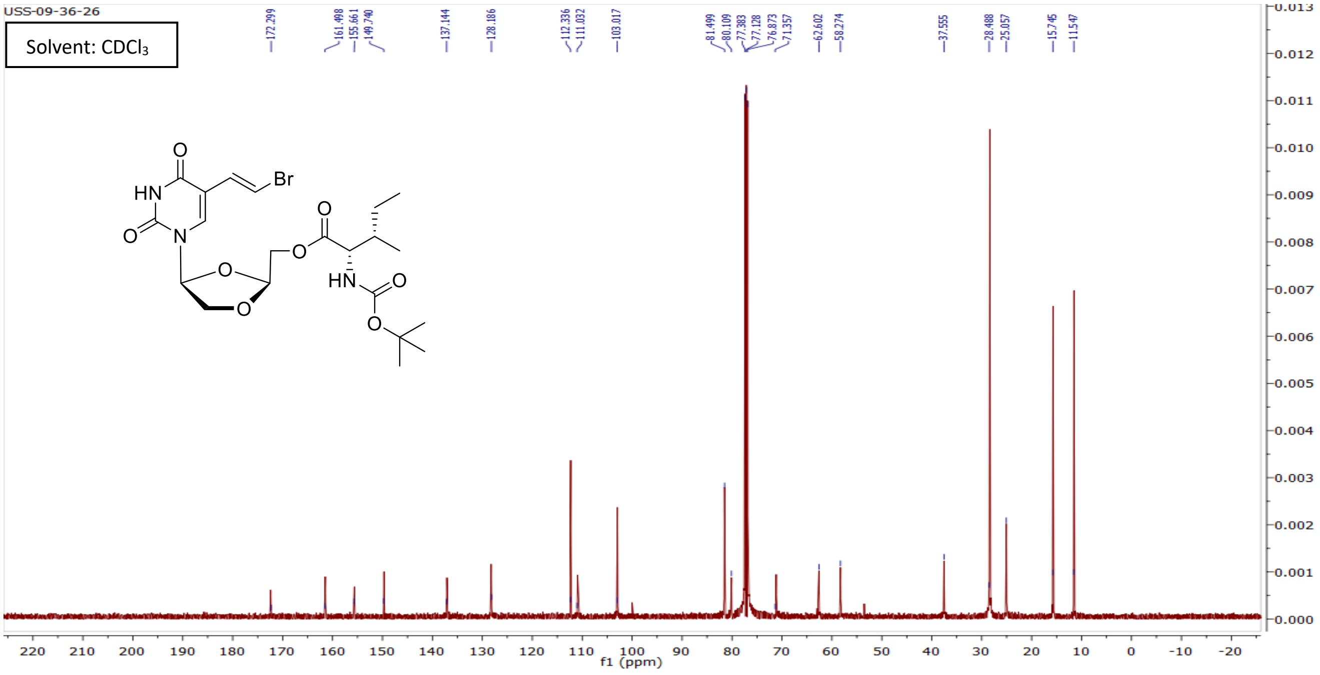

^{13}C NMR of L-BHDU-5'-*O*-L-Boc-iso-leucine ester **(9)**

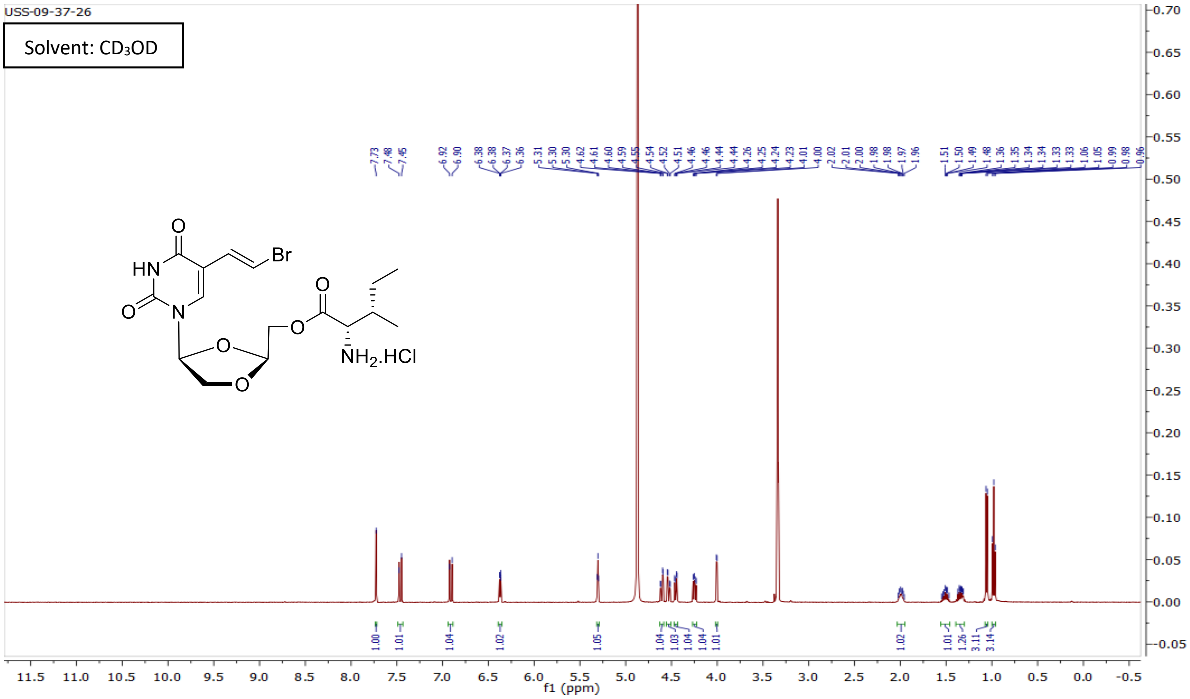

^{1}H NMR of L-BHDU-5'-*O*-L-iso-leucine ester hydrochloride **(21)**

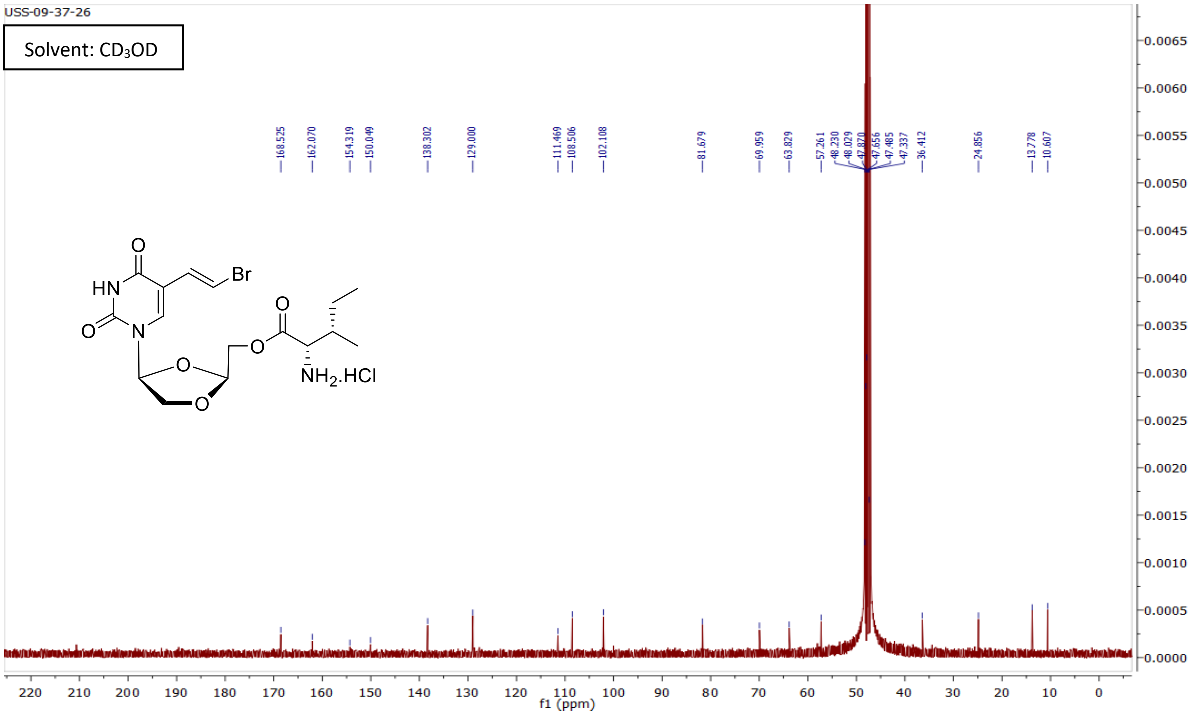

^{13}C NMR of L-BHDU-5'-*O*-L-iso-leucine ester hydrochloride **(21)**

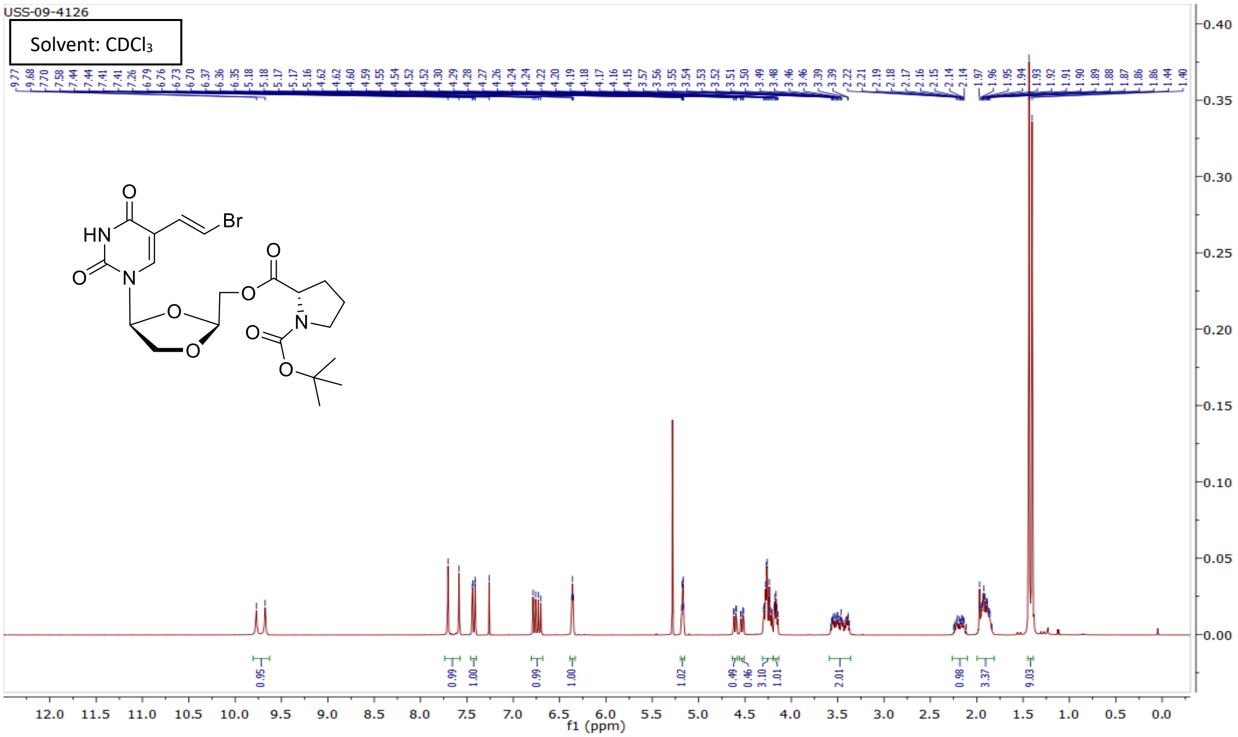

^{1}H NMR of L-BHDU-5'-*O*-L-Boc-proline ester **(10)**

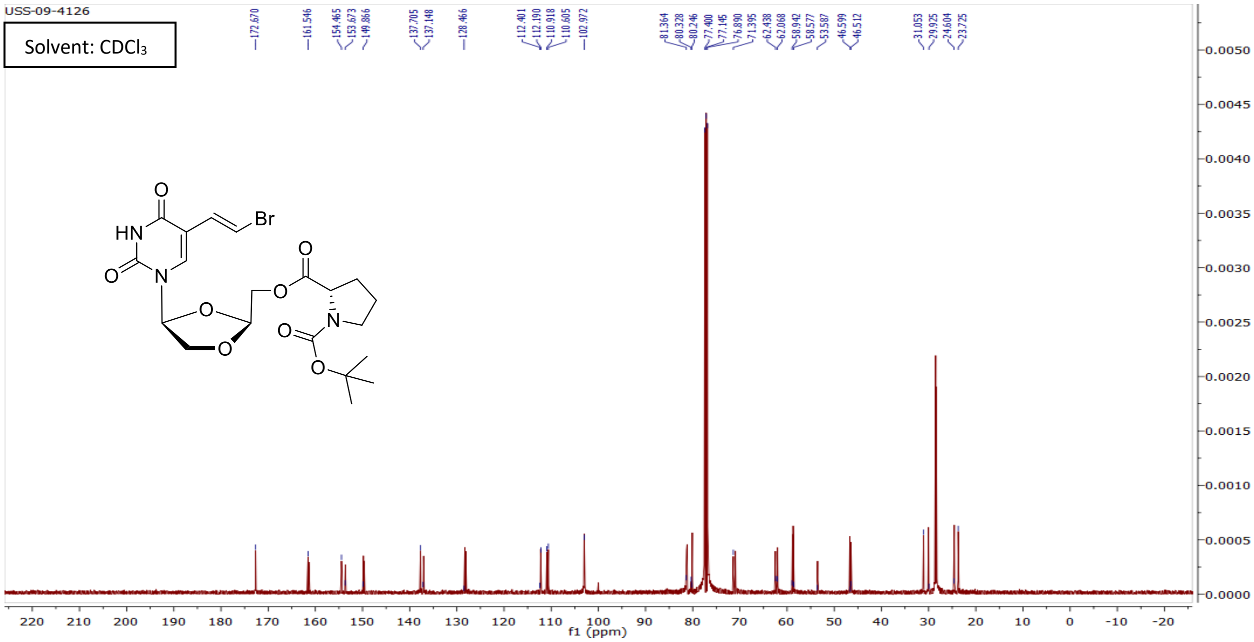

^{13}C NMR of L-BHDU-5'-*O*-L-Boc-proline ester **(10)**

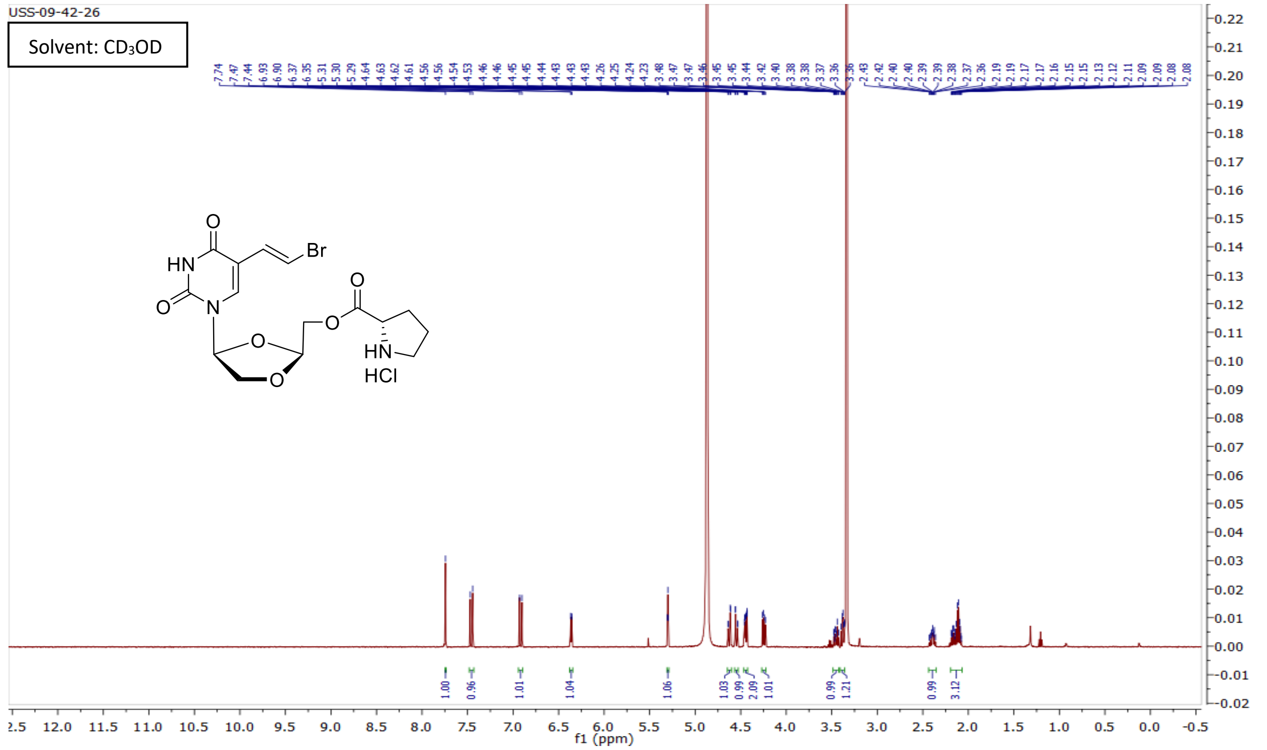

^{1}H NMR of L-BHDU-5'-*O*-L-proline ester hydrochloride **(22)**

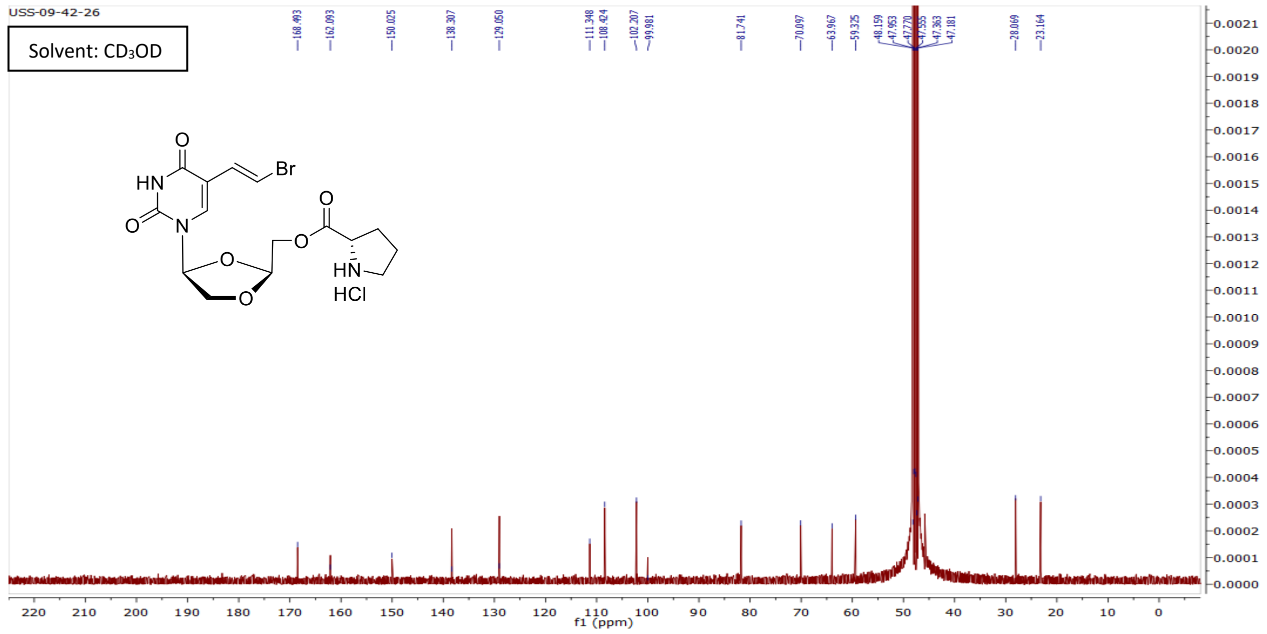

^{13}C NMR of L-BHDU-5'-*O*-L-proline ester hydrochloride **(22)**

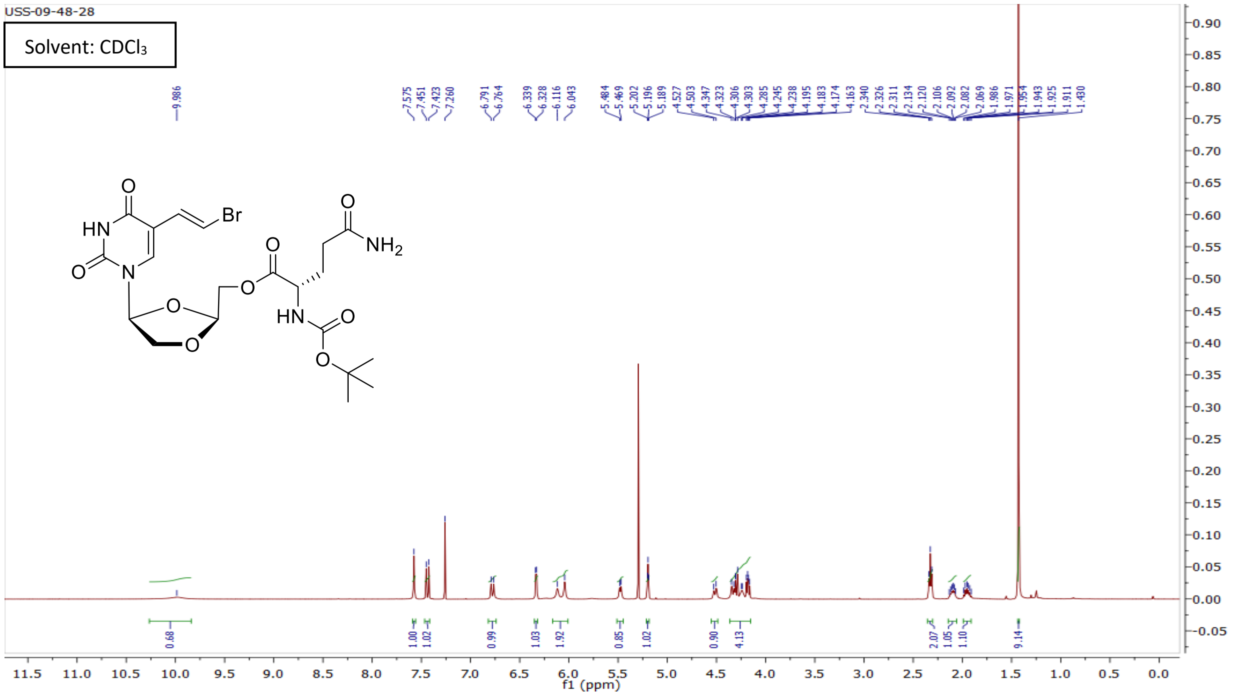

^{1}H NMR of L-BHDU-5'-*O*-L-Boc-glutamine amide ester **(11)**

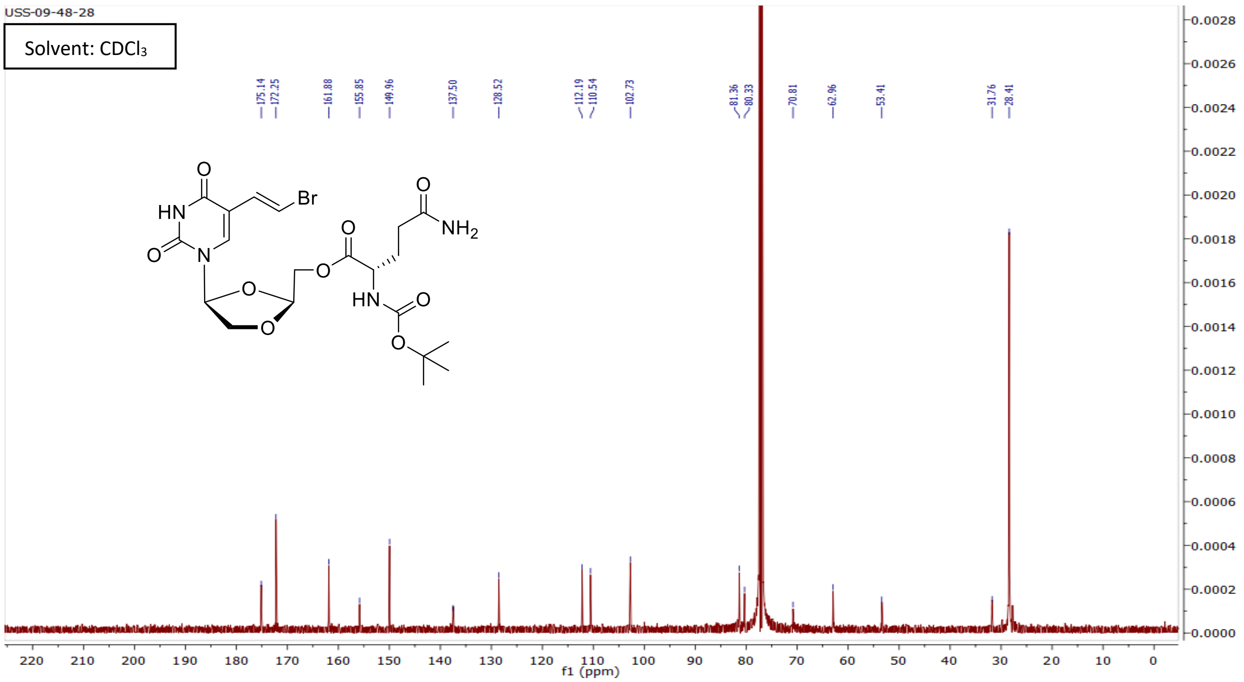

^{13}C NMR of L-BHDU-5'-*O*-L-Boc-glutamine amide ester **(11)**

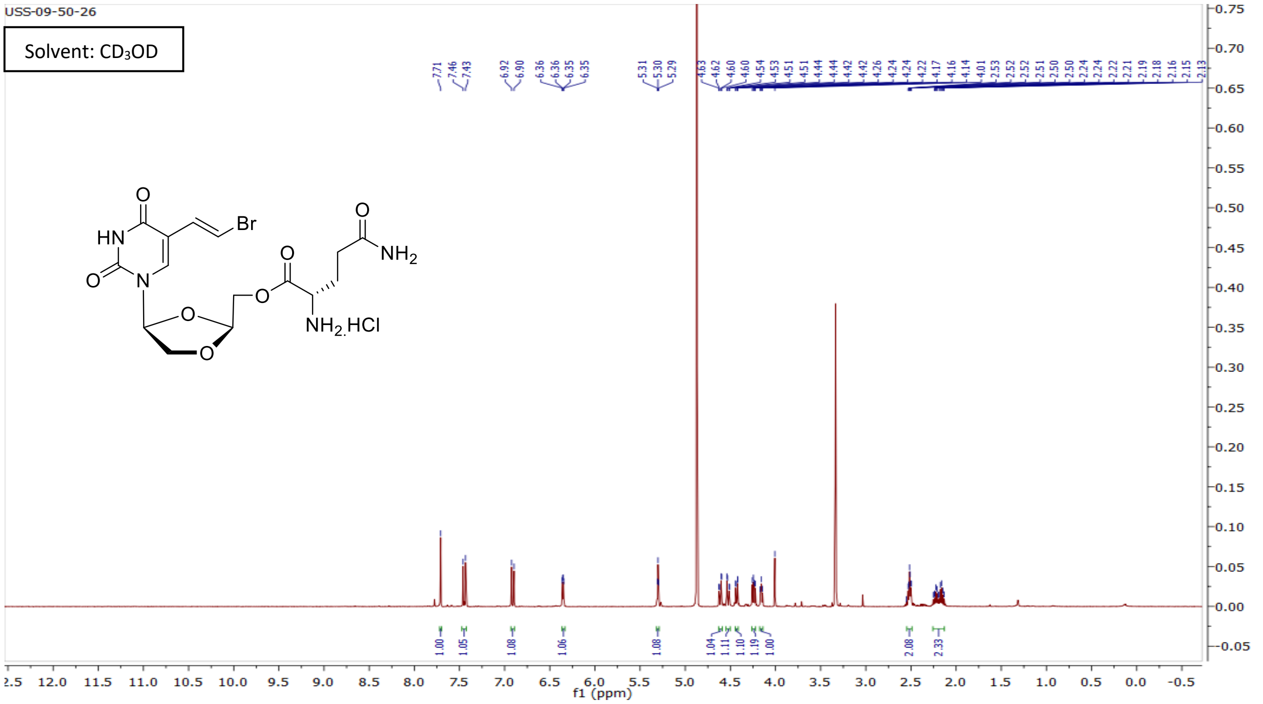

^{1}H NMR of L-BHDU-5'-*O*-L-glutamine ester hydrochloride **(23)**

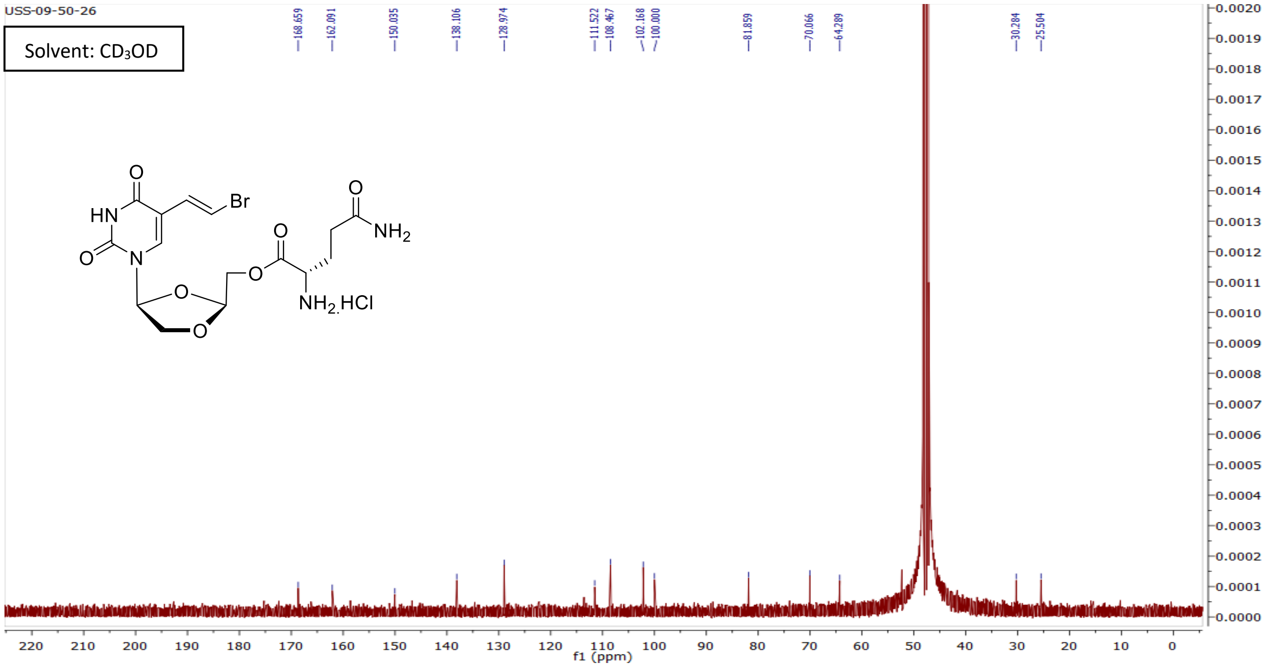

^{13}C NMR of L-BHDU-5'-*O*-L-glutamine ester hydrochloride **(23)**

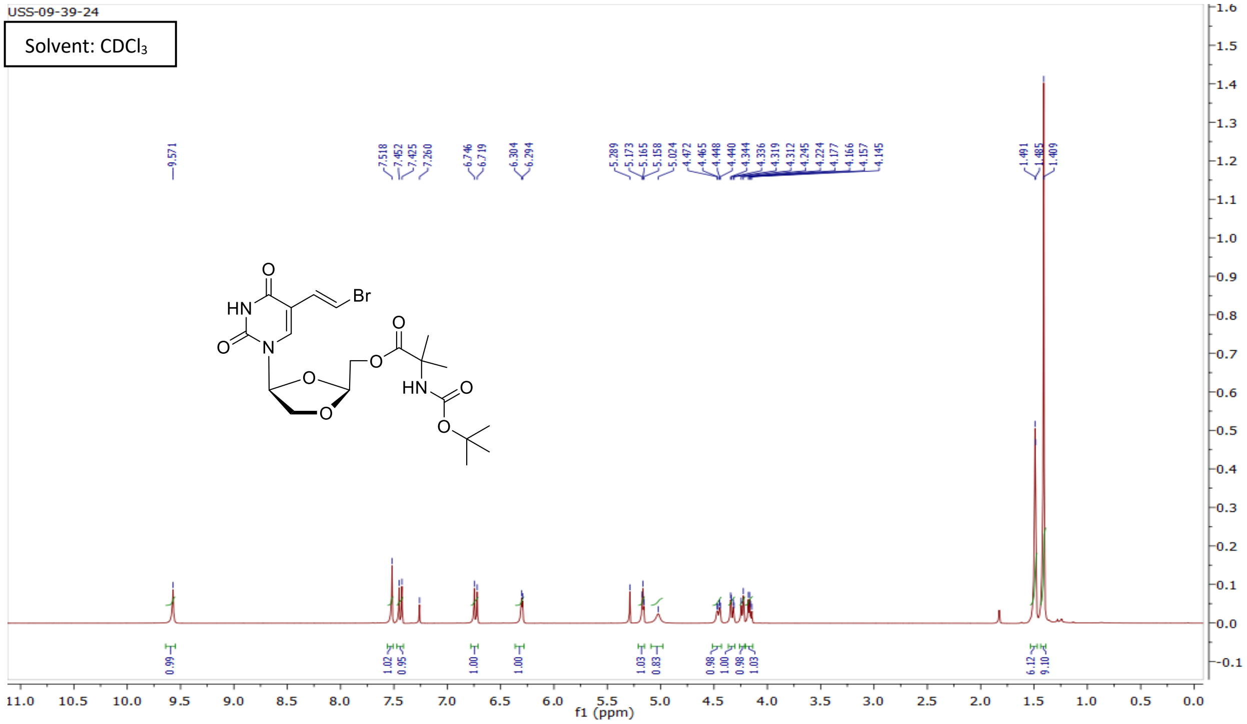

^{1}H NMR of L-BHDU-5'-*O*-Boc-2-Aminoisobutyric ester **(12)**

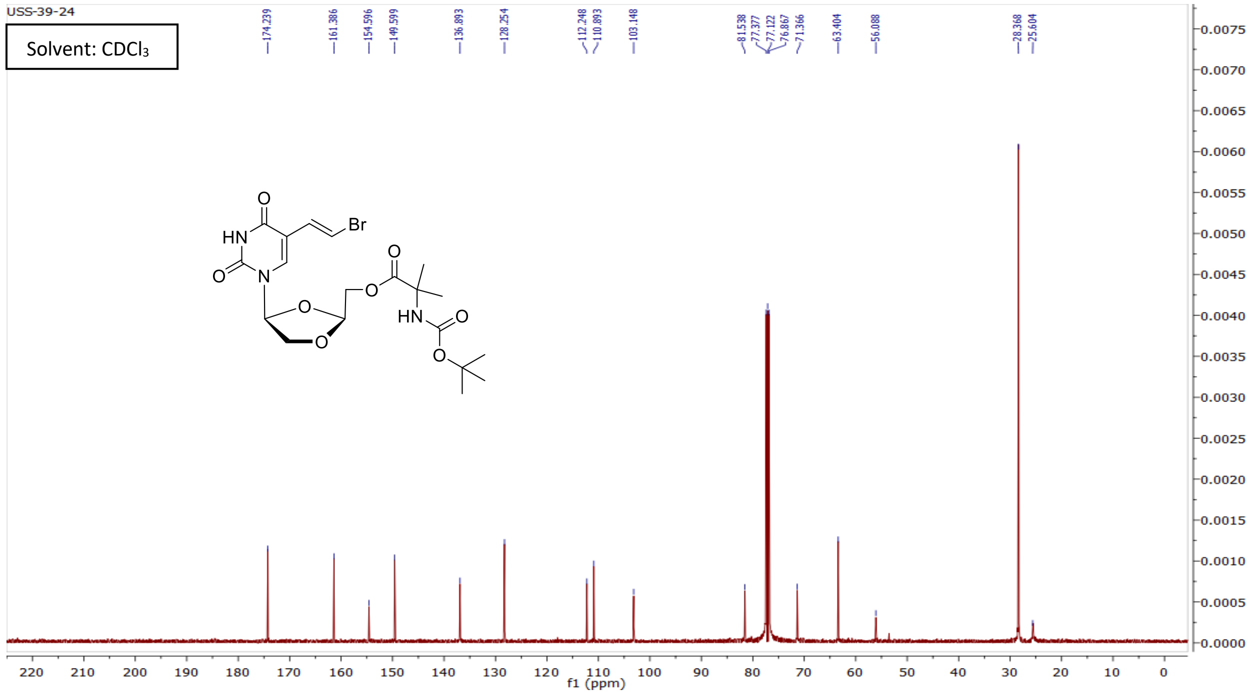

^{13}C NMR of L-BHDU-5'-*O*-Boc-2-Aminoisobutyric ester **(12)**

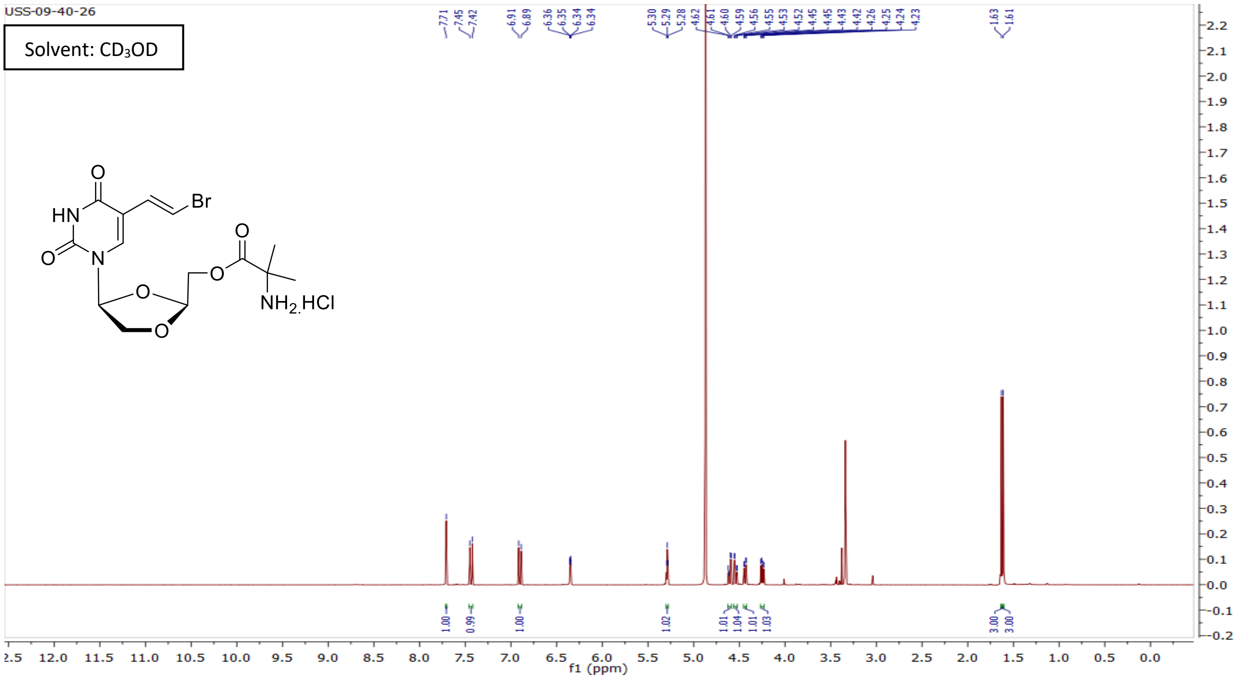

^{1}H NMR of L-BHDU-5'-*O*-2-Aminoisobutyric acid ester hydrochloride **(24)**

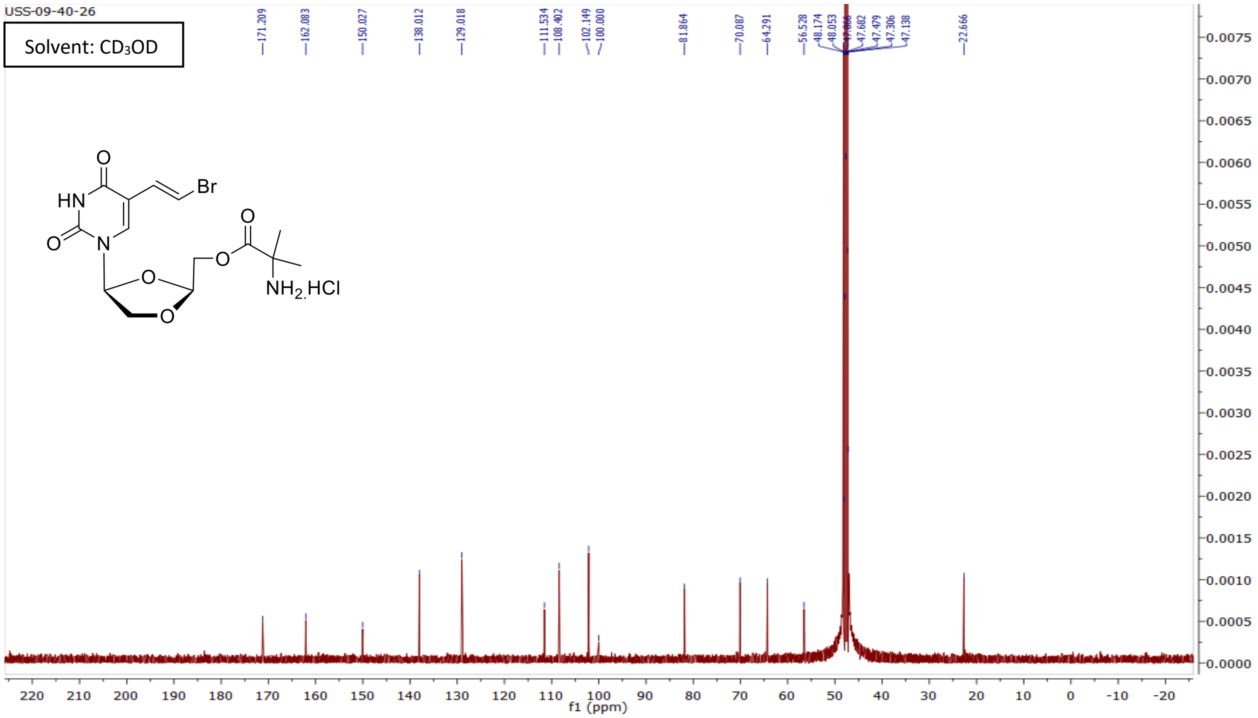

^{13}C NMR of L-BHDU-5'-*O*-2-Aminoisobutyric acid ester hydrochloride **(24)**

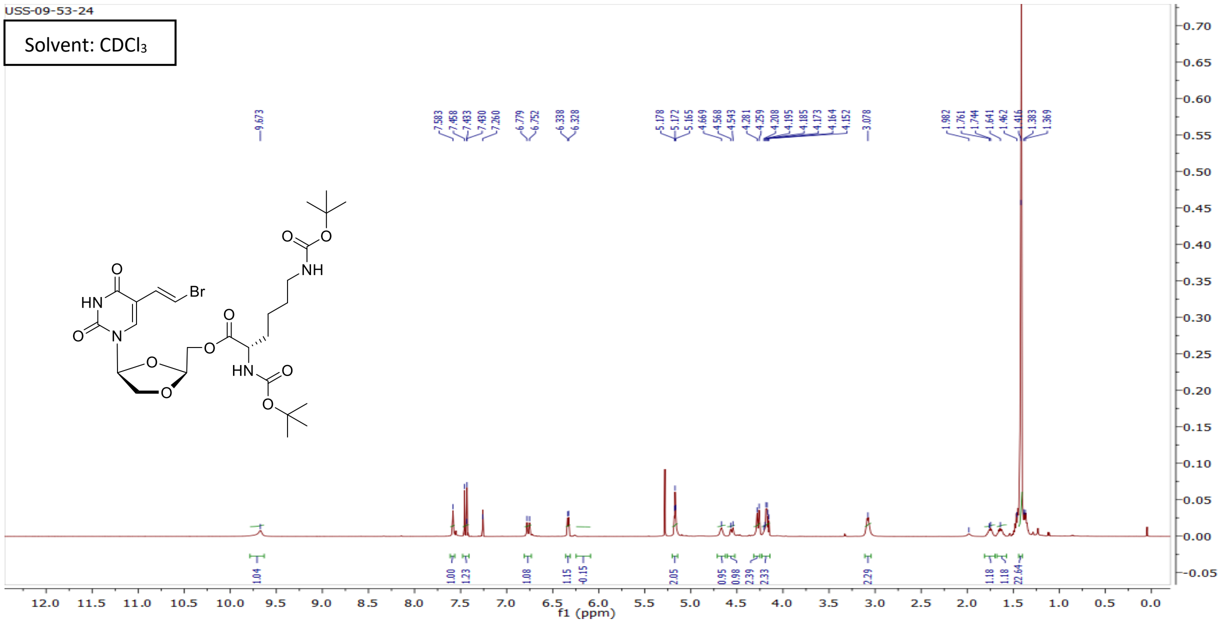

^{1}H NMR of L-BHDU-5'-*O*-L-Boc-lysine Boc-amide ester **(13)**

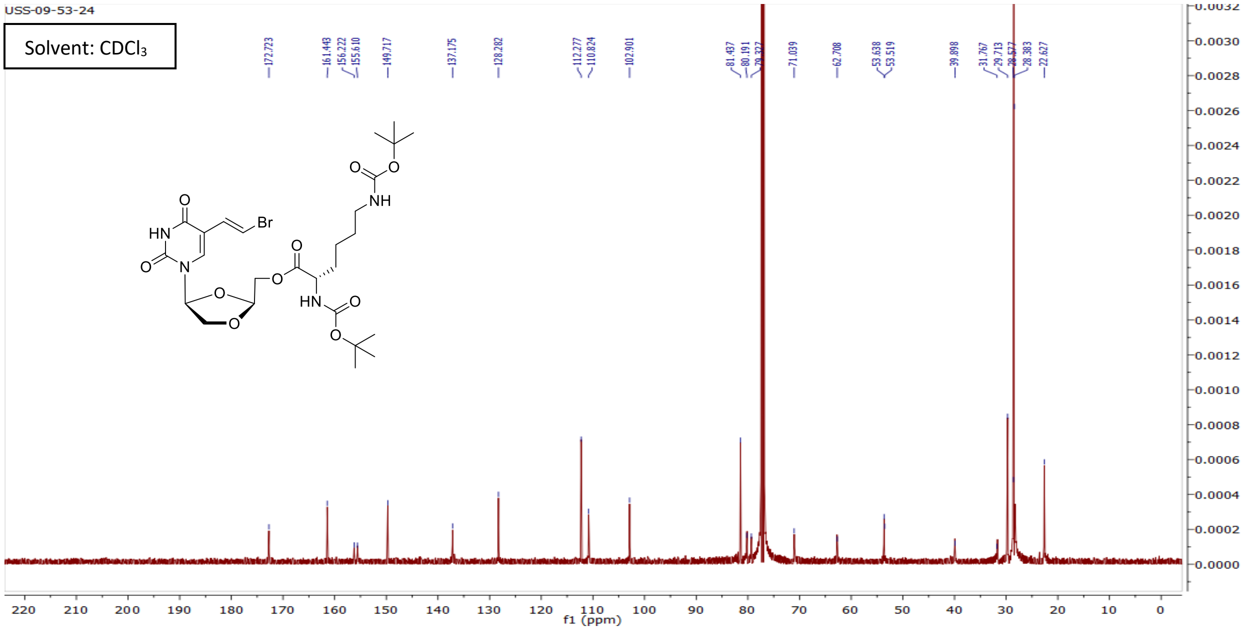

^{13}C NMR of L-BHDU-5'-*O*-L-Boc-lysine Boc-amide ester **(13)**

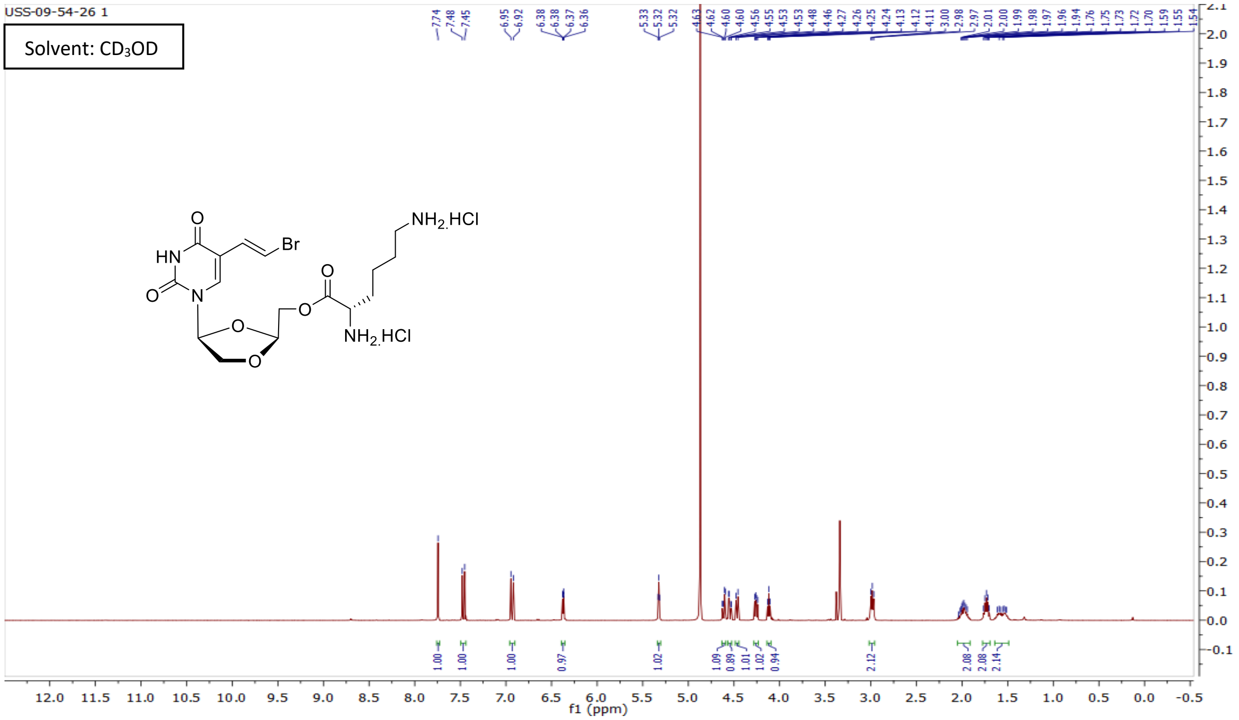

^{1}H NMR of L-BHDU-5'-*O*-L-lysine ester hydrochloride **(25)**

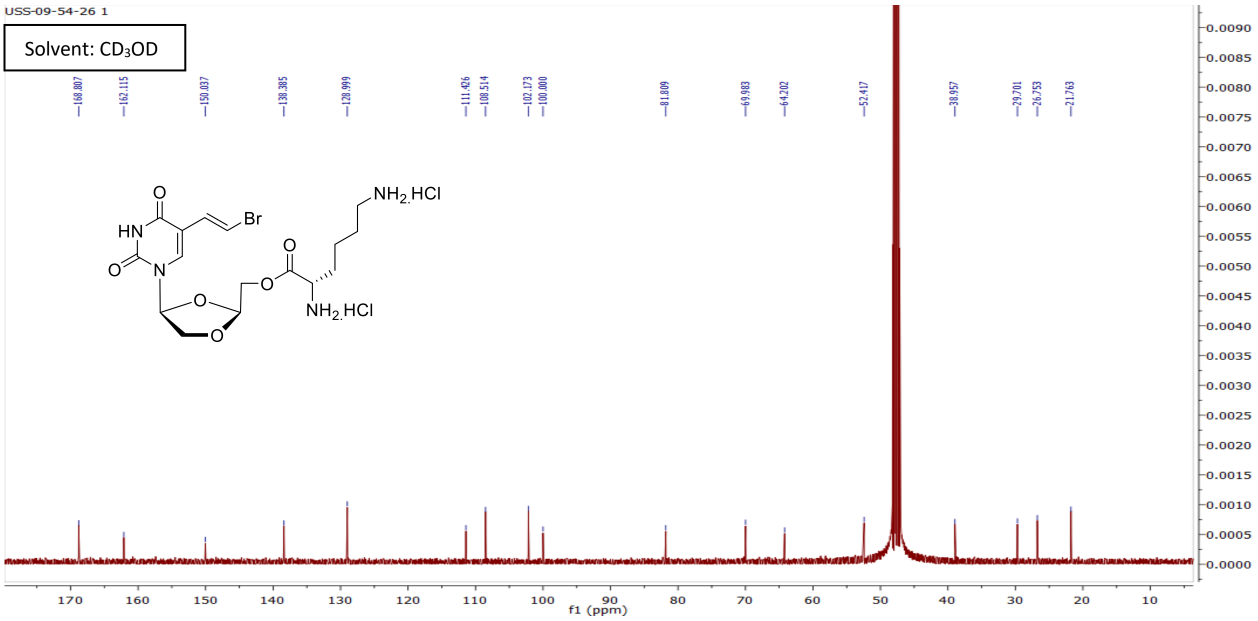

^{13}C NMR of L-BHDU-5'-*O*-L-lysine ester hydrochloride **(25)**

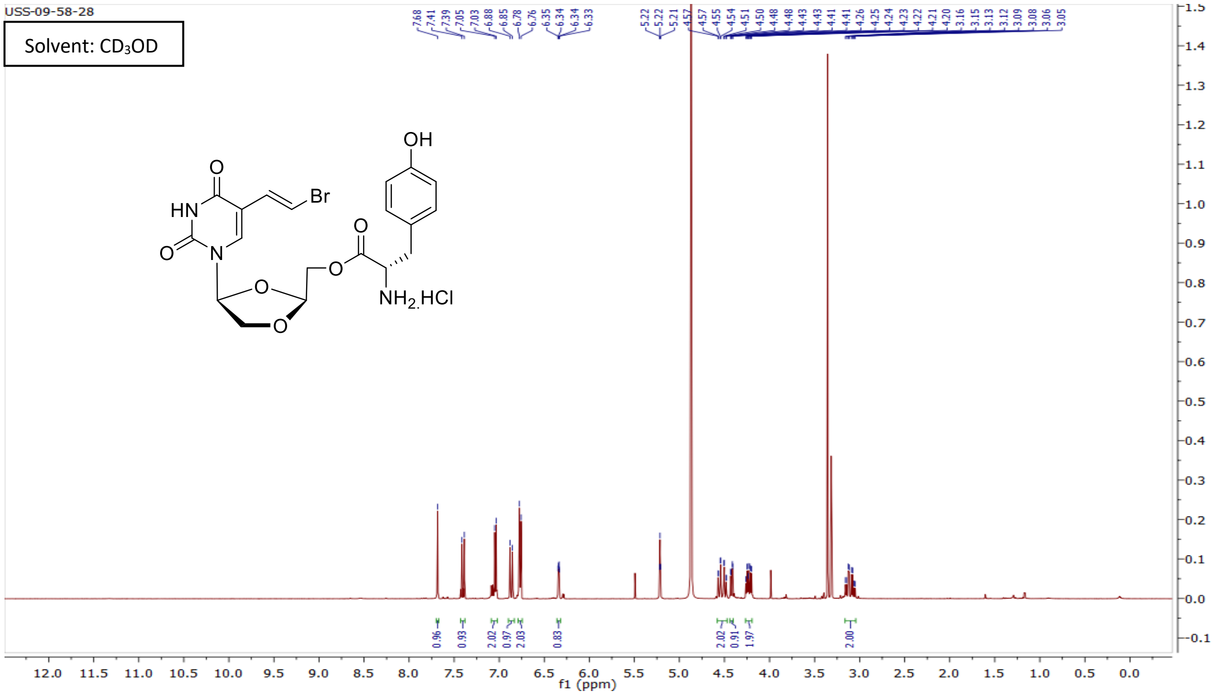

^{1}H NMR of L-BHDU-5′-*O*-L-tyrosine ester hydrochloride **(26)**

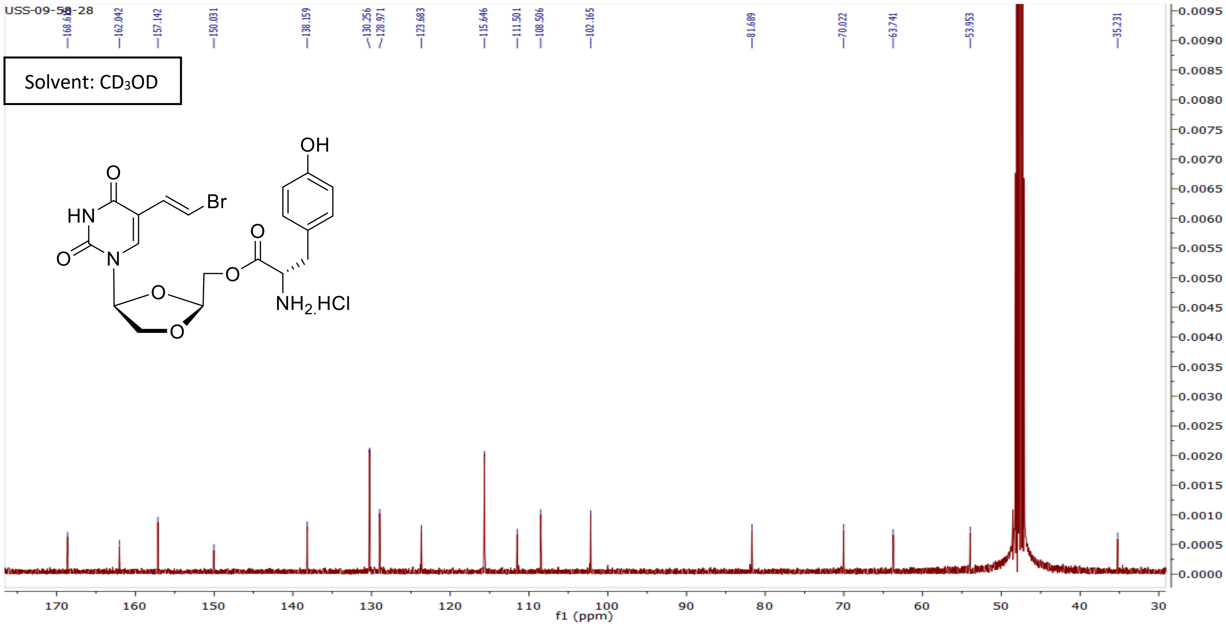

^{13}C NMR of L-BHDU-5′-*O*-L-tyrosine ester hydrochloride **(26)**

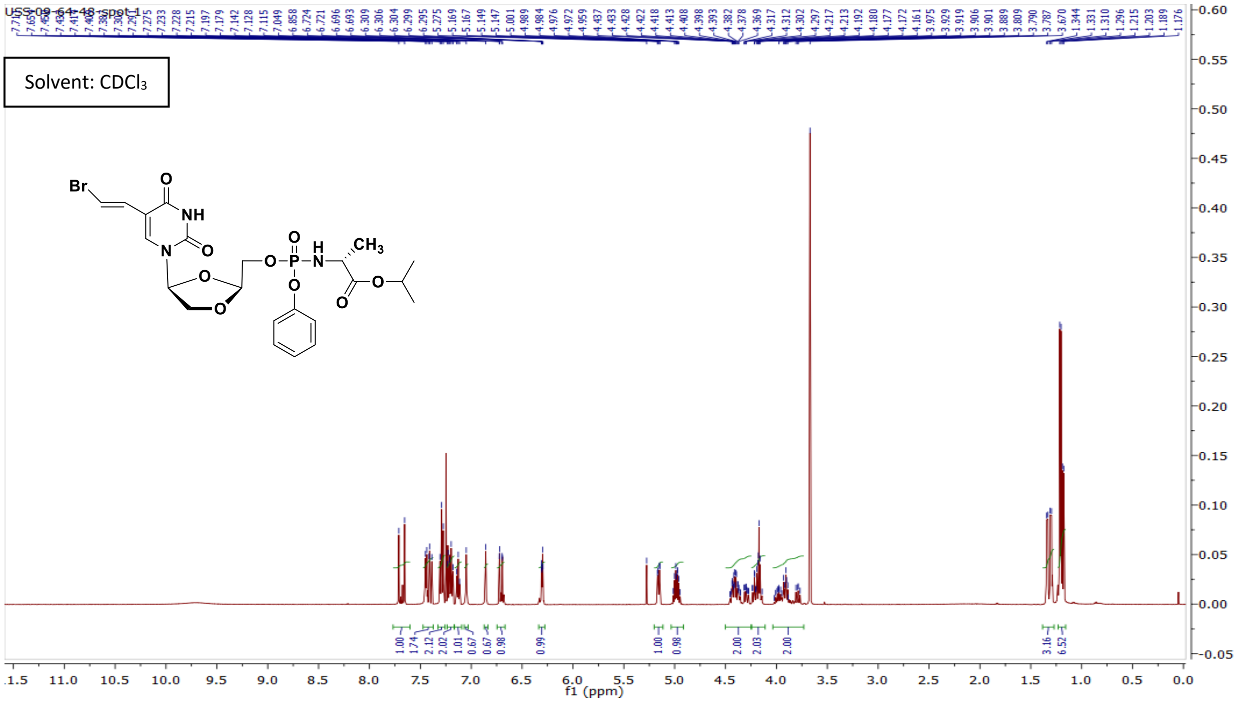

^{1}H NMR of compound **33.**

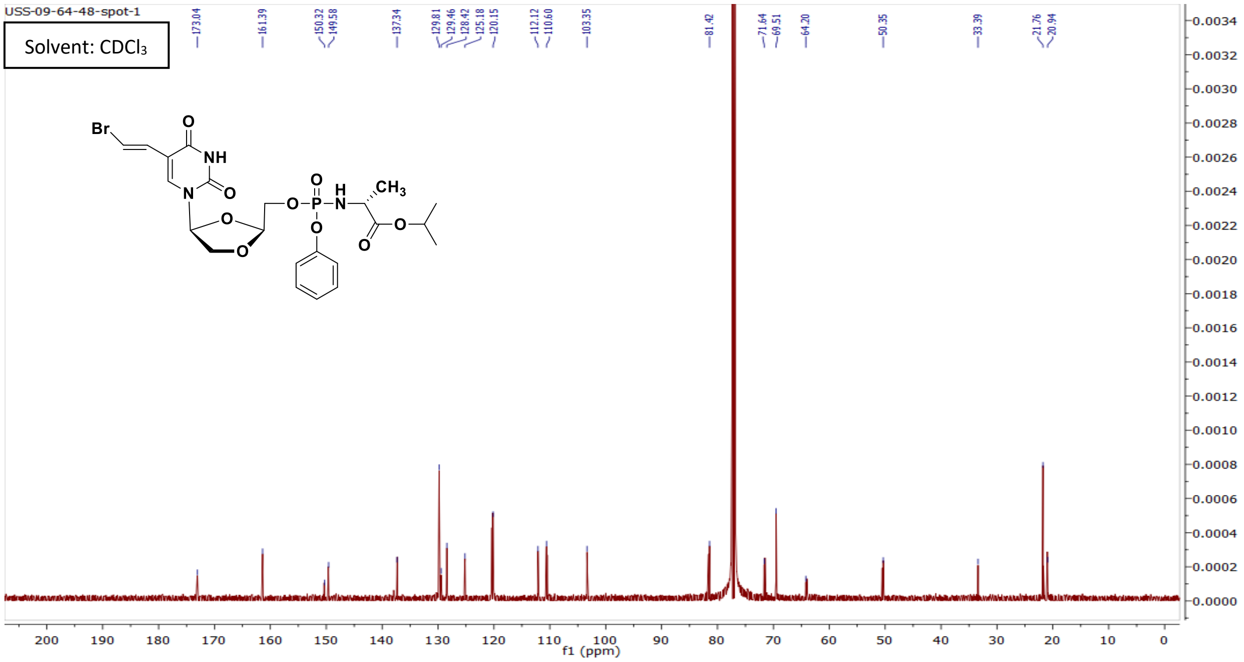

^{13}C NMR of compound **33.**

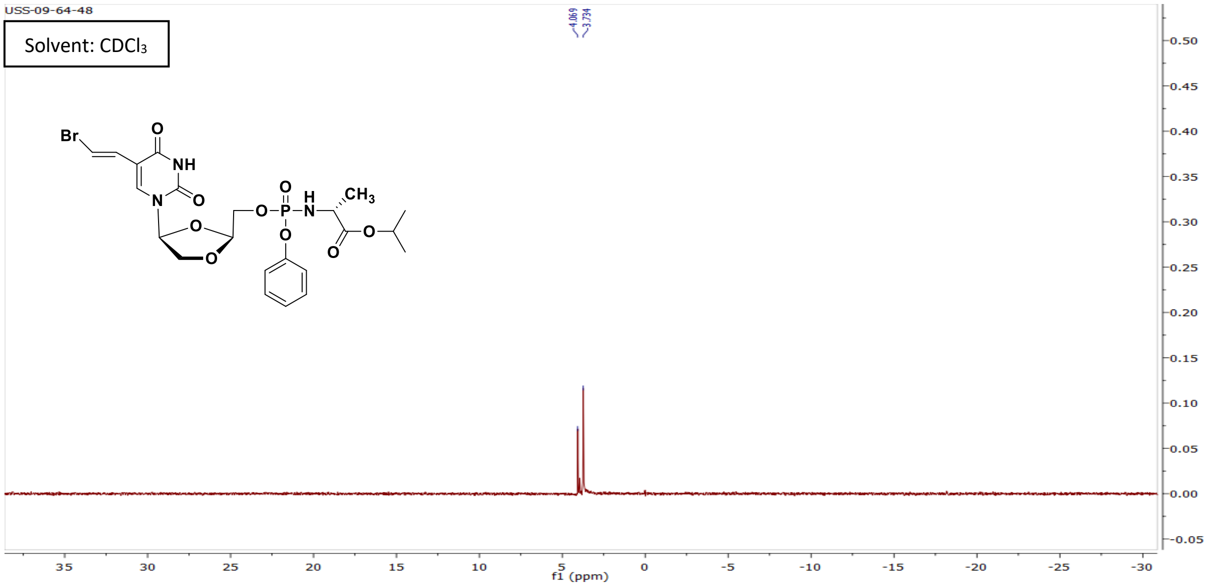

^{31}P NMR of compound **33**.

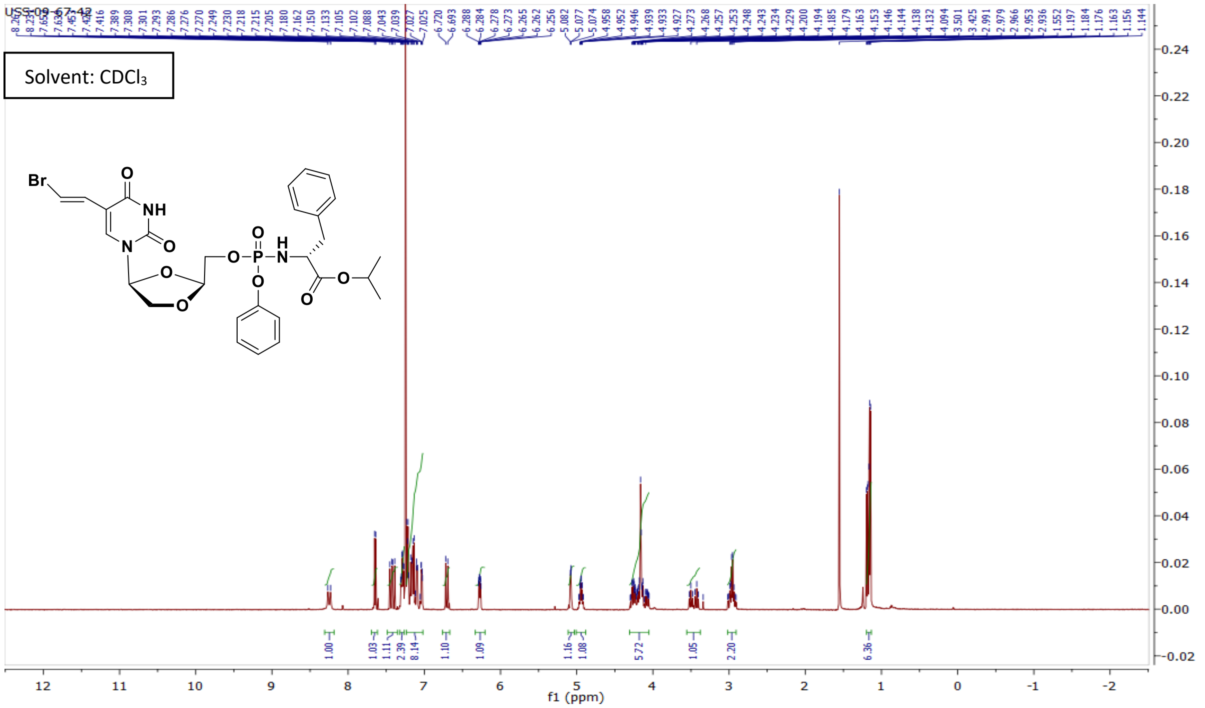

^{1}H NMR of compound **34**.

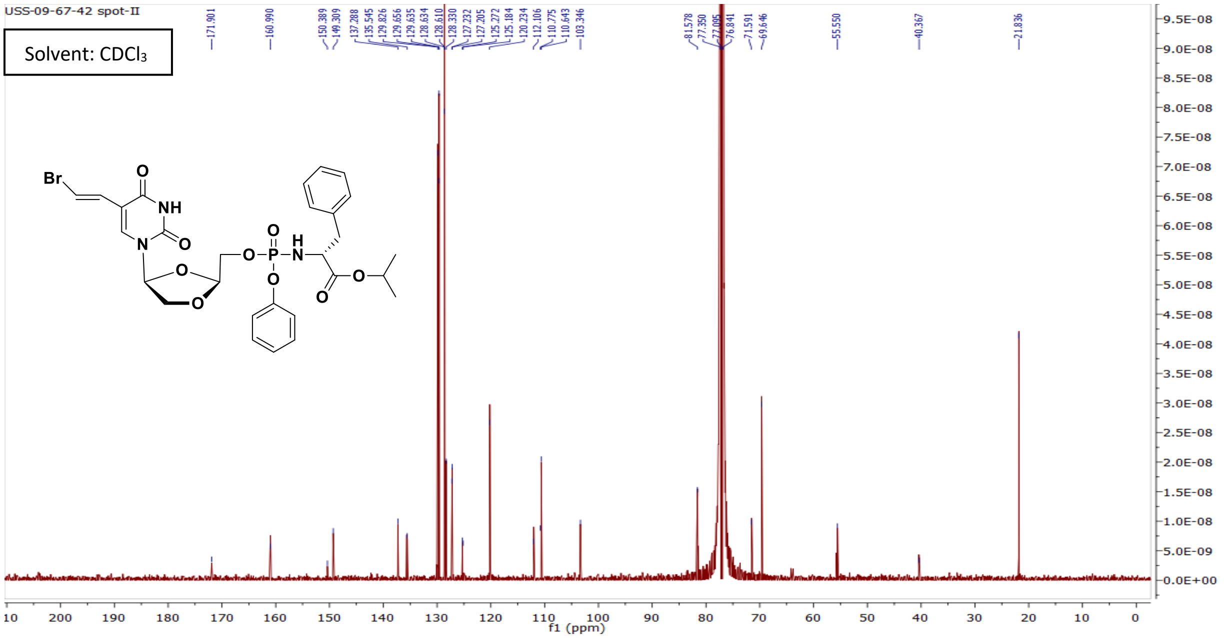

^{13}C NMR of compound **34**.

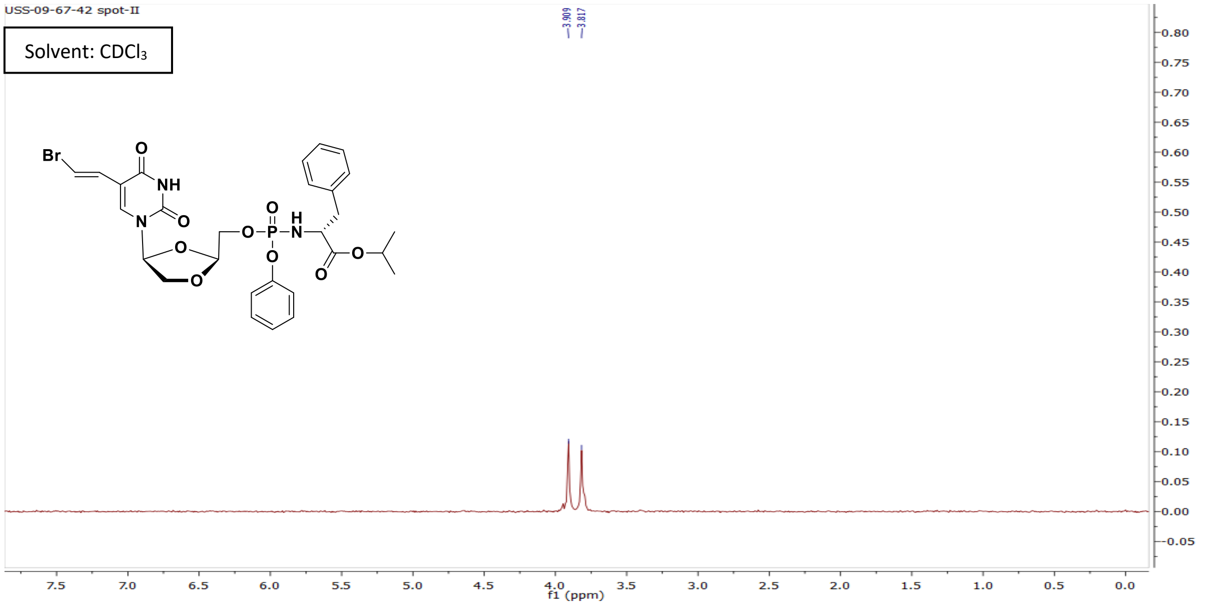

^{31}P NMR of compound **34**.

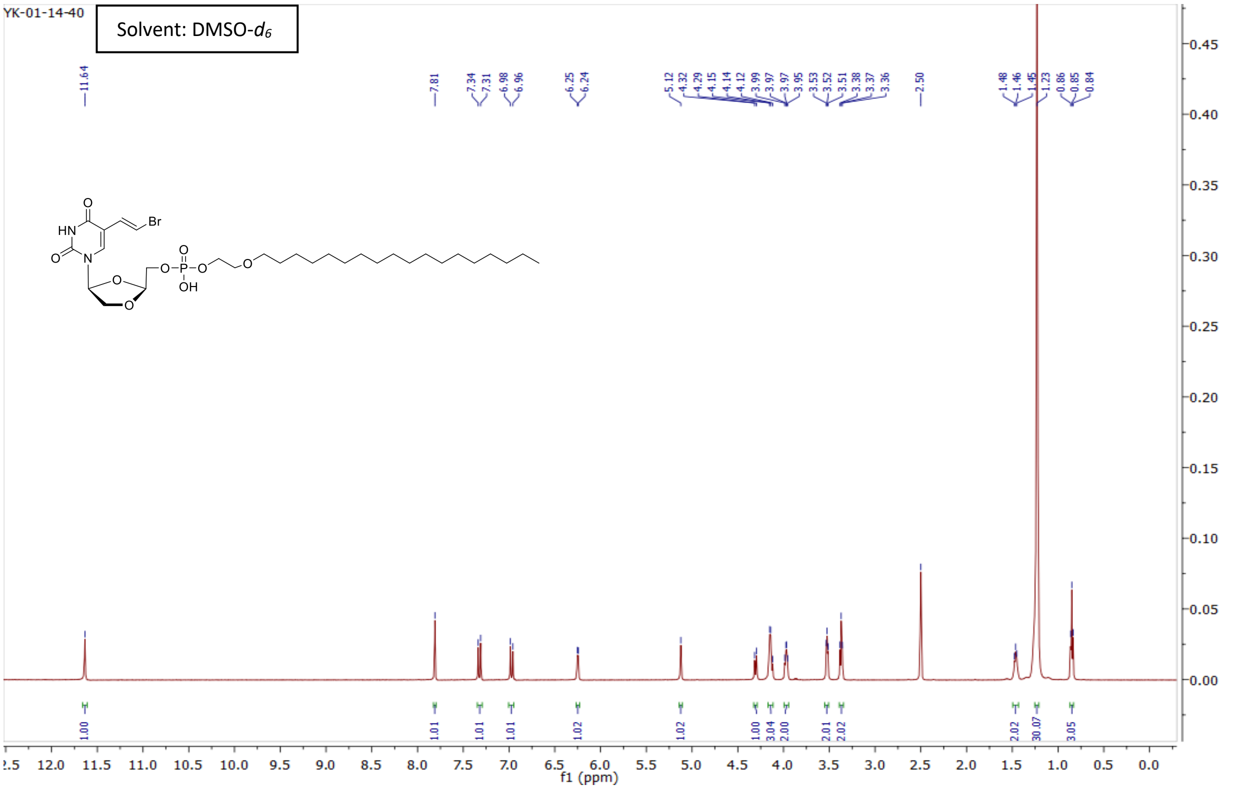

^{1}H NMR of ODE-L-BHDU-MP **(38)**.

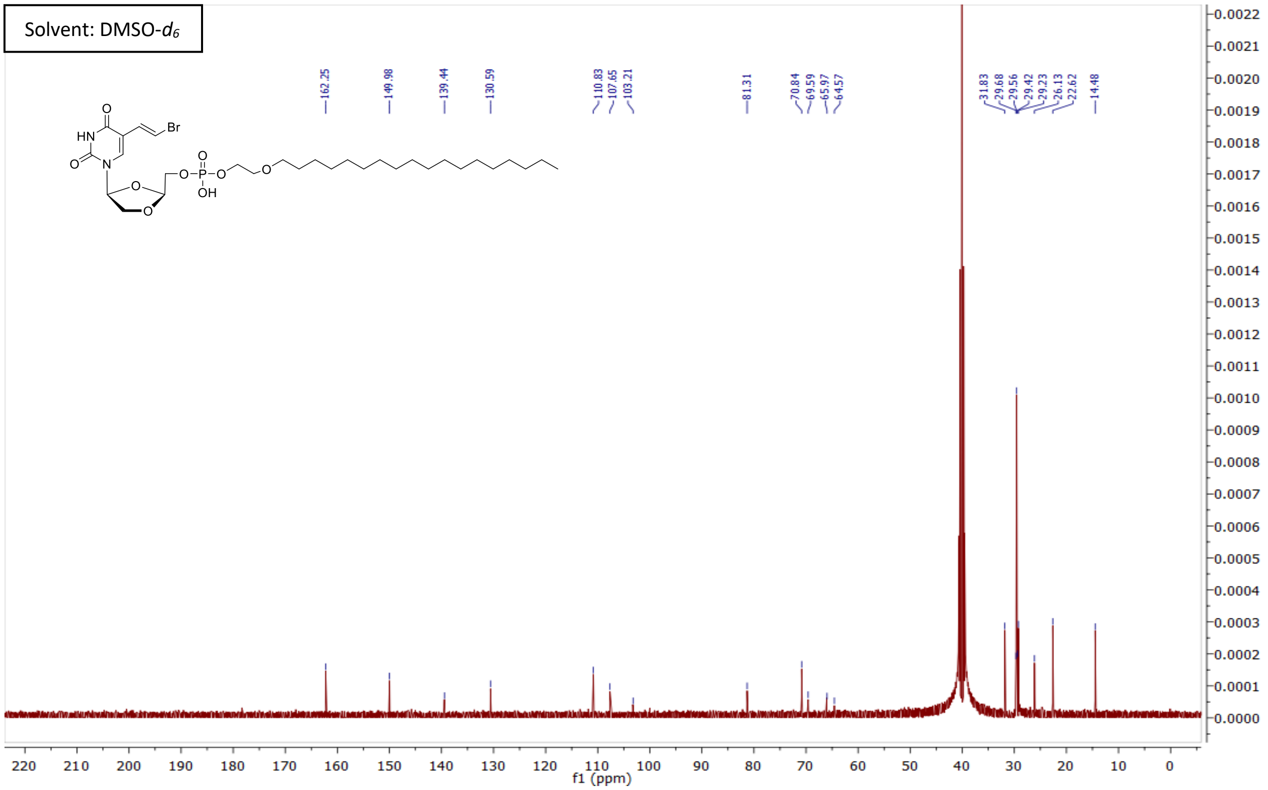

^{13}C NMR of ODE-L-BHDU-MP **(38)**.

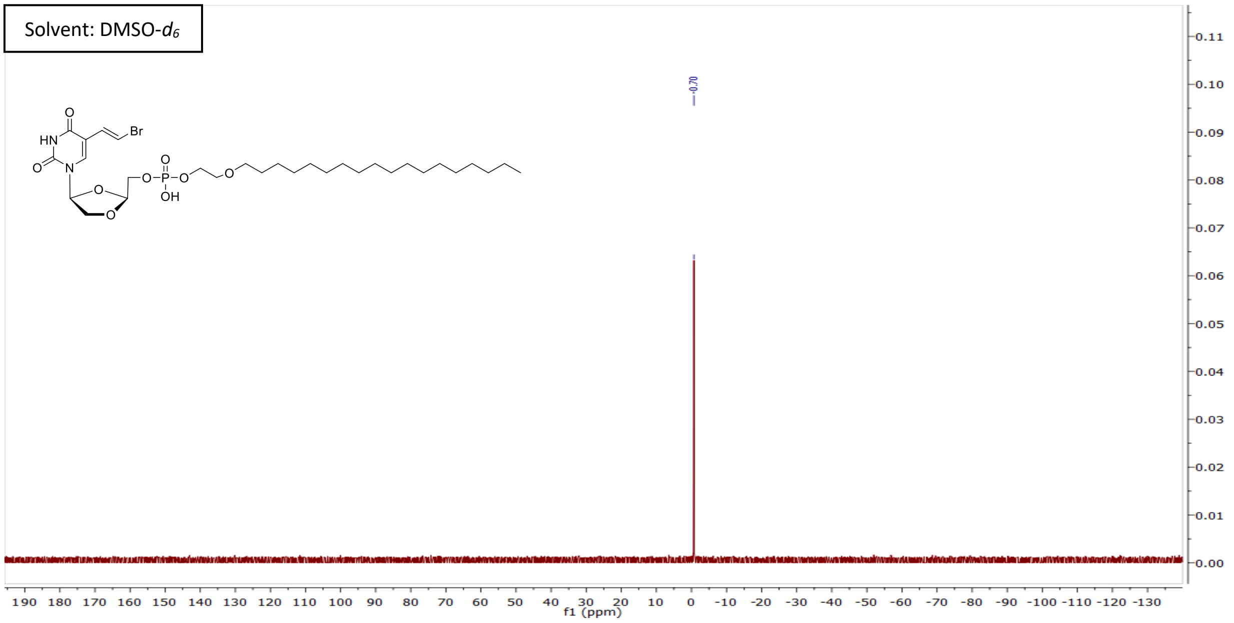

^{31}P NMR of ODE-L-BHDU-MP **(38)**.

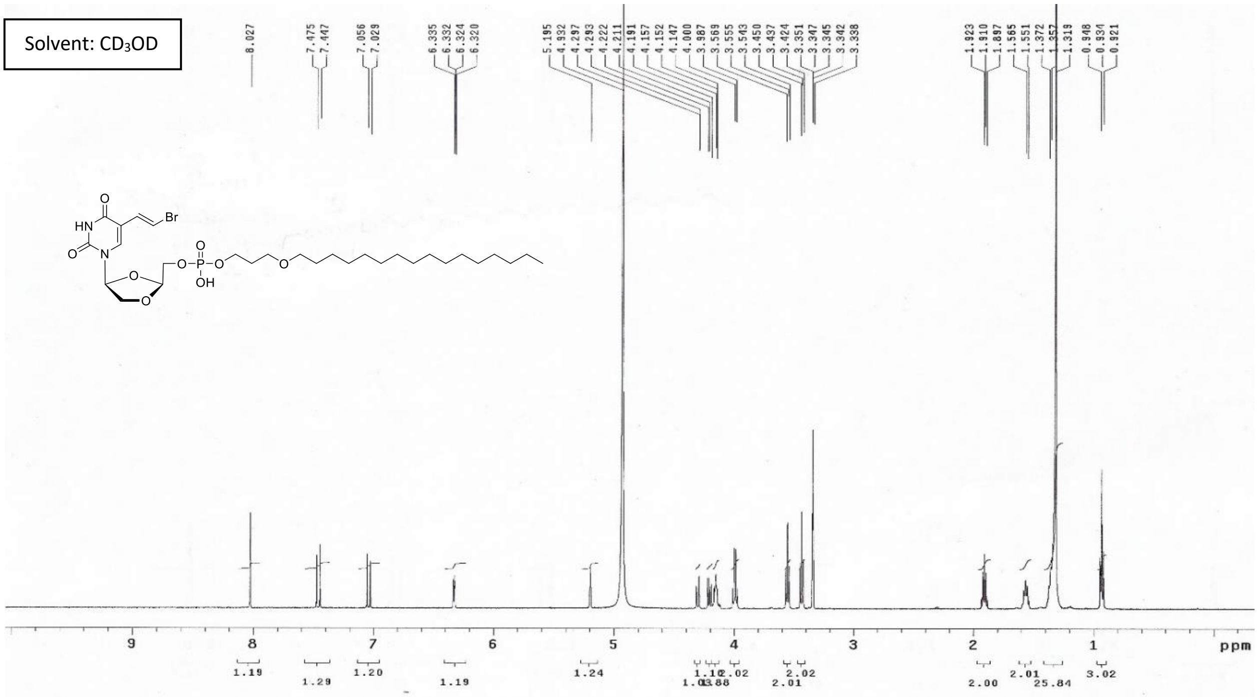

^{1}H NMR of HDP-L-BHDU-MP **(39)**.

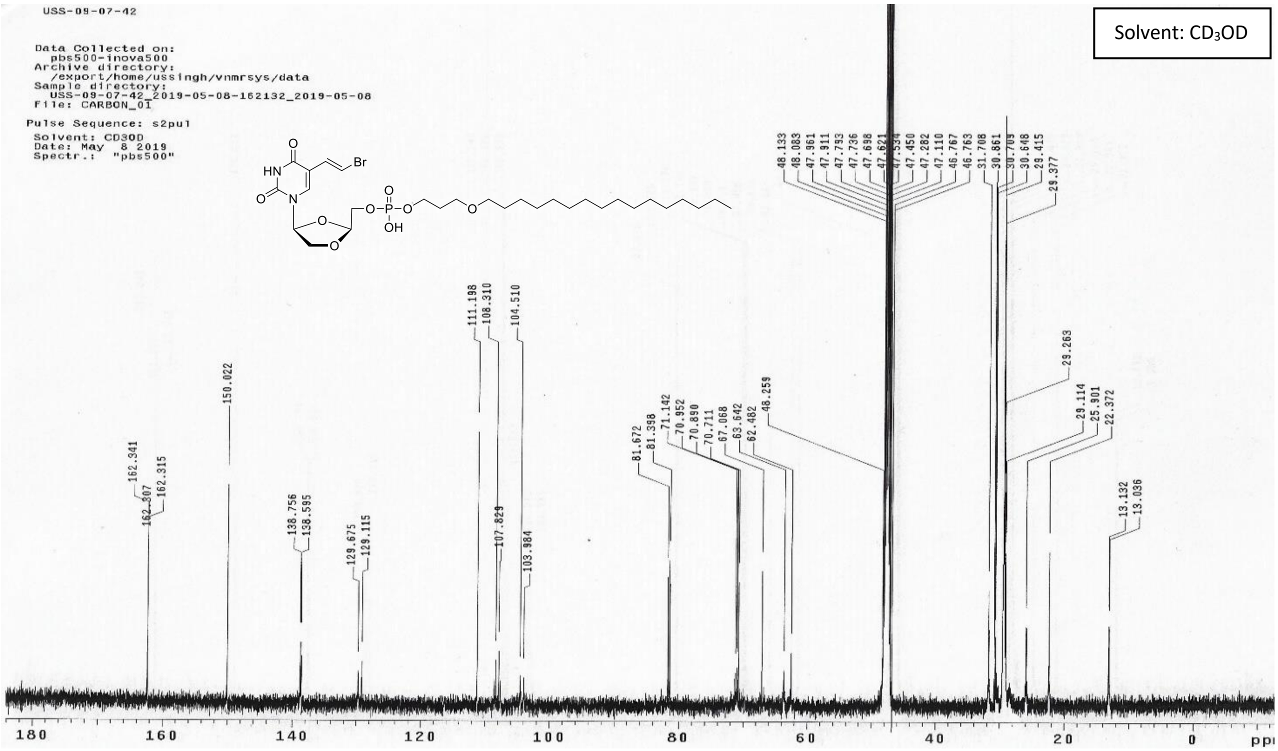

^{13}C NMR of HDP-L-BHDU-MP **(39)**.

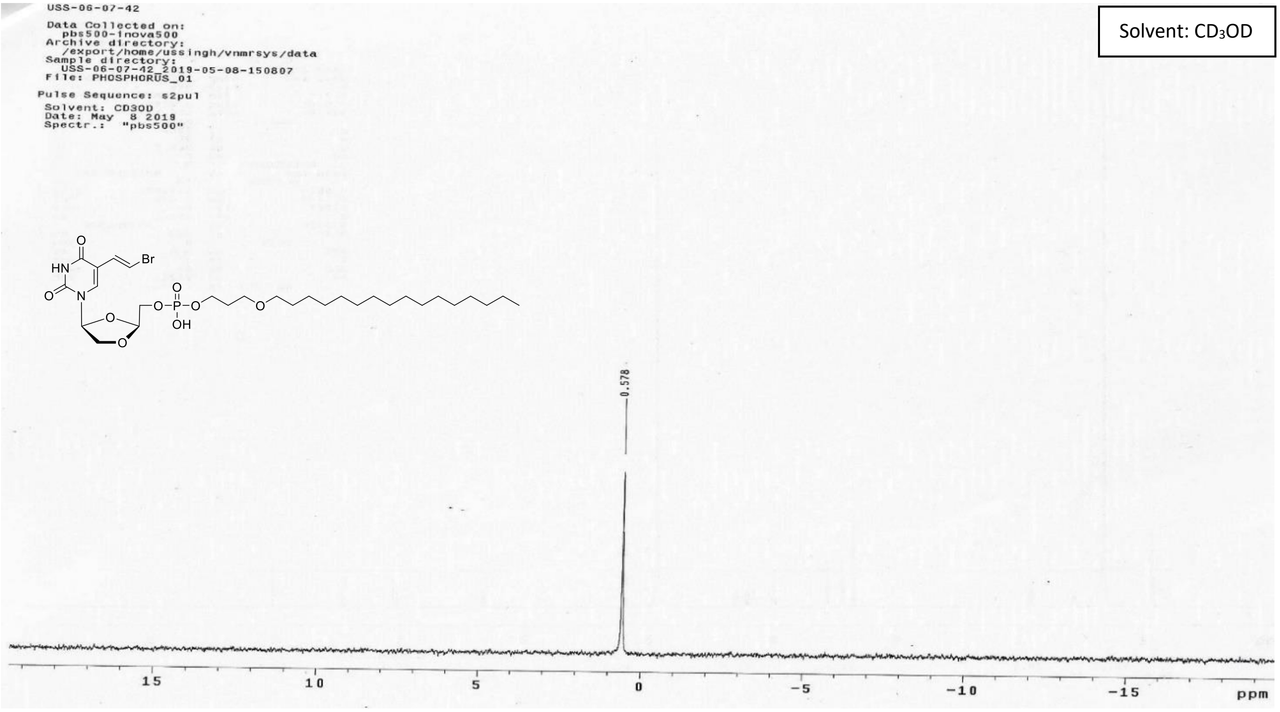

^{31}P NMR of HDP-L-BHDU-MP **(39)**.

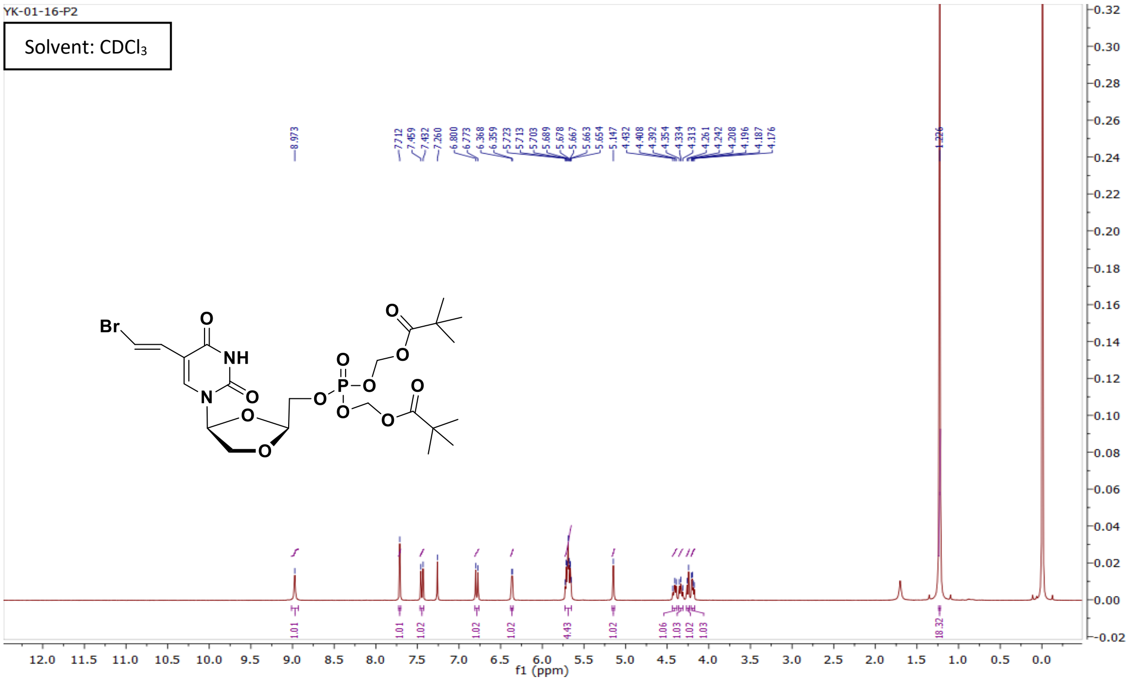

^{1}H NMR of POM-L-BHDU-MP **(41)**.

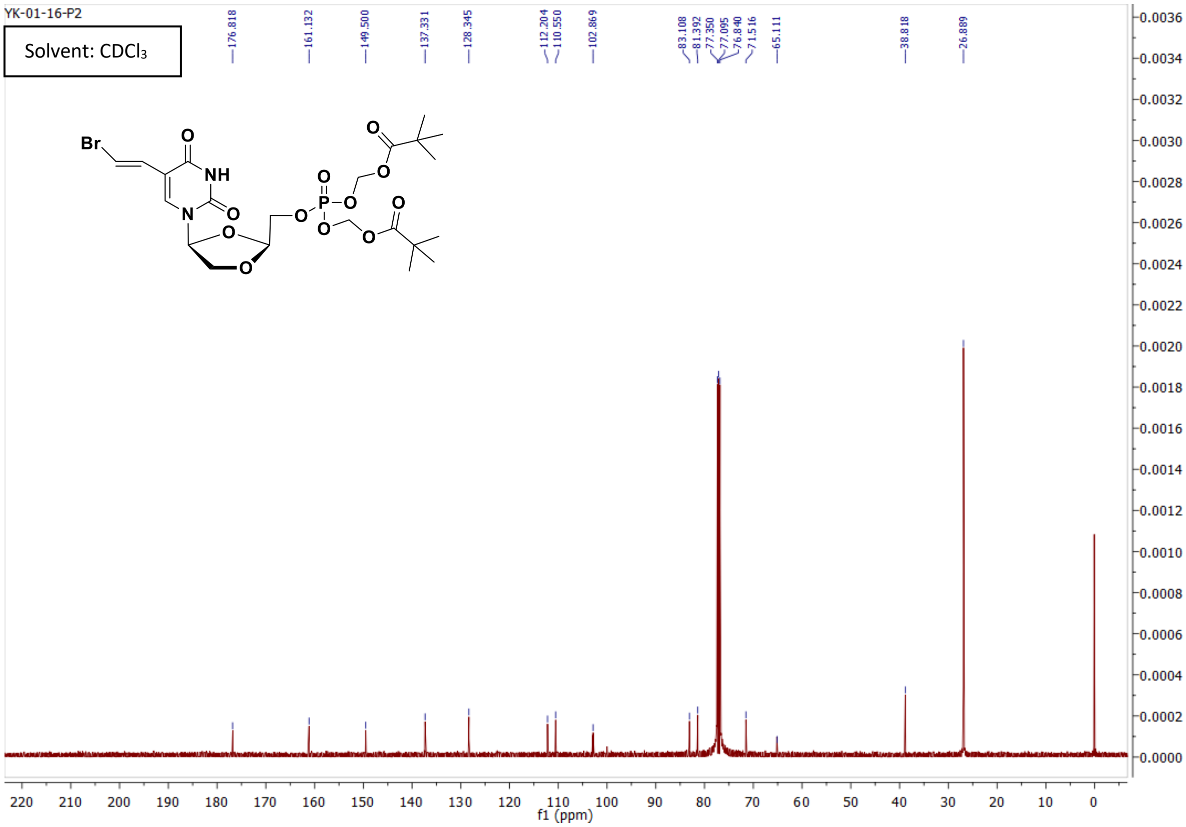

^{13}C NMR of POM-L-BHDU-MP **(41)**.

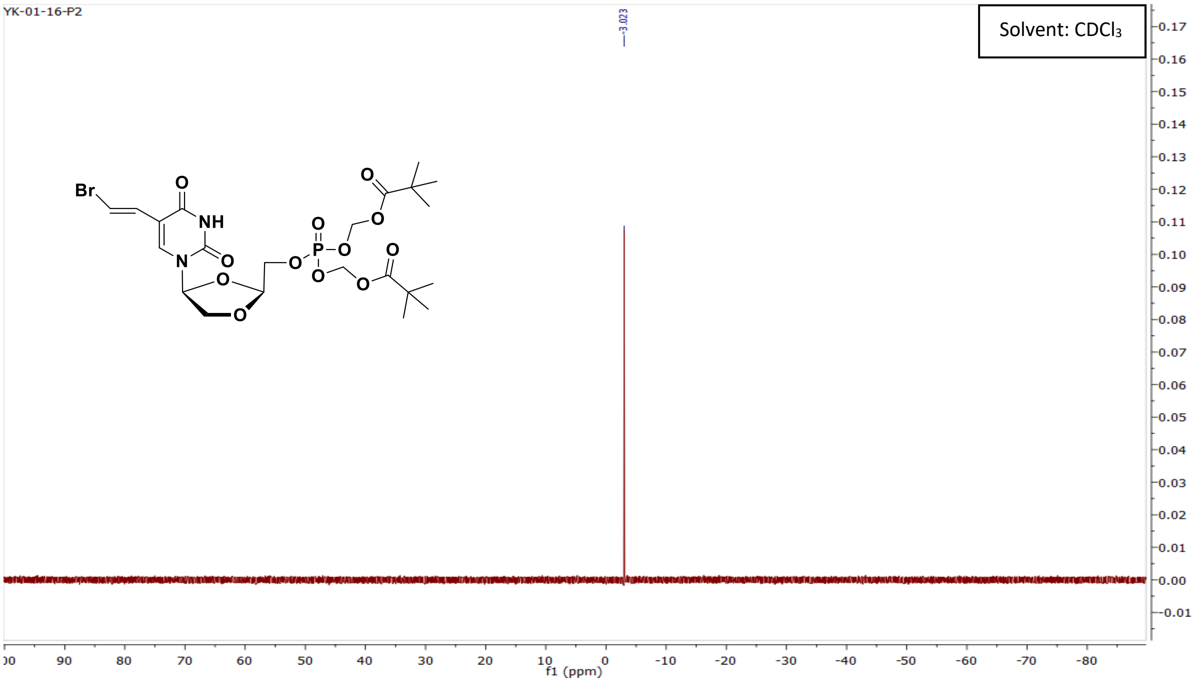

^{31}P NMR of POM-L-BHDU-MP **(41)**.

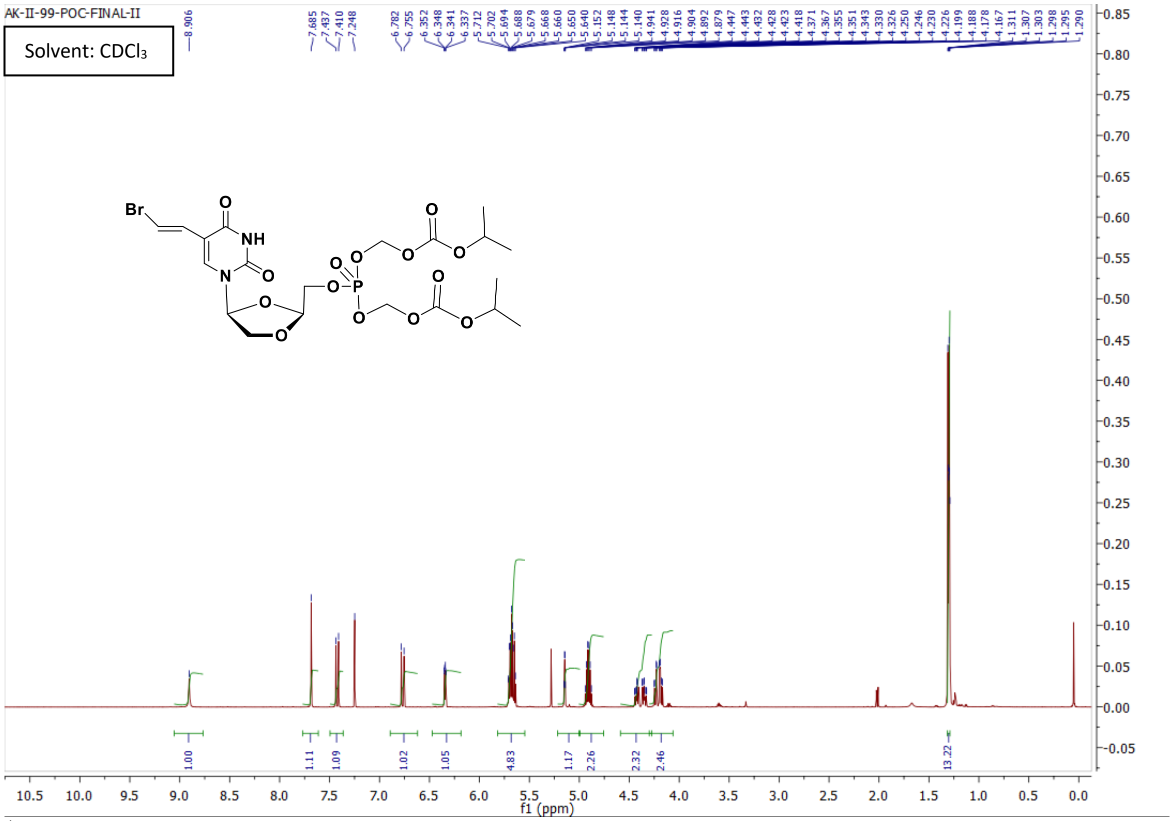

^{1}H NMR of POC-L-BHDU-MP **(47)**.

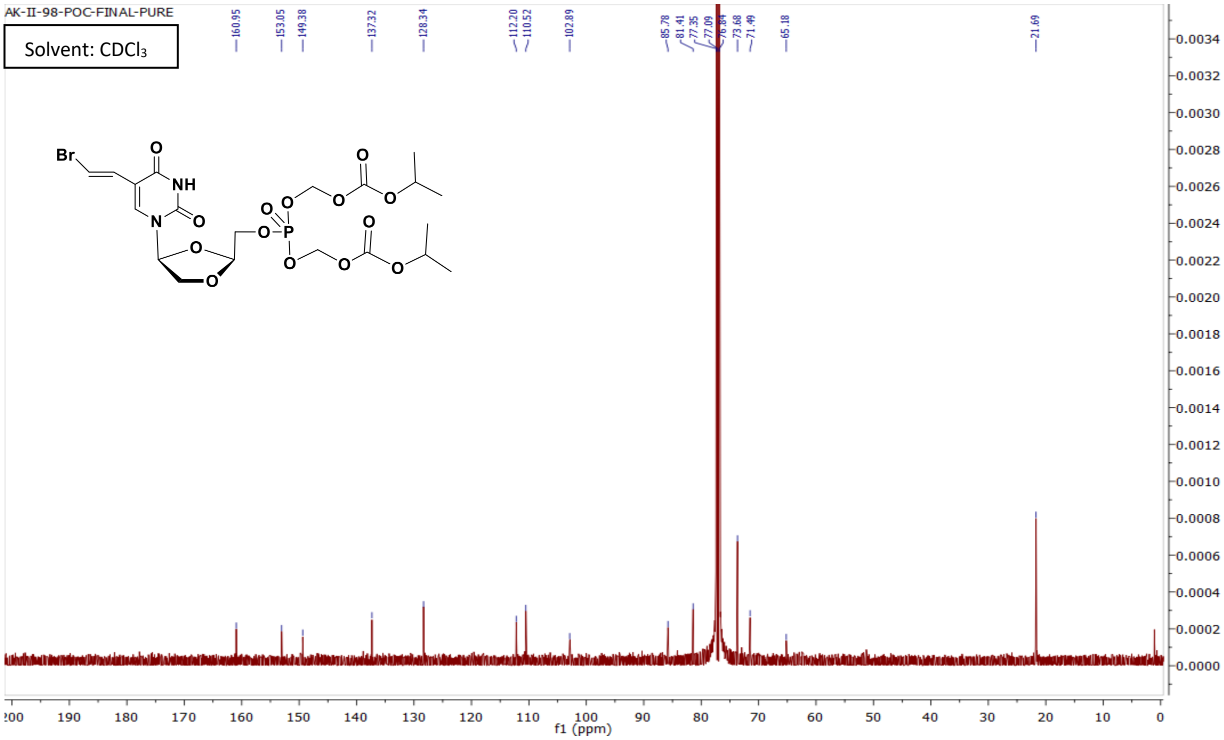

^{13}C NMR of POC-L-BHDU-MP **(47)**.

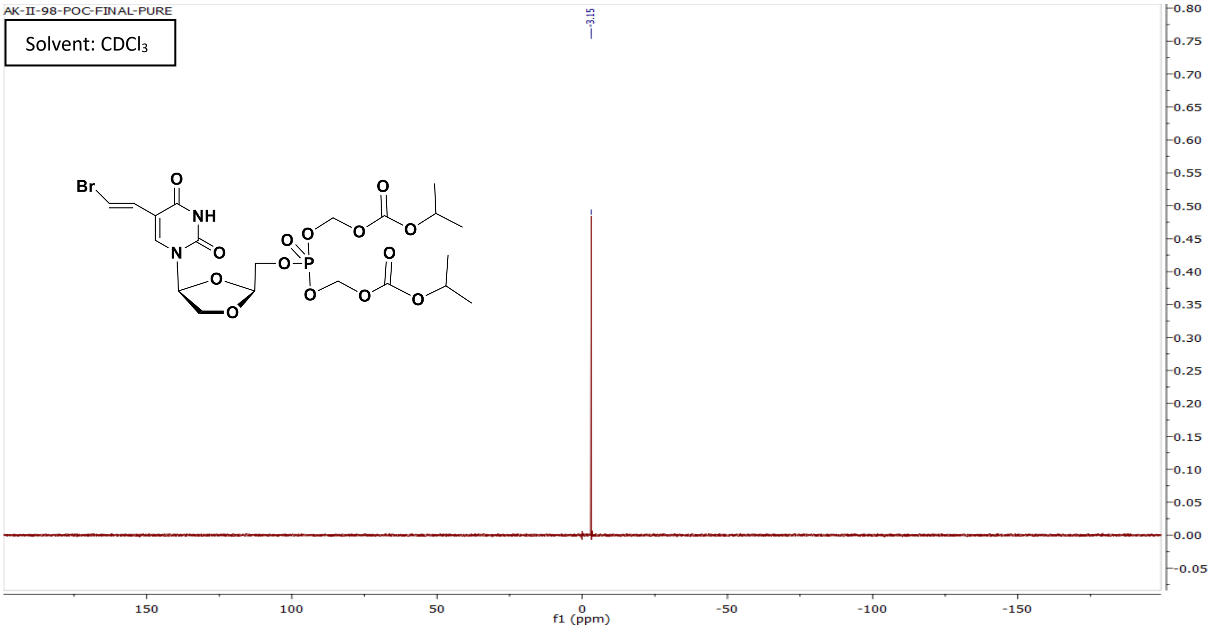

^{31}P NMR of POC-L-BHDU-MP **(47)**.

Table S1: elemental analysis of final compounds

Compound	Molecular Formula	Calculated			Found		
		C	H	N	C	H	N
14.	$C_{12}H_{15}BrClN_3O_6$. 1.5 H_2O	32.78	4.13	9.56	32.39	3.90	9.23
15.	$C_{13}H_{17}BrClN_3O_6$. 1.0 H_2O	35.11	4.31	9.45	35.05	4.32	9.28
16.	$C_{19}H_{21}BrClN_3O_6$. 1.0 H_2O	43.82	4.45	8.07	43.75	4.51	7.98
17.	$C_{15}H_{21}BrClN_3O_6$. 0.5 H_2O	38.85	4.78	9.06	38.82	4.85	8.96
18.	$C_{15}H_{21}BrClN_3O_6$. 1.2 H_2O	37.82	4.95	8.82	37.69	5.12	8.65
19.	$C_{21}H_{22}BrClN_4O_6$. 1.0 H_2O	45.06	4.32	10.0	44.75	4.46	9.78
20.	$C_{16}H_{23}BrClN_3O_6$. 1.0 H_2O	39.48	5.18	8.63	39.29	5.31	8.41
21.	$C_{16}H_{23}BrClN_3O_6$	41.00	4.95	8.96	40.82	4.93	8.74
22.	$C_{15}H_{19}BrClN_3O_6$. 1.0 H_2O	38.28	4.50	8.93	38.30	4.50	8.82
23.	$C_{15}H_{20}BrClN_4O_7$. 1.5 H_2O	35.28	4.54	10.97	35.43	4.63	10.63
24.	$C_{14}H_{19}BrClN_3O_6$. 1.0 H_2O	36.66	4.61	9.16	36.94	4.64	8.77
25.	$C_{16}H_{25}BrCl_2N_4O_6$. 1.5 H_2O	35.12	5.16	10.24	35.26	5.28	9.87
26.	$C_{19}H_{21}BrClN_3O_7$. 1.0 H_2O	42.52	4.32	7.83	42.54	4.42	7.68
33.	$C_{22}H_{27}BrN_3O_9P$	44.91	4.63	7.14	45.23	4.92	6.85
34.	$C_{28}H_{31}BrN_3O_9P$	50.61	4.70	6.32	50.92	4.95	6.11
38.	$C_{30}H_{52}BrN_2O_9P$. 0.5 H_2O	51.14	7.58	3.98	51.20	7.55	3.92
39.	$C_{29}H_{50}BrN_2O_9P$. 0.9 H_2O	49.92	7.48	4.01	49.65	7.62	3.69
41.	$C_{22}H_{32}BrN_3O_{12}P$	42.12	5.14	4.47	42.34	5.23	4.26

HPLC method and trace for compound (1)

HPLC Method Conditions			
Column	XBridge C18 (4.6x150), mm, 3.5µm	Sample	YK-02-22-P
Mobile Phase-A	10mM Ammonium Acetate in Water	Vial	1:F
Mobile Phase-B	100% Acetonitrile	Injection Volume	0.5 µL
Gradient (T% B)	0/10,1/10,10/95,15/95,15.1/10	Run Time	20 minutes
Flow Rate	0.8 mL/min	Proc. Chnl. Descr.:	2998 PDA MaxPlot (190.0 nm to 800.0 nm) (2998(210-400)nm) Subtracted from blank
Column oven Temp	Ambient	Acq. Method Set:	Method_1_COL-2
Diluent: MeOH			

Auto-Scaled Chromatogram

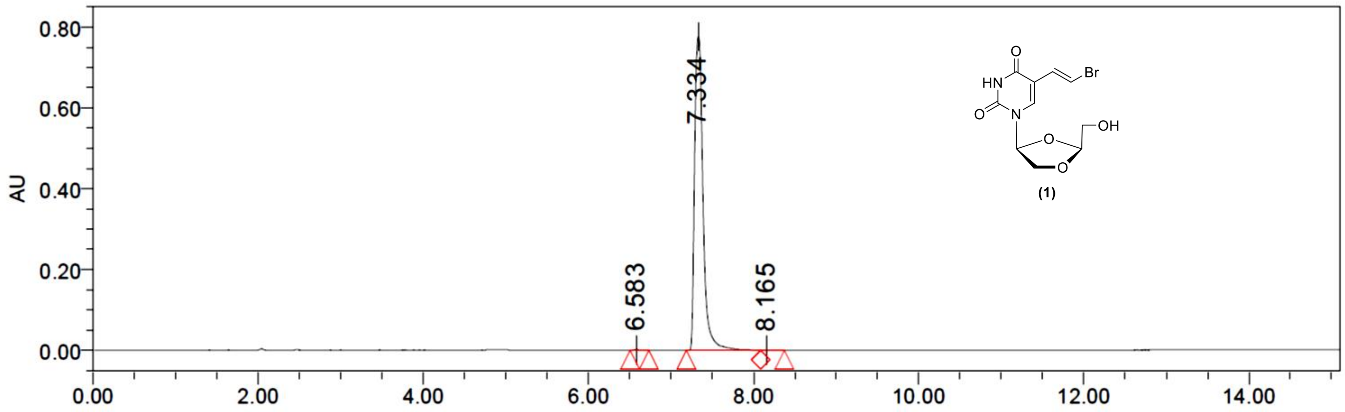

Peak Results

	RT	Area	% Area
1	6.583	3900	0.07
2	7.334	5515706	99.85
3	8.165	4112	0.07

HPLC method and trace for compound (24)

HPLC Method Conditions			
Column	XBridge C18 (4.6x150), mm, 3.5µm	Sample	USS-09-40-26
Mobile Phase-A	10mM Ammonium Acetate in Water	Vial	1:F
Mobile Phase-B	100% Acetonitrile	Injection Volume	0.5 µL
Gradient (T% B)	0/10,1/10,10/95,15/95,15.1/10	Run Time	20 minutes
Flow Rate	0.8 mL/min	Proc. Chnl. Descr.:	2998 PDA MaxPlot (190.0 nm to 800.0 nm) (2998(210-400)nm) Subtracted from blank
Column oven Temp	Ambient	Acq. Method Set:	Method_1_COL-2
Diluent: MeOH			

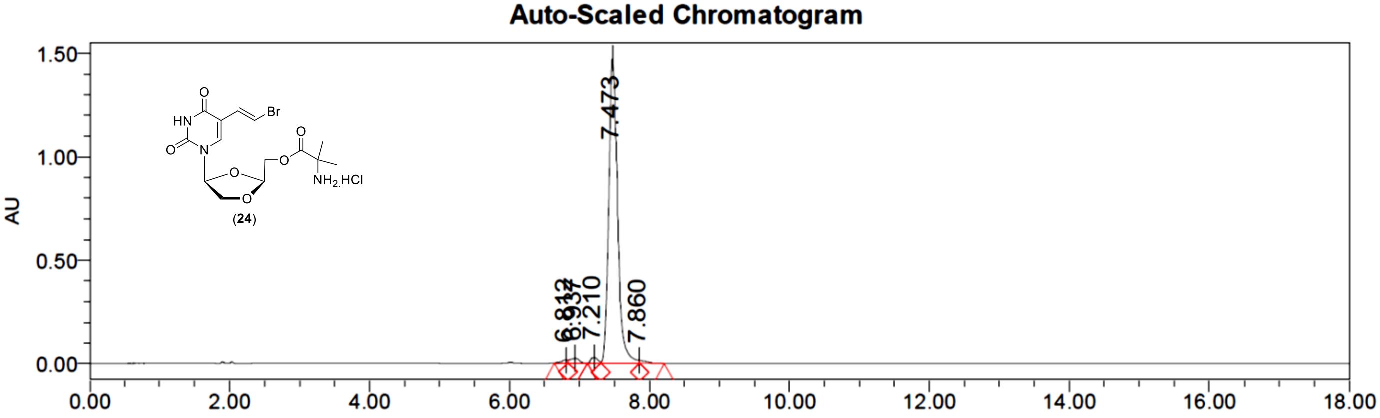

Peak Results

	RT	Area	% Area
1	6.812	89312	0.66
2	6.937	215246	1.60
3	7.210	179665	1.34
4	7.473	12863074	95.63
5	7.860	103434	0.77

HPLC method and trace for compound (34)

HPLC Method Conditions			
Column	UPLC BEH C18 (100x2.1), mm, 1.7µm	Sample	USS-09-67-42
Mobile Phase-A	0.05% FA in water	Vial	2:C,8
Mobile Phase-B	0.05% FA in ACN	Injection Volume	0.5 µL
Gradient (T% B)	0/3, 0.5/3, 3/15, 5/100, 8/100, 8.10/3, 10/3	Run Time	10 minutes
Flow Rate	0.8 mL/min	Proc. Chnl. Descr.:	2998 PDA MaxPlot (190.0 nm to 800.0 nm) (2998(210-400) nm) Subtracted from Blank
Column oven Temp	50 °C	Acq. Method Set:	Method_1_COL-2
Diluent: Acetonitrile (ACN)			

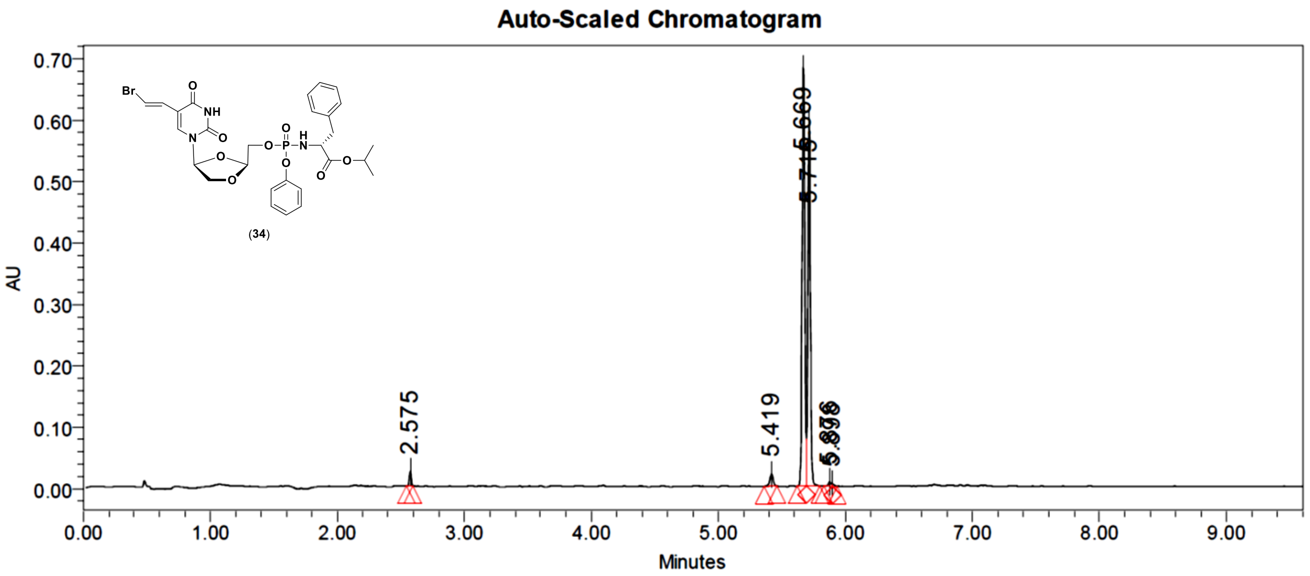

Peak Results

	RT	Area	Height	% Area
1	2.575	24554	23527	1.30
2	5.419	37276	19381	1.97
3	5.669	970842	683177	51.31
4	5.715	844265	596844	44.62
5	5.876	11377	7188	0.60
6	5.898	3622	3512	0.19

Mixture of two diastereomers
Diastereomer 1 = 51.31
Diastereomer 2 = 44.62
Total HPLC purity = 95.93

HPLC method and trace for compound (38)

HPLC Method Conditions			
Column	XBridge C18 (4.6x150), mm, 3.5µm	Sample	YK-01-14-40
Mobile Phase-A	10mM Ammonium Acetate in Water	Vial	1:F
Mobile Phase-B	100% Acetonitrile	Injection Volume	0.5 µL
Gradient (T% B)	0/50,1/50,10/95,20/95, 21/50	Run Time	20 minutes
Flow Rate	0.8 mL/min	Proc. Chnl. Descr.:	2998 PDA MaxPlot (190.0 nm to 800.0 nm) (2998(210-400) nm)
Column oven Temp	Ambient	Acq. Method Set:	Method_1_COL-2
Diluent: MeOH+DMSO			

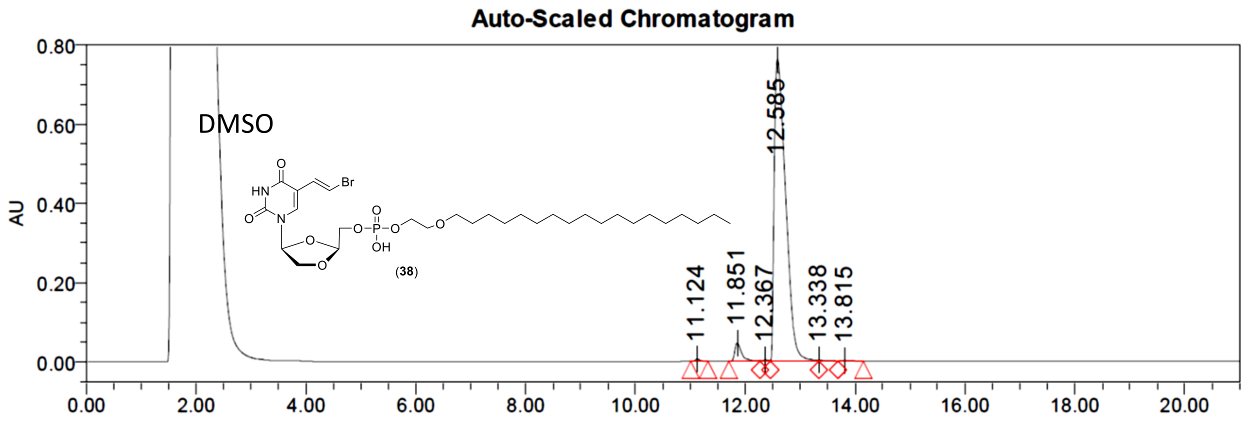

Peak Results

	RT	Area	% Area
1	11.124	32548	0.28
2	11.851	411117	3.58
3	12.367	26936	0.23
4	12.585	10970267	95.52
5	13.338	26873	0.23
6	13.815	17312	0.15

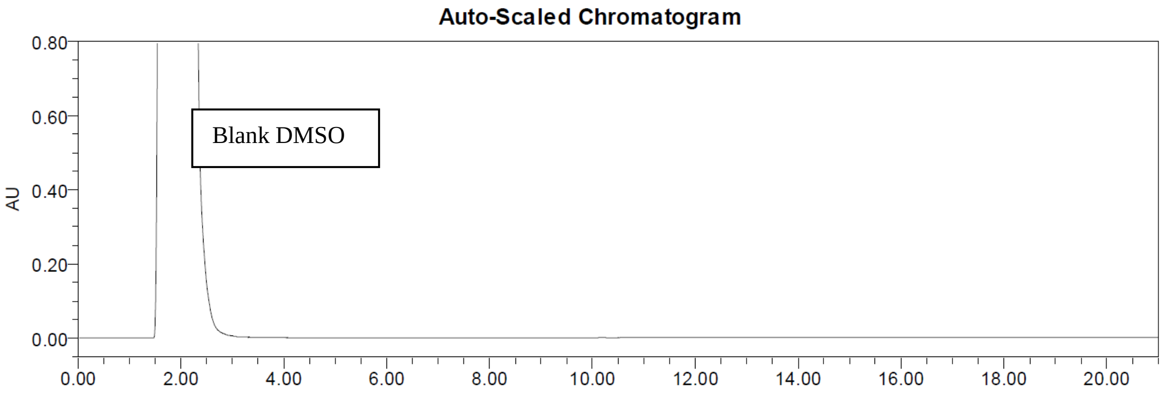

Peak Results

	RT	Area	% Area
1			

HPLC method and trace for compound (39)

HPLC Method Conditions			
Column	XBridge C18 (4.6x150), mm, 3.5µm	Sample	USS-09-07-42
Mobile Phase-A	10mM Ammonium Acetate in Water	Vial	1:F
Mobile Phase-B	100% Acetonitrile	Injection Volume	0.5 µL
Gradient (T% B)	0/50,1/50,10/95,20/95, 21/50	Run Time	20 minute
Flow Rate	0.8 mL/min	Proc. Chnl. Descr.:	2998 PDA MaxPlot (190.0 nm to 800.0 nm) (2998(210-400)nm)
Column oven Temp	Ambient	Acq. Method Set:	Method_1_COL-2
Diluent: MeOH+DMSO			

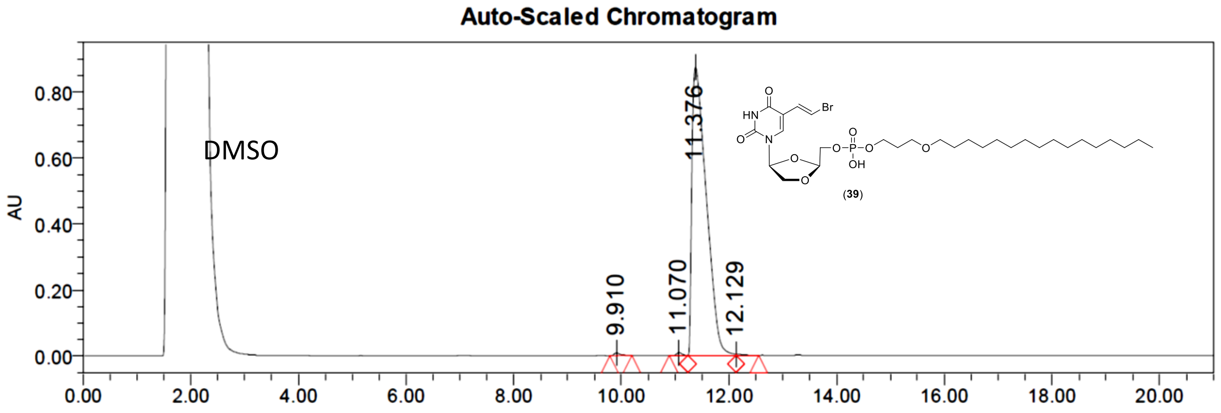

Peak Results

	RT	Area	% Area
1	9.910	70920	0.43
2	11.070	67562	0.41
3	11.376	16151477	98.97
4	12.129	29648	0.18

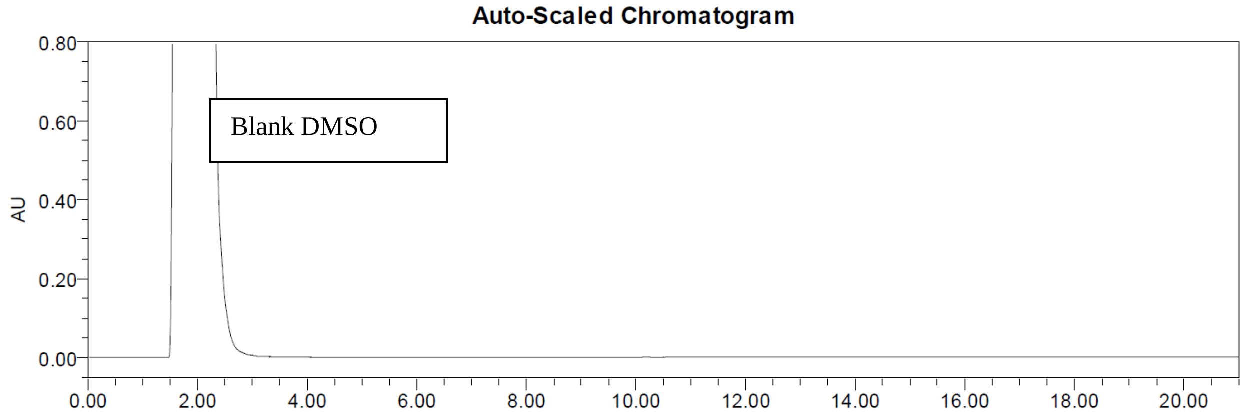

Peak Results

	RT	Area	% Area
1			

HPLC method and trace for compound (41)

HPLC Method Conditions			
Column	XBridge C18 (4.6x150), mm, 3.5µm	Sample	YK-01-16-P-2
Mobile Phase-A	10mM Ammonium Acetate in Water	Vial	1:F
Mobile Phase-B	100% Acetonitrile	Injection Volume	0.5 µL
Gradient (T% B)	0/10,1/10,10/95,15/95. 15. 1/10	Run Time	20 minute
Flow Rate	0.8 mL/min	Proc. Chnl. Descr.:	2998 PDA MaxPlot (190.0 nm to 800.0 nm) (2998(210-400) nm) Subtracted from blank
Column oven Temp	Ambient	Acq. Method Set:	Method_1_COL-2
Diluent: MeOH			

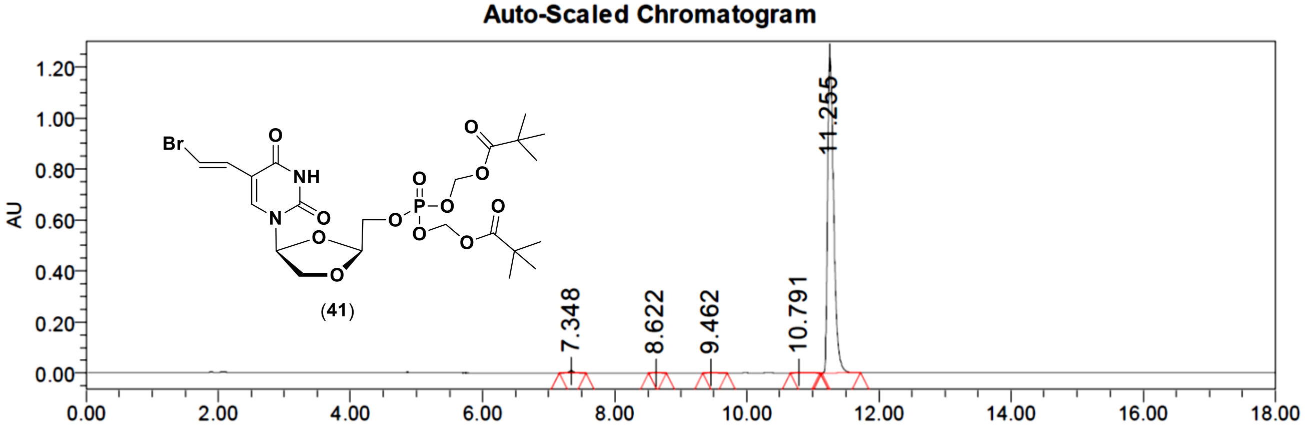

Peak Results

	RT	Area	% Area
1	7.348	44197	0.55
2	8.622	11801	0.15
3	9.462	17042	0.21
4	10.791	24910	0.31
5	11.255	7996494	98.79

HPLC method and trace for compound (47)

HPLC Method Conditions			
Column	XBridge C18 (4.6x150), mm, 3.5µm	Sample	YK-02-26-P
Mobile Phase-A	10mM Ammonium Acetate in water	Vial	1:F
Mobile Phase-B	100% Acetonitrile	Injection Volume	0.5 µL
Gradient (T% B)	0/10,1/10,10/95,15/95. 15. 1/10	Rum Time	20 minute
Flow Rate	0.8 mL/min	Proc. Chnl. Descr.:	2998 PDA MaxPlot (190.0 nm to 800.0 nm) (2998(210-400) nm) Subtracted from blank
Column oven Temp	Ambient	Acq. Method Set:	Method_1_COL-2
Diluent: MeOH			

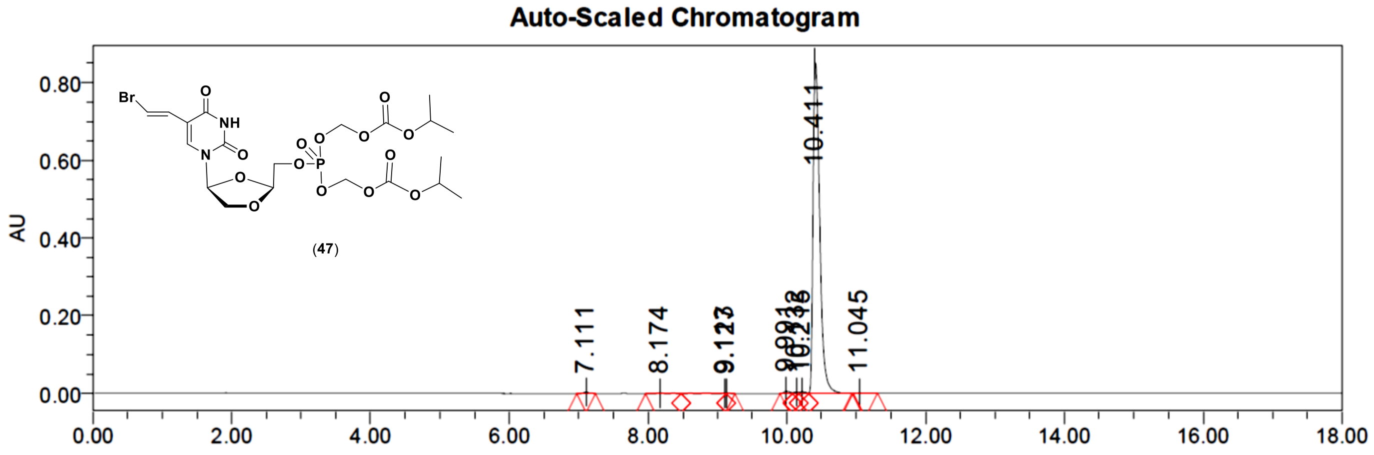

Peak Results

	RT	Area	% Area
1	7.111	16180	0.28
2	8.174	8653	0.15
3	9.113	13805	0.24
4	9.127	3178	0.05
5	9.991	30861	0.53
6	10.132	15077	0.26
7	10.215	24301	0.42
8	10.411	5694194	97.91

	RT	Area	% Area
9	11.045	9239	0.16
